# Computer Vision Helps Experimentally Monitor Mixing Effects in Deep Eutectic Solvents

**DOI:** 10.1021/acssuschemeng.5c05783

**Published:** 2025-10-03

**Authors:** Calum Fyfe, Rhoda Duncan, Timothy J. D. McCabe, Kristin Donnachie, Henry Barrington, Marc Reid

**Affiliations:** Department of Pure and Applied Chemistry, 3527 University of Strathclyde, Glasgow G1 1XL, U.K.

**Keywords:** viscosity, mass transfer, reaction optimization, green chemistry, computational fluid dynamics, imaging, sustainable synthesis, mixing, camera

## Abstract

Deep eutectic solvents (DESs) offer promising sustainable alternatives to petroleum-derived solvents, yet their high viscosities present significant mixing challenges that can impact synthetic outcomes. Here, we demonstrate the application of computer vision as a quantitative, non-invasive tool for monitoring and optimizing mixing in DESs. Using *Kineticolor* video analysis software, we tracked mixing dynamics across three model DES formulations (ChCl/EG, ChCl/G, ChCl/U) under varying temperatures and vessel geometries. Our results reveal that mixing completion times span from seconds (for MeOH) to over 60 min (for a viscous DES), with temperature elevation from 25 to 60 °C reducing mixing times by up to 10-fold. Computational fluid dynamics (CFD) simulations validate experimental observations, showing severe flow field restriction in narrow vessel geometries with highly viscous DES formulations. We demonstrate the practical implications and value of understanding these mixing phenomena through sodium borohydride-mediated aldehyde reduction. This work demonstrates computer vision and video analysis as an essential method for bridging the gap between sustainability goals and practical synthetic implementation when developing methodologies using DES solvents.

## Graphical abstract

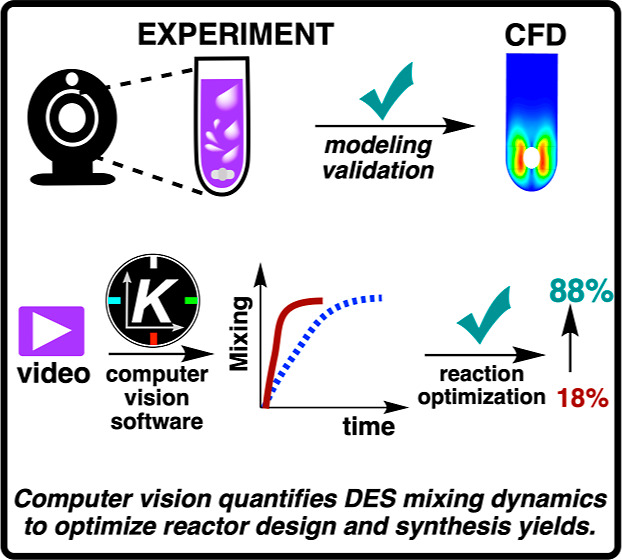

## 1 Introduction

### 1.1 Deep Eutectic Solvents

Deep eutectic solvents (DESs) are a unique class of solvent formed from a hydrogen bond donor (HBD) and a hydrogen bond acceptor (HBA).^1,2^ The resulting viscous mixtures often have melting points significantly lower than their individual components due to extensive hydrogen bonding, enabling them to remain liquid at room temperature.^3^ Common examples include choline chloride with ethylene glycol (ChCl/EG), glycerol (ChCl/G), or urea (ChCl/U), typically in a 1:2 molar ratio (Figure 1).

**Figure 1:**
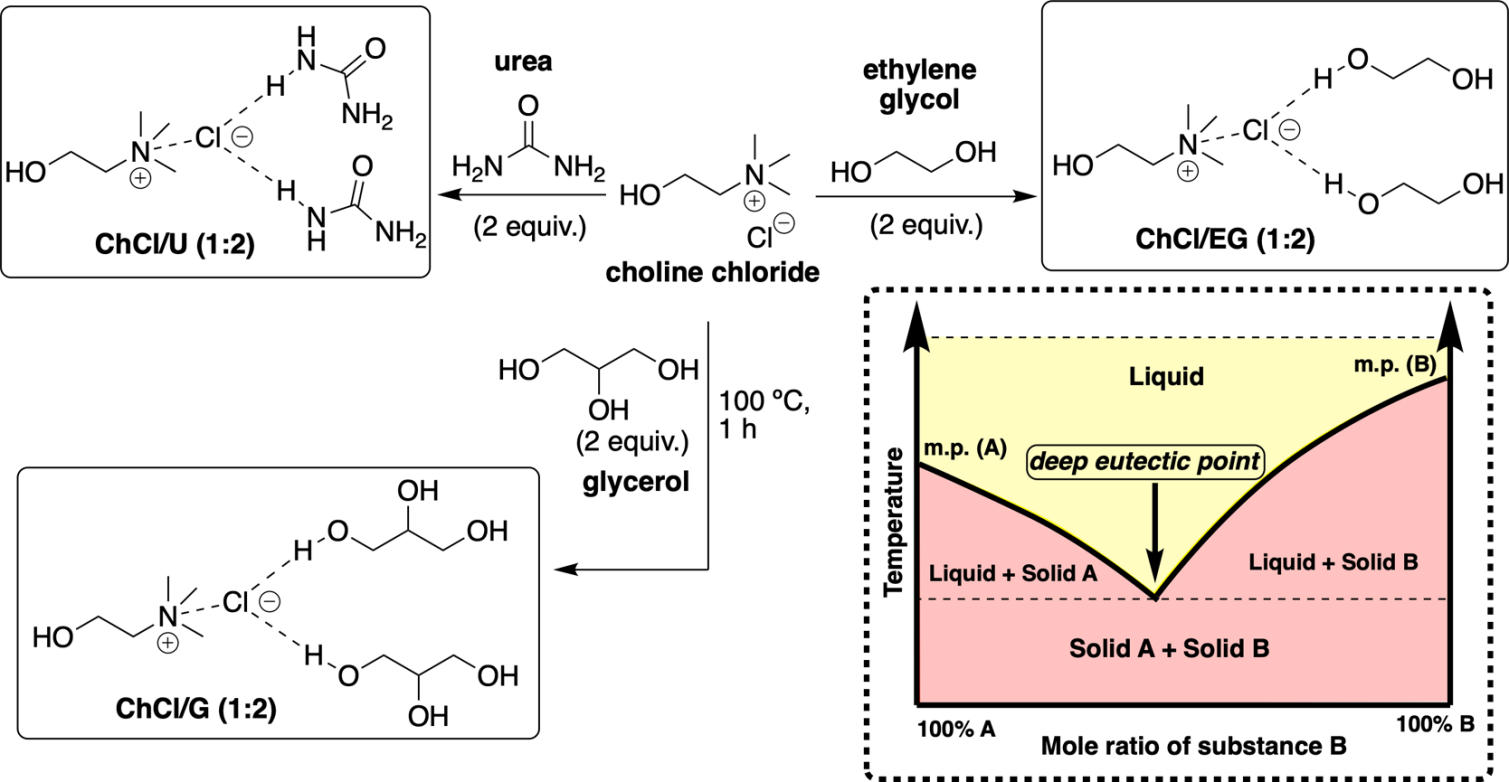
Common DES formulations used in this study. Inset: Physicochemical origins of DES formulations.

DESs have attracted attention—in research and review—as sustainable alternatives to petrochemical solvents^4,5^ due to their biodegradability,^6^ low toxicity, and cost-effectiveness.^2^ They have also become subjects of investigation in a wider range of domains, including extraction of biologics,^7–14^ antimicrobial applications,^15–23^ lubrication,^24–33^ coatings,^34–43^ food safety,^5,44–52^ and synthesis.^53–67^ In synthesis, DESs have demonstrated utility in various processes, including CO_2_ capture,^68–82^ electrophilic aromatic halogenation,^83^ alkylation of amines,^84^ reductions with hydride reagents,^85^ and dipolar cycloadditions.^86^ Additionally, DESs have been used as electrolytes in electrochemical processes.^87,88^ Across this rich array of applications for DESs, the practical adoption of this attractive solvent class in synthetic chemistry should be considered with awareness of their inherently high viscosities (up to 1700 times that of water at 25 °C) which can impede efficient mixing and mass transfer. Being able to easily monitor and quantify these mixing challenges would enable mass transfer limitations and mixing efficiency to more readily feature in the design of chemical reactions compatible with DESs. This goal formed the basis for our investigation.

### 1.2 Viscosity and Mixing Challenges in Deep Eutectic Solvents

The high viscosities and often complex microstructures of DESs pose challenges for mixing and mass transfer. In such viscous media, and in related ionic liquids, flows tend to be laminar, and molecular diffusion is slow, leading to prolonged mixing times compared to conventional solvents.^89,90^ Ionic liquids can have self-diffusion coefficients orders of magnitude lower than water, meaning that unmixed pockets persist longer unless agitation is effective.^89^ Deep eutectic solvents similarly exhibit sluggish diffusion; in extreme cases (e.g., semisolid aerosol droplets of DES), mixing time scales can range from minutes to hundreds of hours even for micron-sized domains.^?^ These slow mixing dynamics can be attributed to the high fluid viscosity and to nanoscale structuring into polar and nonpolar domains that can “trap” diffusing species.^91^ While varying the DES component ratio enables strategic tuning of chemical properties,^1,4,5^ they typically exhibit viscosities greater than water at room temperature,^92^ which limits the mobility and mixing of free species within the solvent.^93,94^

Understanding and improving mixing in ILs and DESs is crucial for their use in chemical reactions, extractions, and process applications. A number of experimental studies have probed how mixing proceeds in these media, measuring mixing times, flow patterns, and mass transfer rates under various conditions. Key findings indicate that mixing in ILs and DESs is often diffusion-limited in the absence of strong convection, but innovative mixing strategies (e.g., microfluidic droplet flows, acoustic agitation, or tailored impeller designs) can significantly enhance mixing even in laminar regimes.^89,90^ Raising temperature or adding small amounts of water can lower viscosity and accelerate diffusion by an order of magnitude or more.^95,96^ In IL–water mixtures, water addition similarly boosts ionic mobility and mixing rates.^97^

The impact of viscosity on reaction kinetics can be understood through Kramers’ theory, which extends transition state theory to account for frictional effects in viscous media.^98,99^ Kramers’ rate constant expression for reactions in viscous media is given by

1 $$k=\frac{\omega_{b}}{2\pi \gamma }e^{-\Delta V/k_{B}T}$$

where ω_b_ is the attempt frequency at the top of the barrier, γ is the friction coefficient related to medium viscosity, Δ*V* is the height of the energy barrier, *k*_B_ is the Boltzmann constant, and *T* is the temperature. This framework demonstrates that reaction rates can decrease with increasing medium viscosity through frictional barriers that impede reacting particles from reaching the product state (Figure 2). In practical terms, high viscosity impairs both molecular diffusion and bulk convection, particularly under low-shear mixing conditions. The diffusion and turbulence factors that dominate mixing are severely restricted in viscous mixtures.^100^ Increased viscosity raises the friction between molecules, requiring greater shear stress to overcome frictional forces and achieve effective mixing.

**Figure 2:**
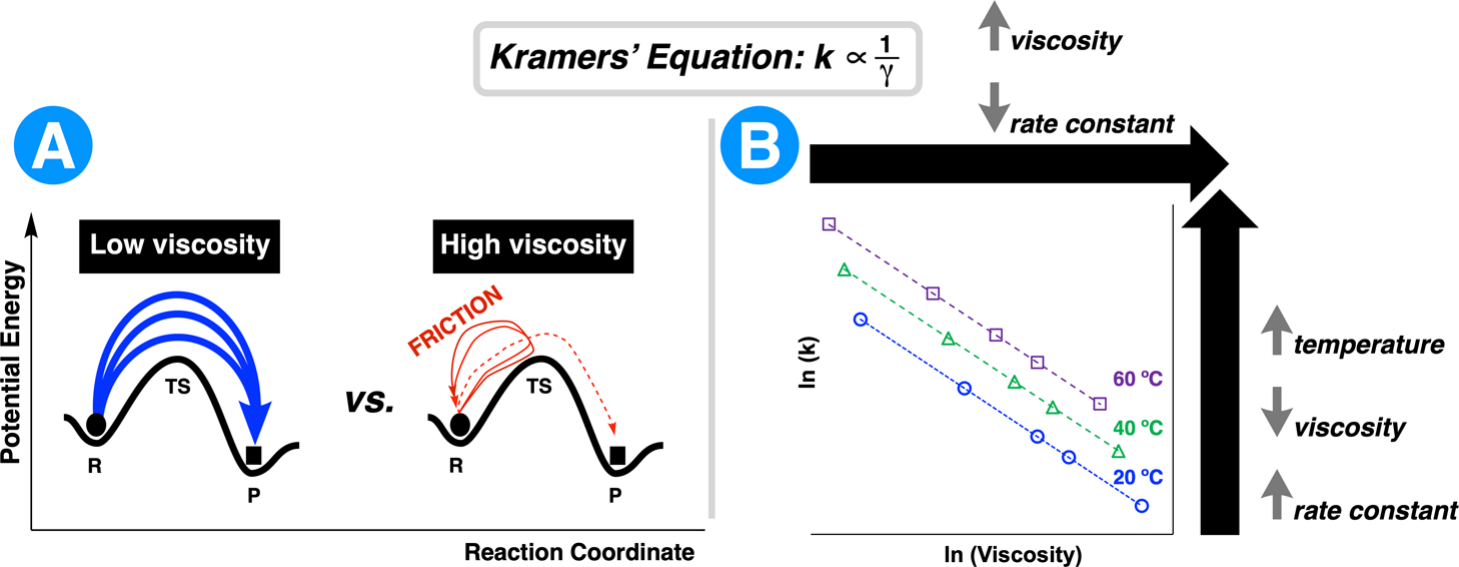
Kramers’ theory explains viscosity-dependent reaction kinetics in deep eutectic solvents. (A) Potential energy diagrams illustrating how medium viscosity affects molecular motion over reaction barriers. In low-viscosity media, reactants easily traverse the transition state (TS) with minimal friction. In high-viscosity media, increased friction creates additional barriers to molecular motion, requiring multiple attempts to cross the same energetic barrier. (B) The inverse relationship between viscosity and reaction rate constant for DES systems at different temperatures (e.g., 20, 40, and 60 °C). The log–log plot demonstrates Kramers’ prediction that rate constants decrease with increasing viscosity, while elevated temperature provides a practical solution by reducing DES viscosity.

Efficient mixing in viscous systems requires strategies that maximize interfacial surface area and ensure uniform distribution throughout the reactor volume.^100^ These requirements necessitate careful consideration of vessel geometry, impeller design, and operating temperature. Failure to account for these parameters can lead to stratification, localized concentration gradients, and ultimately compromised reaction performance.

### 1.3 Analytical Approaches to Assessing Mass Transfer in DESs

The extreme viscosities of DES systems often necessitate specialized analytical approaches to characterize transport phenomena. Controlled studies of liquid–liquid extraction in millireactors reveal the molecular basis for these transport limitations. Mitar et al.^101^ provided direct measurements of volumetric mass transfer coefficients (*K*_r_·*a*) showing strong inverse correlation between DES viscosity and extraction efficiency. Their systematic investigation revealed diffusion coefficients in DESs of 10^–12^ to 10^–13^ m^2^/s, significantly lower than conventional solvents at 10^–9^ to 10^–10^ m^2^/s, providing quantitative evidence for the restricted molecular mobility that creates persistent concentration gradients in viscous DES media.

Pulsed field gradient NMR has emerged as a particularly valuable technique for measuring self-diffusion coefficients directly. D’Agostino et al.^102^ conducted pioneering studies revealing self-diffusion coefficients of 10^–10^ to 10^–13^ m^2^/s in maline (ChCl-malonic acid) systems, demonstrating the technique’s capability to correlate diffusion and viscosity data for understanding mass transfer mechanisms at the molecular level. However, conventional analytical methods can require modification for viscous DES characterization. Magic-angle spinning NMR and extended acquisition times become necessary as standard NMR approaches fail due to slow molecular tumbling in high-viscosity media.^103^ Similarly, differential scanning calorimetry reveals that many DES systems undergo glass transitions rather than crystallization due to kinetic limitations from high viscosity, fundamentally altering thermal analysis protocols compared to conventional solvents.^104^ Beyond NMR, complementary spectroscopic approaches show particular promise for real-time monitoring of DES mixing processes. Raman spectroscopy studies by Elderderi et al.^105^ demonstrated in situ monitoring of DES structural changes with water addition, revealing how hydrogen bond disruption correlates with viscosity reduction and improved transport properties. These noninvasive techniques offer advantages over probe-based methods that can disrupt flow patterns in viscous systems.

Computational approaches successfully capture the complex interplay between structure and transport in DES systems. Molecular dynamics simulations provide detailed understanding of how extensive hydrogen bonding networks create the transport limitations observed experimentally. Bonab et al.^106^ demonstrated through MD simulations that viscosity-dependent self-diffusion coefficients in Type III DESs decrease from 1.52 to 1.07 × 10^–12^ m^2^/s as temperature decreases from 400 to 293 K, revealing the molecular basis for the temperature-dependent mixing improvements observed in reactor studies. Zhang et al.^107^ combined ab initio MD with experimental validation to model density, diffusivity, and viscosity relationships in choline chloride/ethylene glycol systems. Their computational framework revealed dynamic heterogeneities characteristic of viscosity-limited transport, providing molecular-level validation for experimental observations of reduced mass transfer rates and persistent concentration gradients. COSMO-RS thermodynamic modeling by Alnashef et al.^108^ developed quantitative structure–property relationships demonstrating that viscosity significantly influences hydrogen bonding between anions and hydrogen bond donor molecules, directly affecting mass transfer rates. These computational approaches enable prediction of transport limitations and provide design guidance for optimizing DES compositions to balance viscosity with solvent performance—insights directly relevant to reactor design and mixing optimization.

### 1.4 Computer Vision for Mixing Analysis

Traditional mixing diagnostics (including pH probes and torque measurements from overhead stirrers) are invasive and not spatially resolved. Our team has focused on the development of a computer vision software, *Kineticolor*, that enables users to input video footage and output color tracking and mixing analyses. Anyone with any camera can thus gain time- and spatially resolved reaction monitoring insights in a non-invasive and economically accessible manner.^109,110^ With regards mixing, some of our previous studies focused on the implementation and application of computer vision algorithms that would enable computer vision mixing analysis (Figure 3).^111,112^ These efforts were considered against other analytical means by which to monitor mixing, including via pH probe and NMR kinetics of mixing-sensitive reactions. However, to date, the mixing analytics in *Kineticolor* have not been applied to analysis of viscous mixtures.

**Figure 3:**
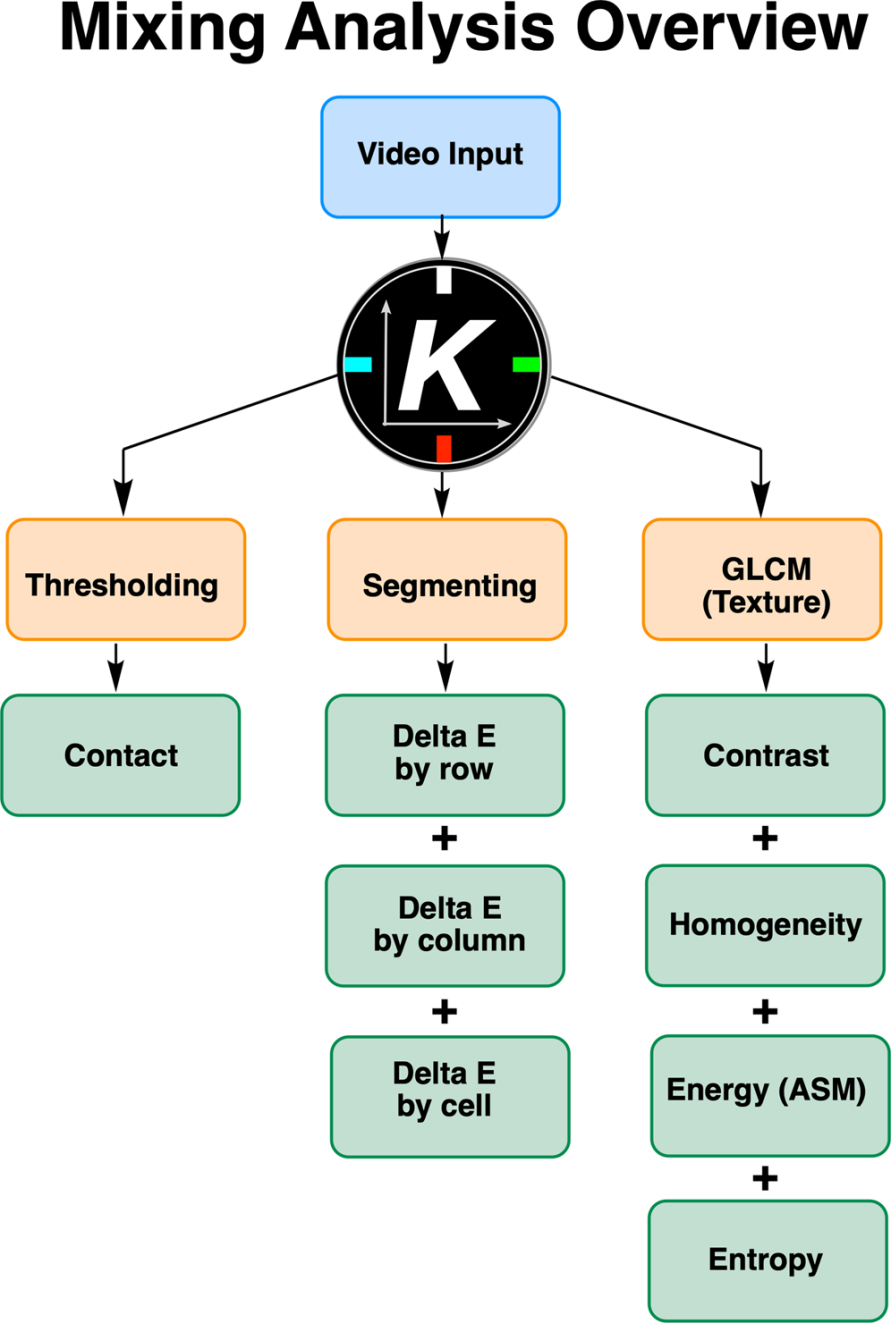
Overview of the computer vision workflow used to analyze mixing effects.

### 1.5 Study Aims

In response to the mixing challenges identified for viscous DES formulations, and building on our earlier mixing studies,^111,112^ we aimed to explore to what extent computer vision analysis could provide quantitative assessment of mixing phenomena across different vessel scales and exemplar DES compositions (Figure 4). Using *Kineticolor* as our method of turning video cameras into non-invasive analytical tools capable of quantifying mixing efficiency in DES systems, our ultimate goal drives at enabling synthesis scale-up decisions without disrupting flow patterns through probe insertion.

**Figure 4:**
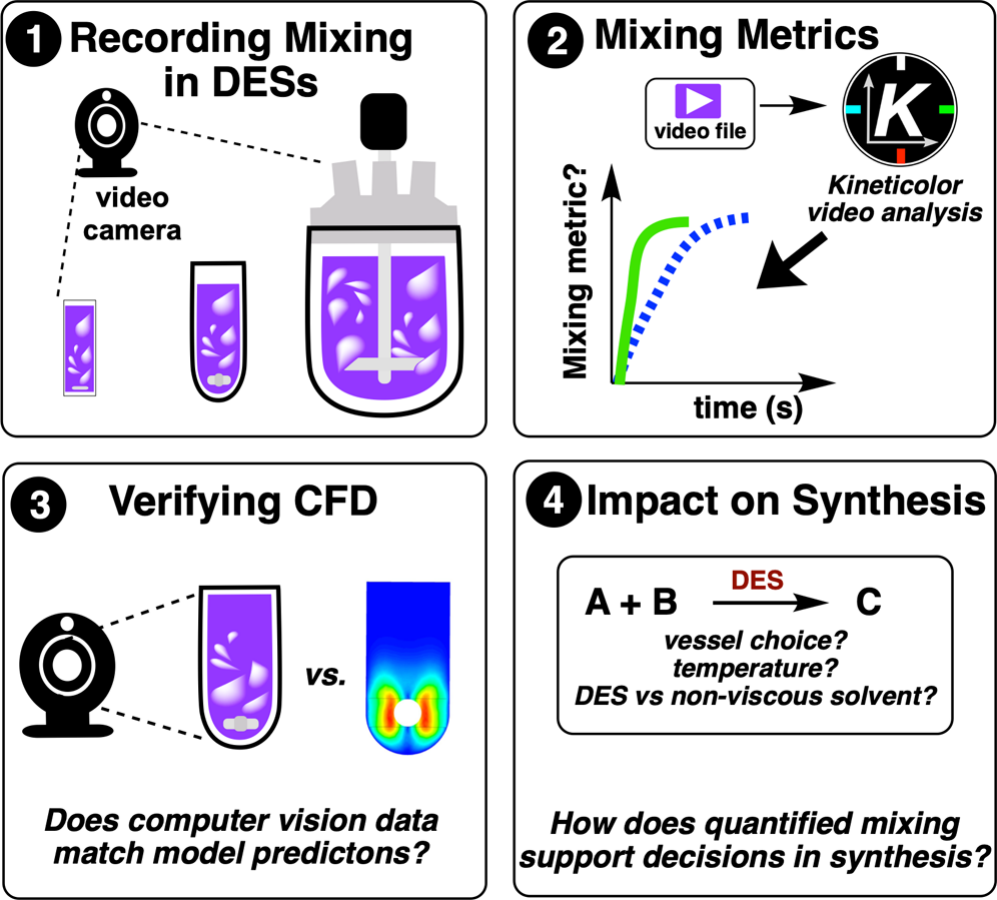
Overview of computer vision approach for analyzing mixing in deep eutectic solvents (DESs). Video recordings of colorimetric reactions are processed using *Kineticolor* software to extract quantitative mixing metrics, which can be used to verify CFD models and inform synthesis decisions regarding vessel design and solvent selection.

More specifically, we sought to

1. Systematically compare mixing dynamics across multiple vessel geometries, different DES viscosities, and a range of temperatures.
2. Identify which computer vision metrics (e.g., Δ*E*, Contact, texture-based parameters) are most sensitive to mixing phenomena in viscous media, and most intuitive with regards user interpretation.
3. Validate the approach using model reaction systems (pH titrations) and real DES-based chemical reactions where mixing affects yield.

While our previous studies established computer vision methods for analyzing mixing in conventional solvents under turbulent flow regimes,^111,112^ DES formulations present fundamentally different challenges. The extreme viscosities of DES (up to 1000× that of water) drive a transition from turbulent to laminar flow regimes, severely restricting mixing zones to regions immediately adjacent to the stirrer bar and creating persistent vertical stratification phenomena invisible to traditional analytical methods. These viscosity-induced limitations result in mixing time variations spanning 4 orders of magnitude—from seconds, far beyond any mixing phenomena studied with *Kineticolor* in our earlier work.

## 2 Results and Discussion

### 2.1 Proof-of-Concept—Tracking Mixing Effects in Viscous Media

Before analyzing any video recordings of processes involving DESs, we first analyzed a viscous variant of a phenolphthalein titration, used previously to develop the mixing metrics now integrated in *Kineticolor*.^111^ We analyzed archival footage, provided by our industrial collaborator during our earlier mixing investigation,^111^ of the two phenolphthalein titrations, one in water and the other in a more viscous water/glycol mixture. First, mixing effects were captured through the color-agnostic metric, Δ*E* (eq 2, Figure 5) which captures contrast between colors rather than specific colors (Figure 6).

2 $$\Delta E_{1976}=\sqrt{{\left(L_{2}^{*}-L_{1}^{*}\right)}^{2}+{\left(a_{2}^{*}-a_{1}^{*}\right)}^{2}+{\left(b_{2}^{*}-b_{1}^{*}\right)}^{2}}$$

- Δ*E* < 1: Change not perceptible by human eye.
- 1 ≤ Δ*E* ≤ 2: Perceptible change with close observation.
- 2 < Δ*E* ≤ 10: Perceptible change at a glance.
- 11 ≤ Δ*E* ≤ 49: Color changes are more similar than opposite.
- 50 ≤ Δ*E* ≤ 99: Colors changes are increasingly obvious.
- Δ*E* ≥ 100: Colors changes are very obvious and stark.

**Figure 5:**
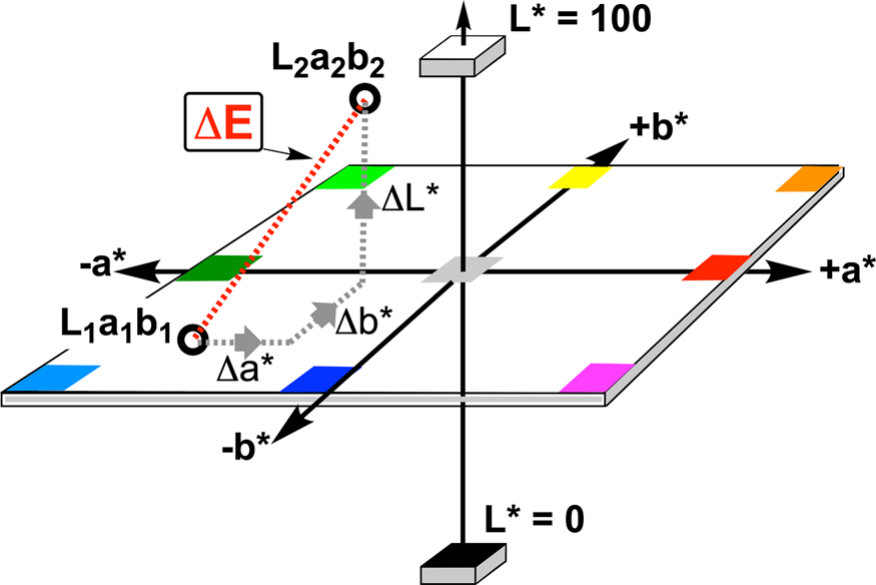
Illustration of Δ*E* calculation as the Euclidean distance between two points in the CIE-L*a*b* color space. The three-dimensional coordinate system shows the L* axis (lightness, 0–100), a* axis (green to red), and b* axis (blue to yellow). Δ*E* represents the straight-line distance between color points *L*_1_ *a*_1_ *b*_1_ and *L*_2_ *a*_2_ *b*_2_, calculated from the coordinate differences ΔL*, Δa*, and Δb*.

**Figure 6:**
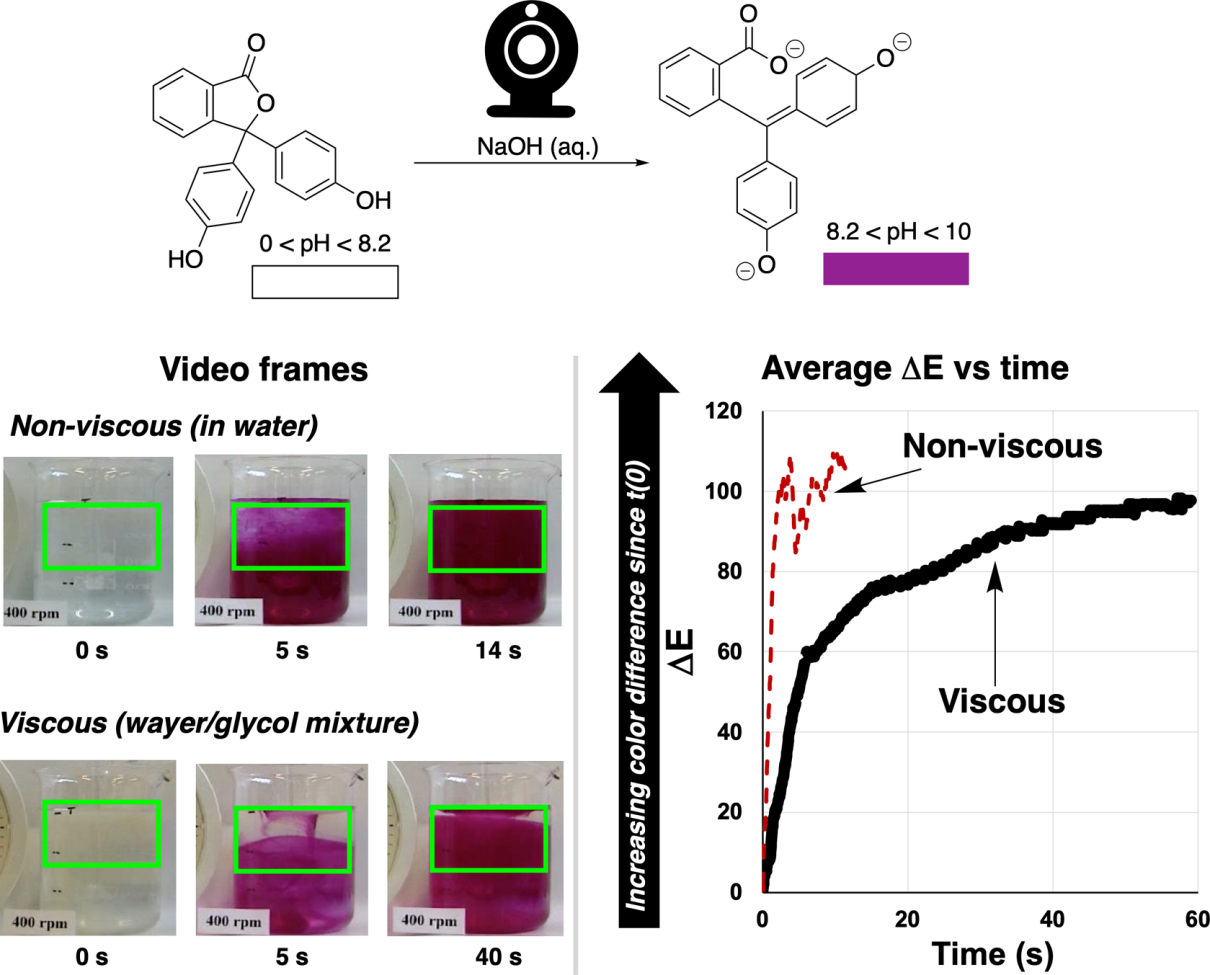
pH titrations with phenolphthalein (acidic to basic) colorimetrically demonstrating the influence of fluid viscosity on mixing. Left: Stills of increasing purple coloration for the reaction in water and in a water/glycol mixed solvent system. Green traces overlaid on images represent selected regions of interest analyzed by *Kineticolor*. Right: Averaged Δ*E* over time for each process, showing the slower rate of change for the viscous mixture.

A host of alternative mixing metrics derived from the video data and computer vision algorithms implemented in *Kineticolor* can also be used to explore the difference in bulk mixing for the viscous and nonviscous reaction mixtures. These metrics spatially analyze the input video, frame by frame, condensing pixel-resolved relationships down into a single value versus time. From previous work, we found that, among a host of such metrics, the *Contact* metric struck the best balance between spatial resolution and ease of interpretation.^111^ Figure 8 shows how *Contact* can be used to succinctly estimate that the viscous phenolphthalein titration takes approximately five times longer to reach the same level of homogeneity as achieved in the nonviscous (water-based) variant of the reaction. A host of additional, more abstract mixing metrics derived from texture analysis algorithms are available and discussed in the Supporting Information. Altogether, this proof-of-concept served to demonstrate that mixing differences in reaction mixtures of differing bulk viscosity could be quantified using various time- and spatially resolved computer vision metrics. Next, our attention turned to applying this learning in the DES context.

**Figure 7:**
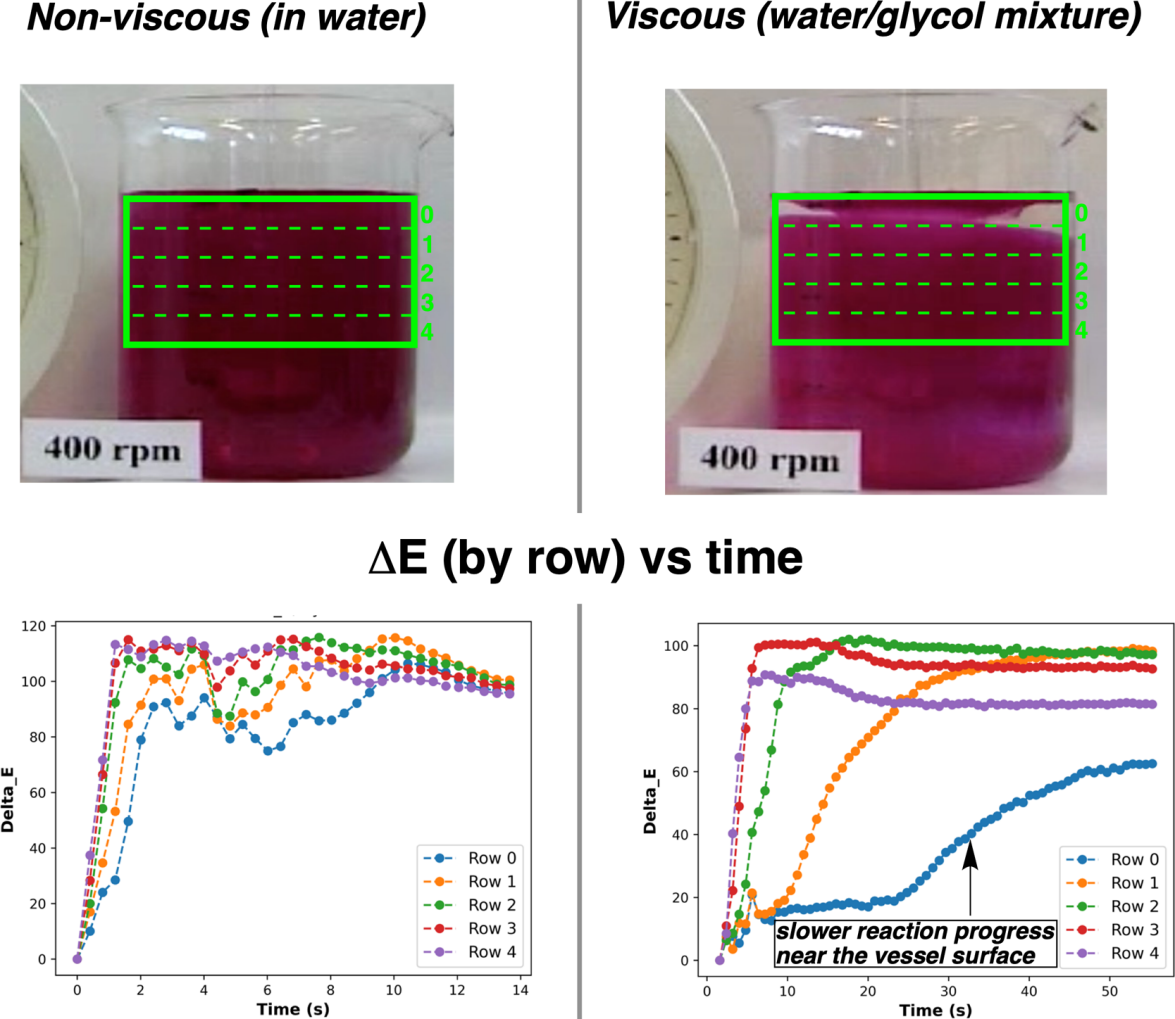
Analysis of phenolphthalein (acidic to basic) pH titrations, exemplifying spatially resolved Δ*E* measurements. Splitting the measurement into five equally spaced rows within the larger ROI highlights the slower rate of reaction progress at the liquid surface (row zero) in the viscous medium (right) versus the nonviscous aqueous medium (left). The still frames shown come from near the end of each video.

**Figure 8:**
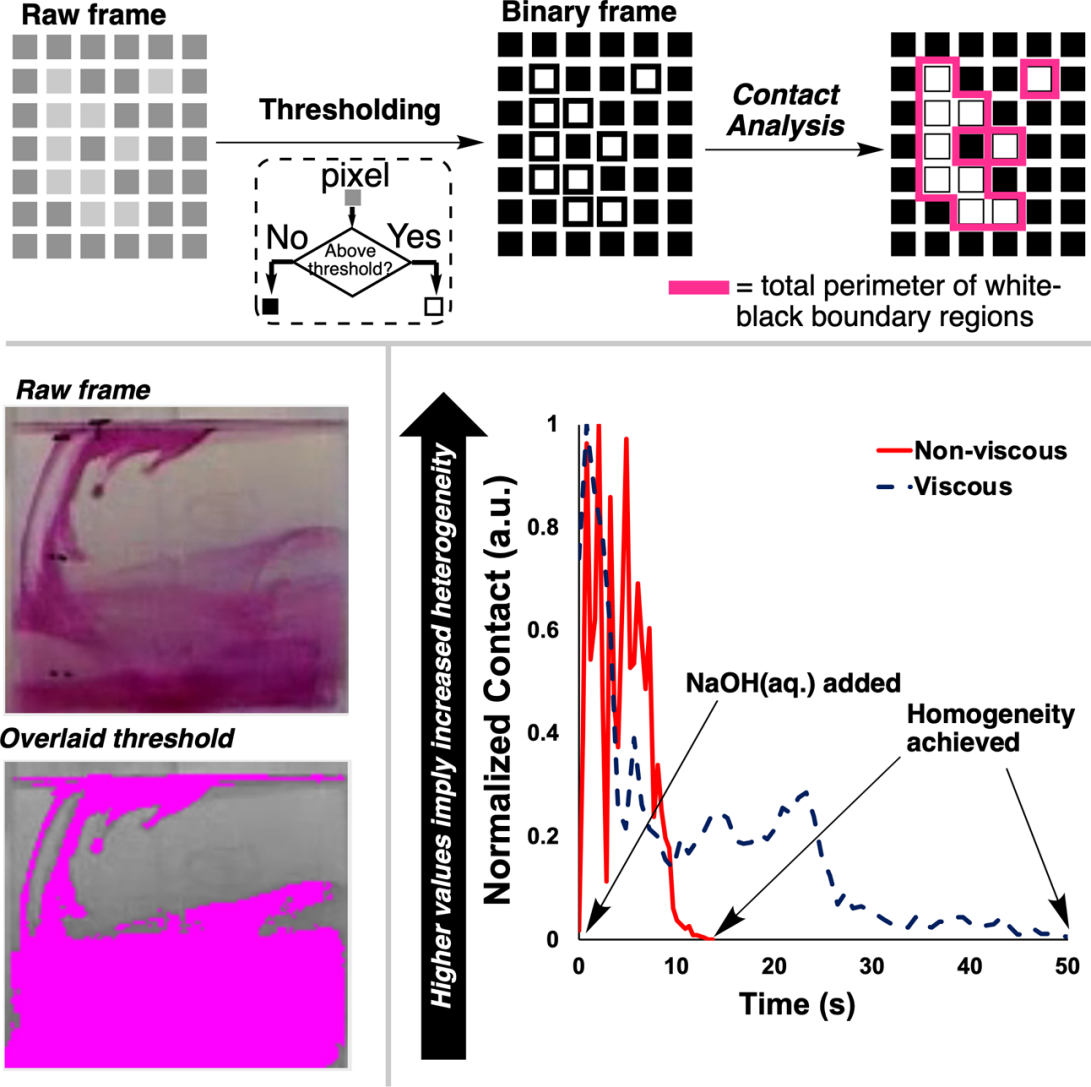
Top: A cartoonized representation of the calculation of *Contact* from video frames. Bottom left: Still from *Kineticolor* showing the overlay of a thresholded region of pixels. Highlighted pixels lie in the greyscale range of 0 – 110. Pixel values above 110 and less than or equal to 255 are not colored. Bottom right: Resulting *Contact* profiles for the viscous and nonviscous phenolphthalein titrations, where the former takes five times longer to reach homogeneity than the latter.

### 2.2 Viscosity Measurements

We began our DES-focused investigation by measuring the viscosity of the selected DESs as a function of temperature (Figure 9). For the available comparisons at 20 °C, our measurements fell within 2–8% of literature values, and with the same qualitative order of viscosity: ChCl/EG < ChCl/G < ChCl/U.

**Figure 9:**
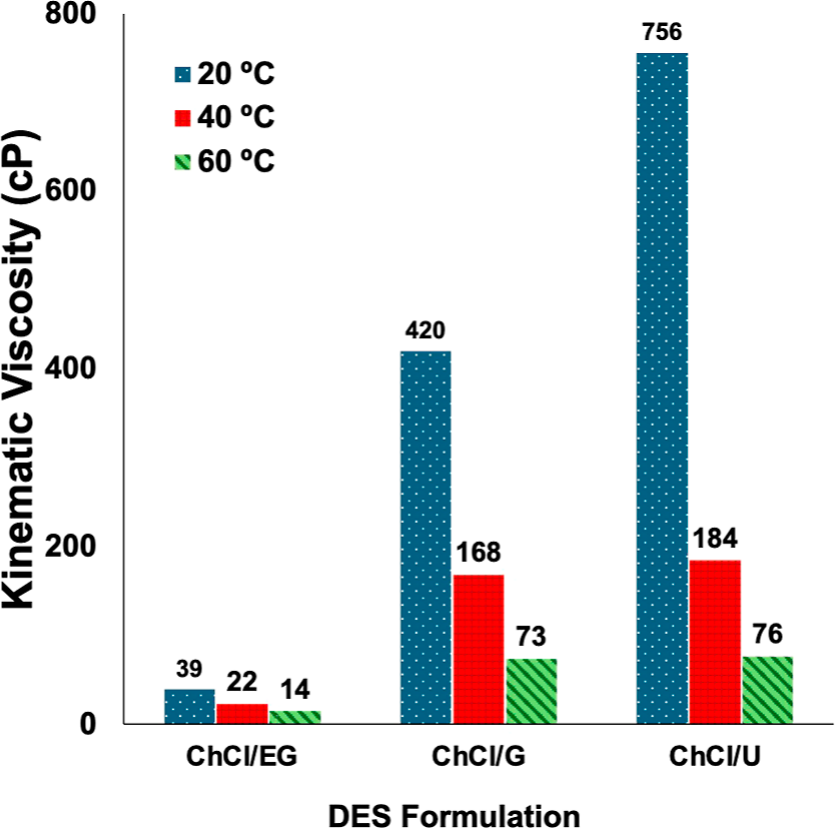
Measured kinematic DES viscosities at various temperatures. The data reveal the following viscosity trend across the tested temperature range: ChCl/EG < ChCl/G < ChCl/U.

These measurements served as an appropriate validation measurement and intuitive aid in interpreting our subsequent computer vision measurements of the mixing dynamics in each DES (see below).

### 2.3 Dye Mixing Measurements with DESs: Cuvette Studies

To establish a baseline understanding of mixing dynamics in DES formulations, we conducted systematic cuvette experiments with all three DES chosen formulations at multiple temperatures. We added 0.1 mL of dye-containing DES to 2.9 mL of DES under constant stirring (500 rpm), video recording the mixing process for 1 h. This methodology provided controlled conditions for quantifying temperature-dependent mixing behavior across the range of DES viscosities.

Indeed, the fact that cameras are somewhat scale-agnostic enables detailed mixing analysis even in small-scale systems. The unique advantage of computer vision methods becomes clear when considering that individual pixels act as distributed “probes” across the entire field of view. In a 1 cm cuvette captured at 1920 × 1080 pixel resolution, for example, one effectively obtains thousands of measurement points with submillimeter spatial resolution. Each pixel captures local concentration gradients through color changes, revealing microscale heterogeneity and meso-mixing patterns that would be invisible to traditional single-point probes.

The high spatial resolution afforded by cameras is particularly valuable for viscous fluids like DESs, where local concentration gradients can persist significantly longer than in low-viscosity systems they may be replacing. The slow dynamics in DESs means that even small-scale experiments can exhibit distinct spatial patterns (i.e., vortices, dead zones, and stratification) that are typically associated with larger vessels. Thus, computer vision transforms the apparent limitation of small reaction volumes into an advantage: the ability to observe and quantify mixing phenomena with unprecedented detail across multiple length scales simultaneously, from molecular-level color changes to system-wide flow patterns.

### 2.4 Temperature-Dependent Mixing Dynamics

Our computer vision analysis revealed distinct mixing profiles for each DES-temperature combination (Table 1). At 25 °C, the ChCl/U DES showed no detectable plateau after 1 h of mixing, indicating incomplete homogenization. The ChCl/G DES similarly failed to reach complete mixing within the experimental time frame. In contrast, the less viscous ChCl/EG DES achieved homogeneity with a mean plateau time of 850 ± 315 s. The large standard deviations in plateau times reflect the stochastic nature of mixing in highly viscous media, where local concentration gradients can persist for extended periods.

**Table 1:**
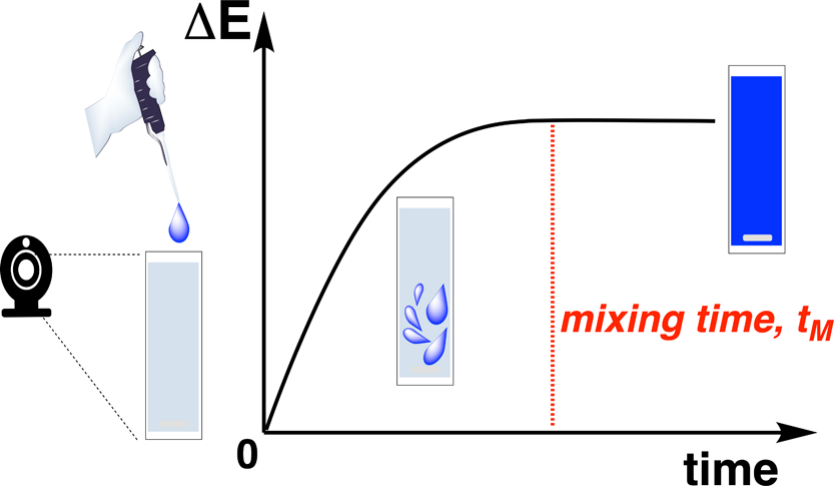
Impact of Temperature and Viscosity on Mixing Plateau Times in DES Formulations and Methanol^a^

solvent (*T*, °C)	viscosity (cP)	mean mixing time (s)	std dev mixing time (s)
MeOH(40)	0.456	12.7	2.08
MeOH(25)	0.544	16.7	6.66
ChCl/EG(60)	13	260	75.5
ZnCl_2_/U(60)		340	69
ChCl/G(60)	54	640	96.4
ChCl/EG(40)	24	710	251.6
ChCl/EG(25)	48	850	315.1
ChCl/U(60)	69	860	105.4
Glycerol(60)	80	970	181
ChCl/U(40)	238	1770	425.3
ChCl/G(40)	133	1777.5	432.9
ChCl/G(25)	281	no plateau	
ChCl/U(25)	750	no plateau	
ChCl/ZnCl_2_(60)^b^		no plateau	
ZnCl_2_/U(25)		no plateau	

a Stirring rate: 500 rpm Stirrer geometry: 6 mm × 3 mm.

b 1:3.5 composition instead of 1:2.

Temperature had a profound effect on mixing times across all DES formulations studied. At 40 °C, both ChCl/G and ChCl/U DES achieved mixing plateaus at approximately 1770 s, while ChCl/EG mixed in 710 ± 252 s. At 60 °C, all DES formulations showed dramatically improved mixing, with ChCl/G and ChCl/U reaching plateaus at 640 ± 96 and 860 ± 105 s respectively, while ChCl/EG mixed in just 260 ± 76 s.

For comparison, methanol at both 25 and 40 °C achieved complete mixing in 16.7 ± 6.7 and 12.7 ± 2.1 s, respectively, highlighting the orders-of-magnitude difference in mixing time scales between conventional solvents and DES. Interestingly, comparison of the ChCl/G DES to its pure glycerol component showed that the latter evidenced a 1.5 times slower mixing time, demonstrating the ability to distinguish unique mixing features of the DES mixing versus a component of that DES. Going further, we ended this study with a brief expansion to investigate mixing times in Type I (e.g., ChCl/ZnCl_2_) and Type IV (e.g., ZnCl_2_/U) DES formulations, both of which were qualitatively much more viscous than any of the Type III DES formulations on which the majority of this study is focused.

### 2.5 Spatially Resolved Mixing Analysis

Building on the proof-of-concept analysis first shown in Figure 7, row-by-row Δ*E* analysis provided spatial insight into the mixing process (Figure 10). In highly viscous DES at 25 °C, the dye initially concentrated in the upper regions of the cuvette (Row 0) before gradually dispersing downward. This vertical concentration gradient persisted for extended periods in ChCl/G and ChCl/U formulations, indicating limited convective transport. As temperature increased, the row-specific Δ*E* profiles converged more rapidly, demonstrating improved mass transport throughout the vessel volume. The spatial resolution enabled by the row-segmented Δ*E* measurements revealed persistent vertical stratification in highly viscous DES formulations, demonstrating the utility of computer vision for quantifying mass transport limitations in viscous media, even on the small scale.

**Figure 10:**
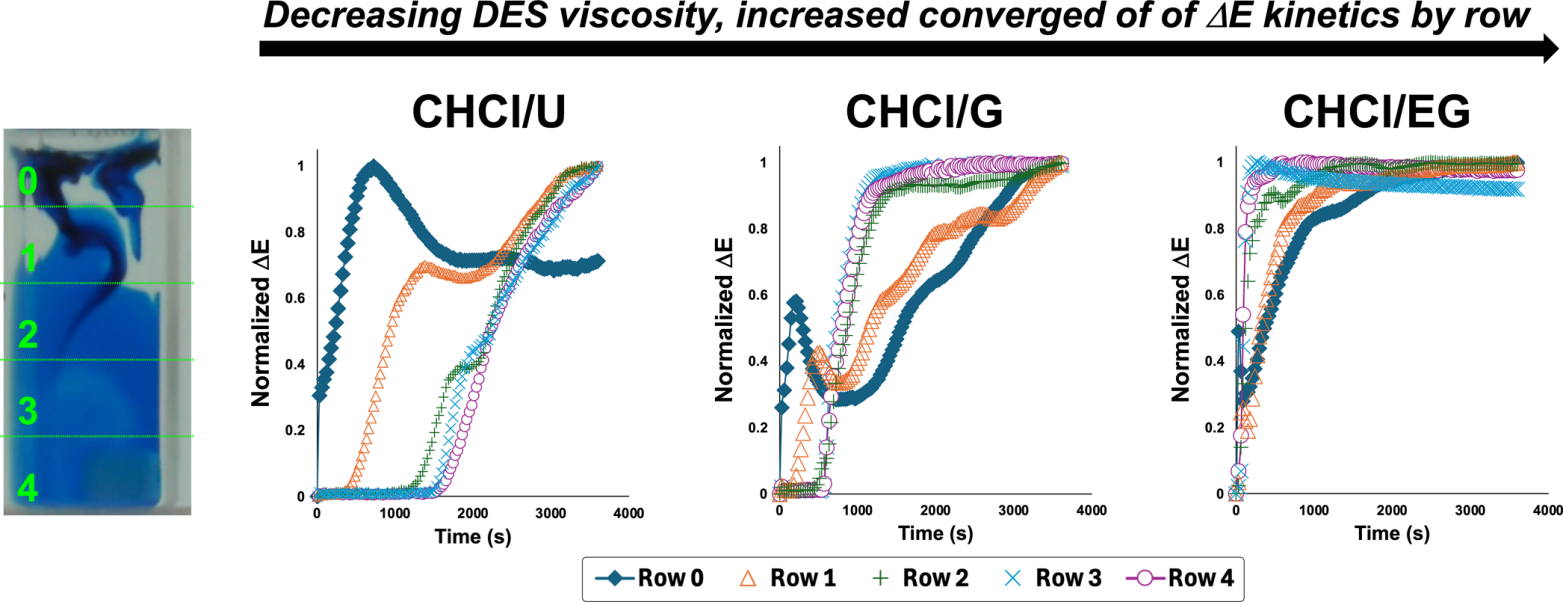
Spatially resolved mixing analysis in DESs using row-segmented Δ*E* measurements. Left: The cuvette is divided into five horizontal rows (0–4) to monitor concentration gradients during dye mixing. Right: Normalized Δ*E* profiles show distinct mixing kinetics across DES formulations of decreasing viscosity: ChCl/U (most viscous), ChCl/G (intermediate), and ChCl/EG (least viscous). Row 0 (top) consistently shows fastest color change, while lower rows exhibit delayed responses that converge more rapidly in less viscous systems.

The *Contact* metric from *Kineticolor* provided complementary insight into the evolution of concentration boundaries during mixing. At 25 °C, all DES formulations showed high initial *Contact* values that decreased slowly, with ChCl/U maintaining elevated *Contact* scores throughout the experiment. Temperature increase led to more rapid *Contact* decay, particularly evident in the ChCl/G system where 60 °C conditions showed a dramatic improvement in mixing uniformity compared to 25 °C.

### 2.6 Temperature–Viscosity Relationships

While plateau mixing times generally correlated with DES viscosity, notable exceptions emerged. At 60 °C, ChCl/G and ChCl/U achieved faster mixing than their viscosity values would predict, suggesting that temperature-dependent changes in DES microstructure may influence mixing beyond simple viscosity effects. This nonlinear relationship between viscosity and mixing time highlights the complexity of mass transport in hydrogen-bonded ionic systems.

The cuvette experiments established that

1. DES mixing times can exceed those of conventional solvents by 2–3 orders of magnitude,
2. Temperature dramatically improves mixing efficiency, with 60 °C providing optimal conditions for all DES tested in the range of temperatures tested, and
3. Computer vision metrics successfully capture both temporal and spatial aspects of mixing in these challenging systems.

These baseline measurements, highlights from which are summarized in Figure 11, provide essential context for understanding mixing effects in reactive DES systems discussed in subsequent sections.

**Figure 11:**
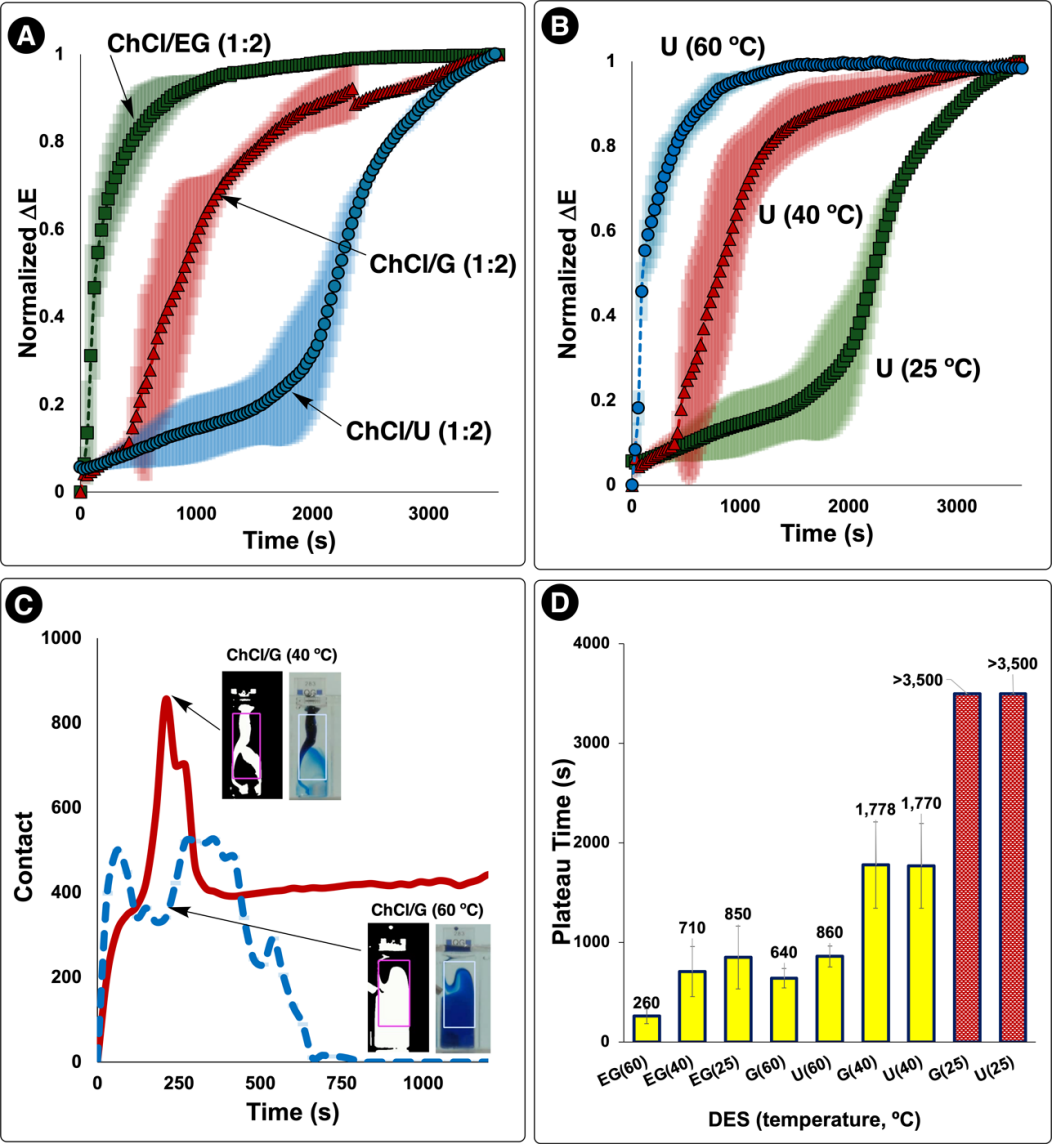
Temperature-dependent mixing dynamics in deep eutectic solvents monitored by computer vision. (A) Normalized Δ*E* profiles for three DES formulations (ChCl/EG, ChCl/G, ChCl/U) at 25 °C showing distinct mixing kinetics, with ChCl/U failing to reach complete homogenization (i.e., no plateau) within 1 h. (B) Effect of temperature on ChCl/U mixing dynamics, demonstrating accelerated mixing at elevated temperatures with complete mixing achieved at 60 °C. (C) *Contact* metric analysis revealing transient concentration boundaries during mixing, with inset images showing binary threshold detection of dye distribution. ChCl/G at 40 °C exhibits prolonged heterogeneity compared to the same system at 60 °C. Higher temperature leads to *Contact* returning to zero more quickly, signaling complete homogeneity. (D) Summary of plateau mixing times across all tested DES formulations and temperatures. Error bars represent standard deviations from triplicate experiments. Red bars indicate systems that did not reach homogenization within the observation period.

### 2.7 Dye Mixing Measurements with DESs: Schlenk Tube Studies

#### 2.7.1 The Impact of Vessel Geometry on DES Mixing

In early chemical methodology development, the typically small scale operations can lead vessel geometry to be overlooked in reaction optimization.^111^ However, while one might presume such parameters to be the remit of the chemical engineer rather than the chemist, mixing-critical factors like vessel geometry cannot be ignored, even during small scale discovery efforts. This point is further emphasized when viscous reaction media like DESs are employed.

To investigate how our computer vision methods might be applied to assessing the impact of vessel choice on DES mixing efficiency, we used two Schlenk tubes with varying outer diameters: narrow (17.5 mm) and wide (38.0 mm). Each tube was filled with 5 mL of DES and dye dispersion monitored via video recording. The same stirrer bar and stirring rate were used in all cases. The impact of vessel shape is shown in Figure 12, where it can be seen that the 5 mL of DES charged to fill Schlenk tube does so with a different overall liquid height. In the wider Schlenk tube, a more uniform mixture was obtained in a shorter period of time compared to the narrower Schlenk tube (Table 2). This effect was more pronounced for ChCl/G and ChCl/U versus the least viscous formulation, ChCl/EG. Indeed, even at 60 °C, the reduced viscosity of ChCl/G and ChCl/U was not enough to overcome mass transport restrictions in the narrow Schlenk tubes. To further domenstrate the exaggerated mixing challenges associated with the narrower vessel, switching to a smaller stirrer bar (6 mm × 3 mm rather than 12 mm × 5 mm) resulted in insufficient mixing and no plateau time that would represent an overall mixing time (see Supporting Information for full details).

**Figure 12:**
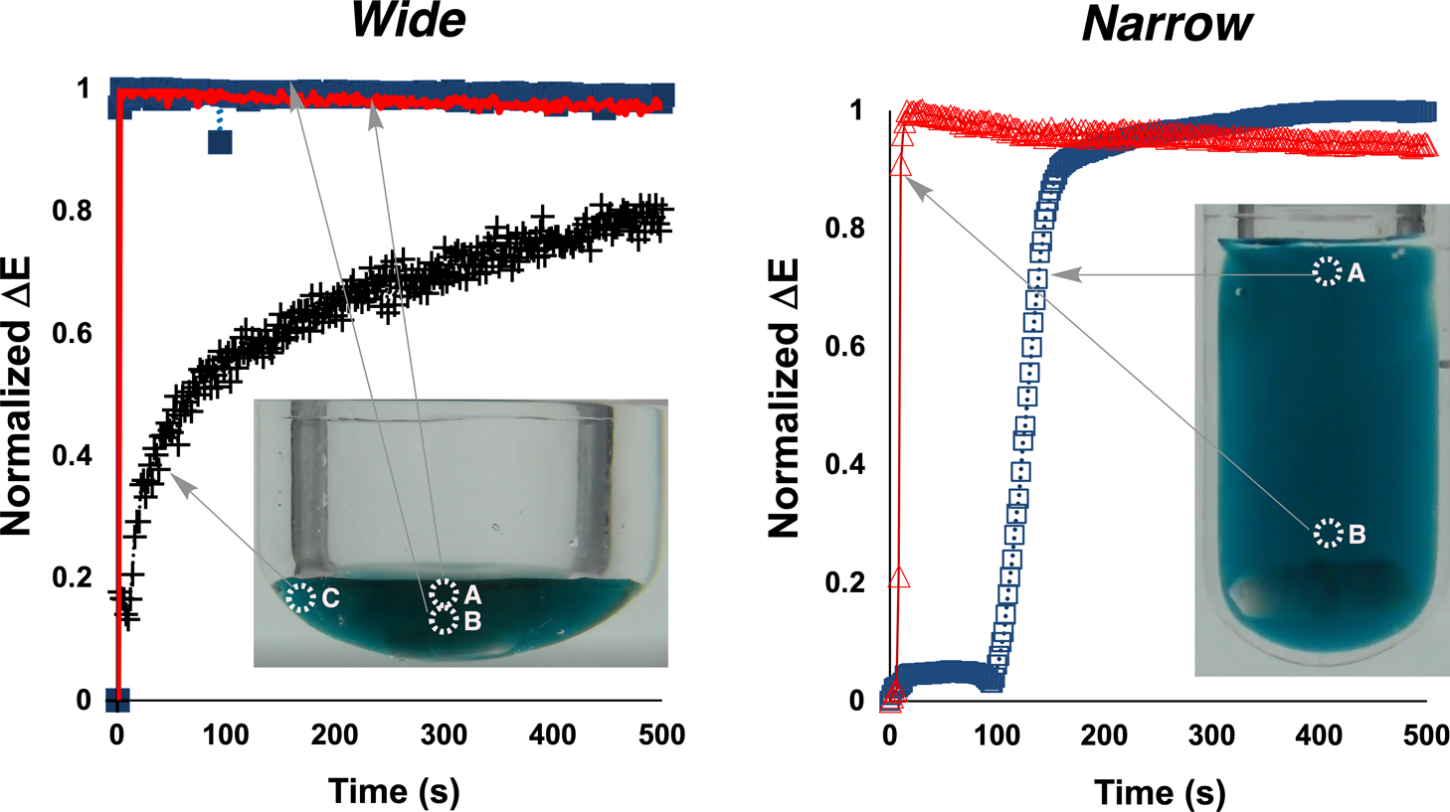
Comparison of mixing dynamics in narrow and wide vessel geometries for ChCl/U DES at 60 °C. Normalized Δ*E* profiles over time for three spatial regions in wide (left) and narrow (right) Schlenk tubes containing the same volume of choline chloride/urea DES. Left: In the wide vessel, rapid mixing is observed throughout all regions (blue squares, red triangles, black crosses). Right/Conversely, the narrow vessel shows significant spatial heterogeneity with delayed mixing in the lower region (blue squares). Inset images: The vessel geometries with sampling regions labeled. The dramatic difference in mixing profiles demonstrates the critical importance of vessel geometry selection when working with viscous deep eutectic solvents, even at elevated temperatures (60 °C). Stirring rate: 700 rpm Stirrer geometry: 12 mm × 5 mm.

**Table 2:** Impact of Schlenk Tube Geometry on Overall Mixing Time (s) in the DES Formulations at 25 and 60 °C

DES^a^	temperature (°C)	narrow	wide
ChCl/EG	25	690	29
ChCl/EG	60	270	25
ChCl/G	25		60
ChCl/G	60	660	19
ChCl/U	25		57
ChCl/U	60	630	27

a All DES components were used in a 1:2 ratio (with respect to moles) in the order they appear.

#### 2.7.2 DESs in Overhead Stirred Tank Systems

For industrial-scale operations, magnetic stirrers become impractical, necessitating overhead mixers with specialized impellers. To exemplify the use of the same computer vision workflow to analyze DES mixing on the larger scale, we employed an impeller with two 4-bladed 45° pitch-blade turbines impeller due to its ability to generate both axial and radial flow patterns, suitable for viscous media. This scaled-up example of monitoring mixing via video analysis also became an opportune case study through which to exemplify the use of spatially resolved mixing metrics based on texture analysis. Building on previous efforts,^111^ we employed gray-level co-occurrence matrix (GLCM) analysis to quantify mixing heterogeneity through texture properties. As summarized in Figure 13, GLCM analysis converts video frames into matrices based on the spatial relationships between pixel gray levels, enabling calculation of metrics such as entropy (which measures global unpredictability and reaches maximum values during random mixing states), angular second moment (ASM, which quantifies global pattern regularity and achieves minimum values when no discernible pattern exists), and homogeneity (which captures local neighbor similarity and decreases when adjacent pixels differ significantly). These texture-based metrics complement averaged color analysis by providing spatially sensitive detection of mixing phenomena that may not be captured through simple pixel averaging across the entire reactor cross-section. For this scale-up study, we focused on monitoring differences in the ChCl/U formulation at 25 and 60 °C. At 25 °C, significant heterogeneity persists even after extended mixing time, while at 60 °C, complete homogenization is achieved (Figure 14).

**Figure 13:**
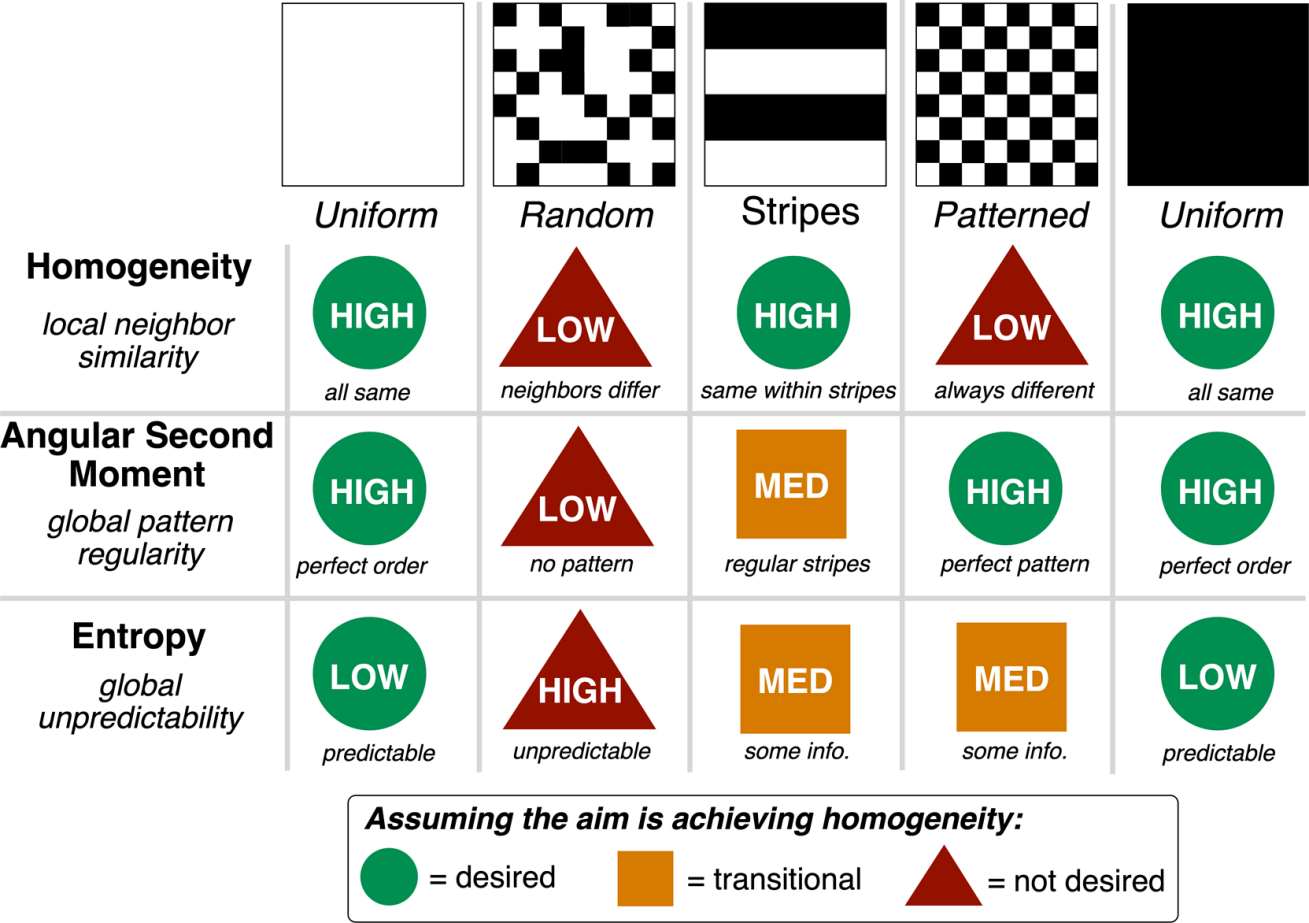
Illustration of how three key GLCM (gray level co-occurrence matrix) metrics respond to different visual patterns. The *Homogeneity* metric measures local pixel similarity, *Angular Second Moment* detects global pattern regularity, and *Entropy* quantifies randomness. These metrics enable automated assessment of mixing quality by distinguishing between uniform (well-mixed) and heterogeneous (poorly mixed) regions in reactor images.

**Figure 14:**
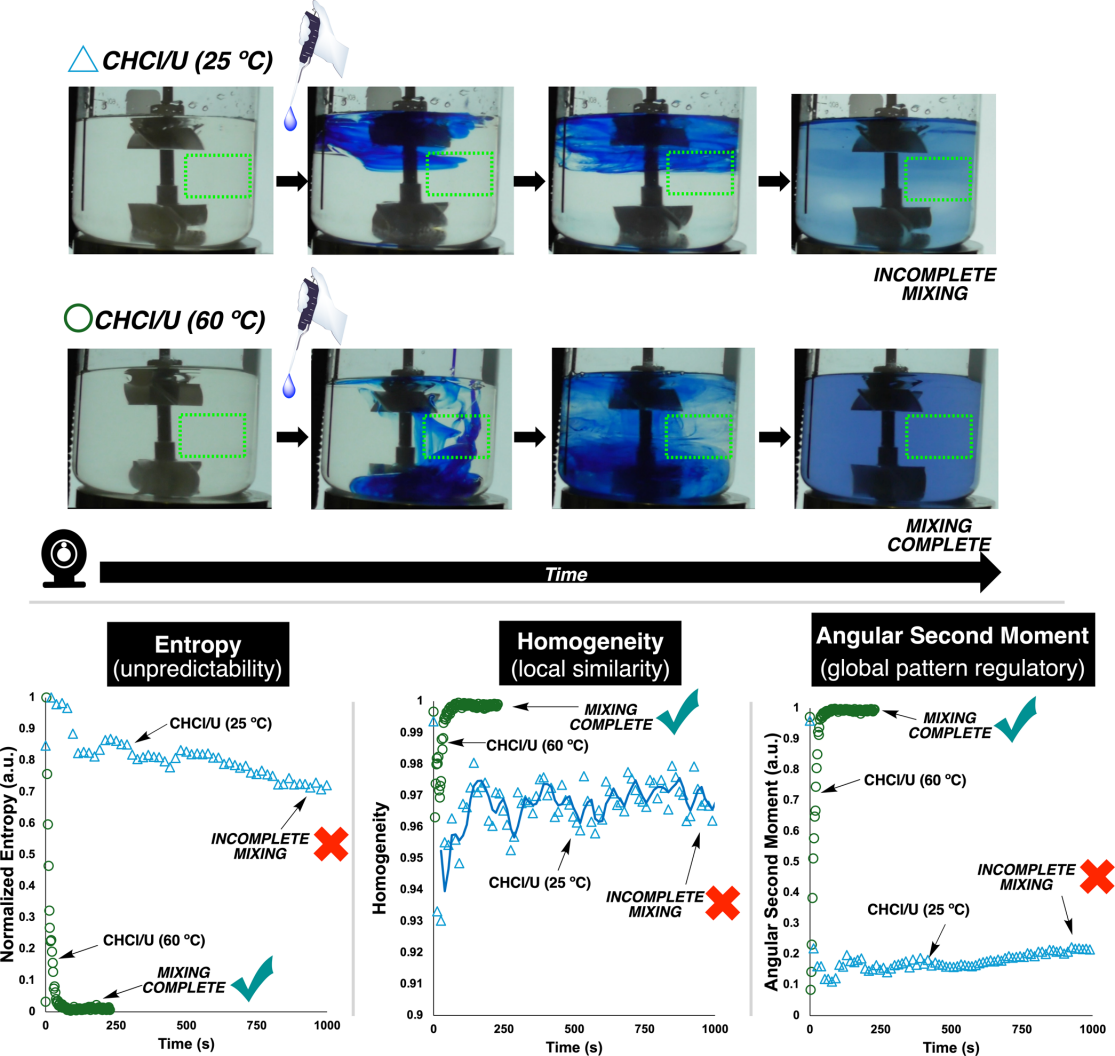
Computer vision analysis of mixing behavior in DESs at different temperatures in an overhead stirred vessel. Top: visual progression of mixing for ChCl/U system at 25 and 60 °C, with blue dye addition demonstrating faster mixing at elevated temperature. Green dashed boxes highlight regions of interest analyzed. Bottom: quantitative and spatially sensitive texture analysis metrics over time: Entropy measures unpredictability/disorder, Homogeneity tracks local similarity, and Angular Second Moment captures global pattern regularity. Higher temperature (60 °C; open green circles) accelerates mixing completion as indicated by metric stabilization, while lower temperature (25 °C; open blue triangles) shows prolonged mixing dynamics.

#### 2.7.3 Reaction Case Study: Borohydride-Mediated Aldehyde Reduction

Having established the use of computer vision as a means of quantitatively assessing the factors impacting mixing in DESs, we next aimed to investigate an example of where such knowledge could support the development of synthetic methodologies employing DESs.

Work by Azizi et al. detailed a green route for reduction of carbonyl compounds in choline chloride/urea DES as an alternative to alcoholic solvents, using sodium borohydride as the reducing agent.^85^ Guided by our computer vision-enabled measurements of mixing times, we investigated the impact of vessel geometry, DES formulation, and temperature on the reduction of 5-chloro-2-fluorobenzaldehyde. As shown in Table 3, mixing limitations dramatically affected product conversion. Focusing on the most viscous ChCl/U DES, the effect of vessel geometry on mixing was most pronounced, with only 17.8% conversion in the narrow vessel compared to 52.1% in the wider vessel. When the temperature was elevated to 60 °C for ChCl/U, thereby reducing viscosity and improving mixing as demonstrated in our earlier analyses, conversion increased dramatically to 87.8%. These results directly validate our computer vision approach to mixing analysis in DES systems and demonstrate how proper consideration of both vessel geometry and temperature can overcome mass transport limitations in highly viscous media.

**Table 3:**
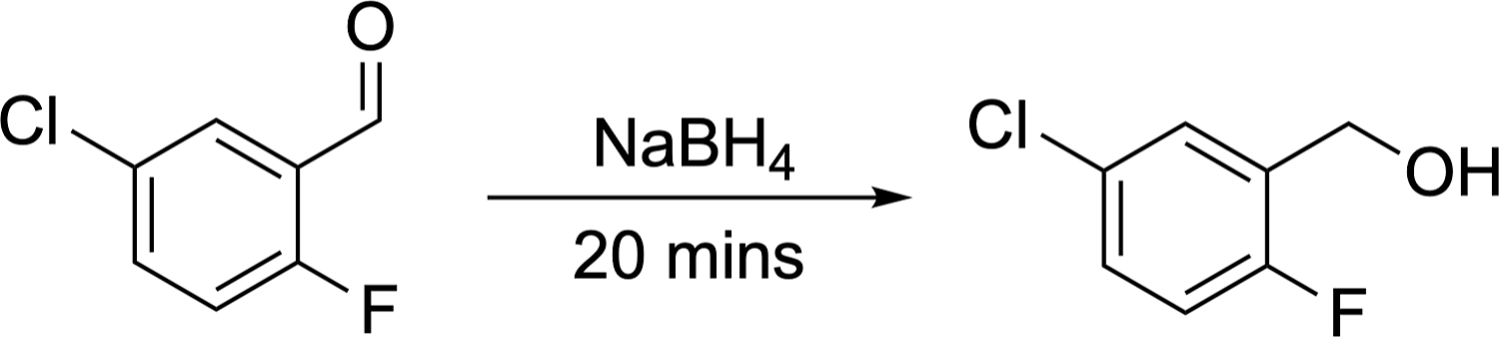
Effect of Vessel Geometry, DES Formulation, and Temperature on Sodium Borohydride-mediated Reduction of 5-Chloro-2-fluorobenzaldehyde^a^

solvent	vessel	temp.(°C)	conversion (%)
ChCl/U	narrow	40	17.8 ± 11.7
ChCl/U	wide	40	52.1 ± 14.0
ChCl/U	wide	60	87.8 ± 10.0

a Reactions were conducted using 5 mmol of aldehyde, 10 mmol NaBH_4_, and 5 mL solvent for 20 min. Narrow vessel = 17.5 mm outer diameter Schlenk tube; wide vessel = 38.0 mm outer diameter Schlenk tube. Conversion determined by ^19^F NMR using α, α, α–trifluorotoluene as internal standard. Stirring rate: 700 rpm Stirrer geometry: 12 mm × 5 mm.

#### 2.7.4 Computational Fluid Dynamics

To complement the experimental observations, computational fluid dynamics (CFD) simulations were performed using the *k*-omega SST turbulence model to investigate mixing dynamics across different DES formulations, temperatures, and vessel geometries, consistent with those tested experimentally. CFD analysis confirmed the experimentally observed impact of vessel geometry on mixing effectiveness when using DESs.

The velocity contour plots revealed striking differences in mixing efficiency based on both DES composition and vessel geometry (Figure 15). In methanol, the stirrer bar’s influence extended throughout the entire vessel volume regardless of geometry. However, as DES viscosity increased, the mixing zone progressively diminished, with the effect most pronounced in narrow vessel configurations. Highly viscous formulations (ChCl/G at 20 °C and ChCl/U at 20 °C) showed severely restricted mixing zones confined to the immediate vicinity of the stirrer bar.

**Figure 15:**
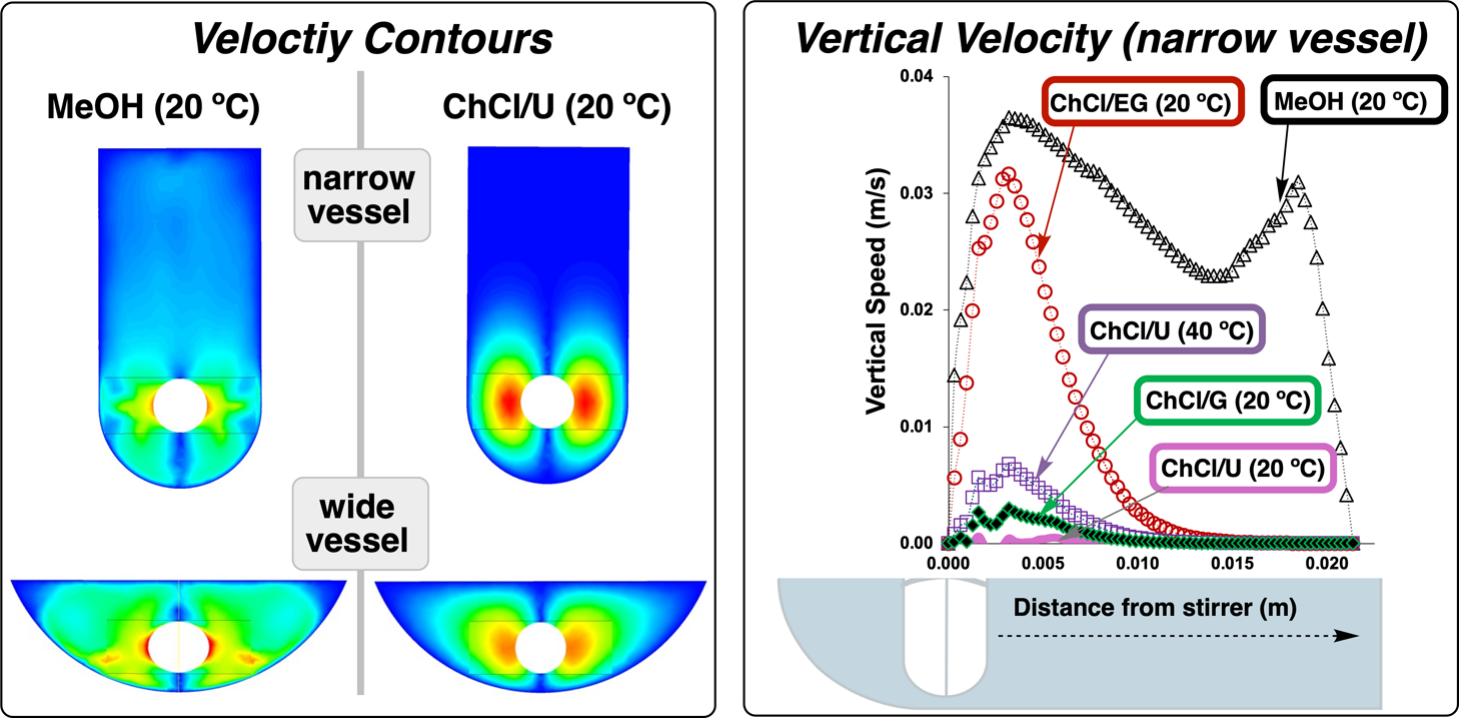
Left: velocity contour plots comparing methanol and ChCl/U DES at 20 °C in narrow and wide vessels, showing how the highly viscous DES exhibits severely restricted mixing zones confined near the stirrer bar in narrow vessels. Right: quantitative analysis of vertical velocity decay as a function of distance from the stirrer bar in narrow vessel for different DES formulations and temperatures. Methanol maintains significant flow throughout the vessel while ChCl/U and ChCl/G at 20 °C shows nearly zero velocity beyond the immediate stirrer zone. Simulations were conducted at 700 rpm for 60 s (0.05 s time steps) for all DES formulations, while methanol simulations ran for 10 s (0.001 s time steps).

Temperature elevation dramatically improved mixing performance in all DES formulations. For ChCl/U, increasing temperature from 20 to 60 °C reduced viscosity from 1372 cP to 68.6 cP, resulting in substantially enhanced velocity fields and more turbulent flow patterns. These computational predictions aligned well with our experimental observations of reduced mixing times at elevated temperatures.

The CFD analysis confirmed several critical findings:

1. Wide vessel geometries significantly outperform narrow vessels for mixing viscous DES formulations.
2. Temperature elevation effectively improves DES mixing, particularly for highly viscous formulations.
3. The transition from turbulent to laminar flow occurs progressively as DES viscosity increases.
4. Stirrer bar effectiveness is severely limited in cases where the solvent height-to-volume ratio is high (i.e., in moving from using a vessel with a wide to a narrow diameter while maintaining the same solvent volume).

Additional CFD analyses, including streamline visualizations and component velocity breakdowns, are available in the Supporting Information. These simulations offer guidance for reactor design when using DES solvents in sustainable chemistry applications, and demonstrate the synergistic value of combining CFD with experimental computer vision-enabled mixing analysis.

These simulations provide a predictive framework for optimizing reaction conditions when using DES solvents, supporting the experimental findings and offering guidance for reactor design in sustainable chemistry applications.

#### 2.7.5 Limitations

This study focused primarily on developing and validating computer vision methodologies for monitoring mixing in viscous DES systems rather than providing comprehensive mixing characterization across the full spectrum of available DES formulations. Our investigation was limited to three representative choline chloride-based DES formulations (ChCl/EG, ChCl/G, ChCl/U) at selected temperature ranges and vessel geometries. The computer vision analysis captures primarily surface-visible phenomena within the reactor, with no explicit penetration into fluid regions not visible through reactor walls. Additionally, our synthetic validation was demonstrated through a single reaction system (sodium borohydride reduction), and the generalizability to other reaction types requires further investigation. The primary objective was to establish computer vision as a viable analytical tool for DES mixing analysis rather than to create an exhaustive database of mixing parameters across all possible DES compositions and operating conditions.

## 3 Conclusions

This work establishes computer vision as a powerful, quantitative approach for analyzing mixing challenges in highly viscous deep eutectic solvents across multiple scales and conditions. Using the Kineticolor software platform, we have systematically quantified the dramatic impact of DES formulation, temperature, and vessel geometry on mixing dynamics, revealing mixing time variations spanning 4 orders of magnitude—from seconds in methanol to over 60 min in viscous ChCl/U at room temperature.

Our comprehensive analysis across three representative DES formulations (ChCl/EG, ChCl/G, ChCl/U) demonstrates that temperature elevation from 25 to 60 °C can reduce mixing times by up to 10-fold, while vessel geometry optimization provides additional significant improvements. The spatially resolved Δ*E* measurements proved particularly valuable for revealing persistent concentration gradients and vertical stratification in viscous media—phenomena that would be invisible to traditional single-point probe measurements. Among the evaluated computer vision metrics, the Contact parameter effectively captured transient mixing boundaries, while texture-based metrics (Homogeneity, Entropy, Angular Second Moment) provided complementary insights into global mixing uniformity.

The integration of computer vision analytics with CFD modeling represents a particularly valuable contribution for understanding mixing phenomena in viscous DES systems. Our approach provides noninvasive, spatially and temporally resolved experimental validation of CFD predictions without disturbing flow fields—a critical advantage over traditional probe-based methods that can alter flow patterns in viscous systems. The validated computational framework enables prediction of mixing behavior across different operating conditions, supporting process optimization decisions without extensive trial-and-error experimentation.

The practical implications of these findings were demonstrated through sodium borohydride-mediated aldehyde reduction, where poor mixing conditions (narrow vessel, 40 °C, ChCl/U) resulted in only 11.8% yield, while optimized mixing conditions (wide vessel) achieved higher yields of 36.4 and 79.4% at 40 and 60 °C, respectively. This significant improvement directly validates the utility of our computer vision approach for informing synthetic methodology development in DES systems.

This work addresses a critical gap in sustainable chemistry implementation by providing accessible tools for quantifying and optimizing mixing in viscous green solvents. The computer vision approach requires only standard laboratory equipment (any camera) and provides insights that would otherwise require expensive specialized instrumentation. By enabling rational optimization of reaction conditions based on quantitative mixing analysis, this methodology bridges the gap between sustainability goals and practical synthetic implementation, facilitating broader adoption of DES solvents in chemical synthesis and process applications.

Future work should extend this approach to additional DES formulations, explore the effects of additives and cosolvents on mixing dynamics, and investigate applications in continuous flow systems where mixing optimization is equally critical for process intensification.

## 4 Experimental Section

### 4.1 Materials

Deep eutectic solvents were prepared from choline chloride (ChCl, Sigma-Aldrich, ≥98%), ethylene glycol (EG, ≥99%), glycerol (G, ≥99%), and urea (U, ≥98%) in 1:2 molar ratios. All components were dried under vacuum at 60 °C for 24 h before use. DESs were prepared by heating and stirring component mixtures at 100 °C until homogeneous, transparent liquids were obtained (∼200 mL scale). Dyed DES stock solutions were prepared at 5 mg/mL using indigo carmine dye.

### 4.2 Viscosity Measurements

Kinematic viscosities were measured using Ostwald viscometers at temperatures ranging from 20 to 60 °C. DES samples were loaded to level A, drawn to level B using a pipet filler, and flow times from B to C were recorded in quintuplicate. Viscosities were calculated using the equation η_1_ = (ρ_1_ *t*_1_/ρ_2_ *t*_2_) × η_2_, where known literature values served as references.

### 4.3 Computer Vision Mixing Analysis

Video recordings were captured using a Panasonic HC-W580 camera positioned in a Godox 45 cm^3^ LED studio tent with HSK A4 LED backlighting. For cuvette studies, 2.9 mL clear DES was combined with 0.1 mL dyed DES in Hellma quartz cuvettes (10 mm path length) with magnetic stirring (3 × 6 mm stirrer bar, 500 rpm). For Schlenk tube studies, 4.8 mL clear DES was mixed with 0.2 mL dyed DES using 12 × 5 mm stirrer bars at 700 rpm in vessels of varying diameter (narrow: 17.5 mm; wide: 38.0 mm). All mixing experiments were conducted at controlled temperatures (25, 40, 60 °C) with video recording for 1 h. Analysis was performed using Kineticolor software, calculating Δ*E* color difference (eq 2), *Contact* boundary parameters, and texture-based metrics including Gray Level Co-occurrence Matrix (GLCM) analysis for spatial heterogeneity quantification.

### 4.4 Synthetic Validation Studies

Sodium borohydride reduction of 5-chloro-2-fluorobenzaldehyde (0.792 g, 5 mmol) was performed in ChCl/U DES (5 mL) using NaBH_4_ (0.379 g, 10 mmol) with stirring (12 × 5 mm stirrer bar, 700 rpm) for 20 min at specified temperatures. Reactions were quenched with 0.1 M HCl (50 mL) and extracted with diethyl ether (3 × 20 mL). Conversions were determined by ^19^F NMR using α, α, α-trifluorotoluene as internal standard.

### 4.5 Computational Fluid Dynamics

CFD simulations were performed using ANSYS Fluent R17.1 with *k*-omega SST turbulence model. Geometries were constructed based on measured vessel dimensions (narrow: 7.55 mm internal radius; wide: 17.6 mm internal radius) with 5 mL fluid volumes. Simulations used experimental viscosity and density data, with 700 rpm stirrer rotation, running for 60 s at 0.05 s time steps for DES systems.

## Supplementary Material



## Data Availability

All data are included in an machine-readable format at: https://doi.org/10.6084/m9.figshare.29279894.v2.

## Notes

Experimental procedures, reactor setup, CFD calculations, and computer vision methods are further detailed in the Supporting Information (PDF)
