# Supplementary material for: Computer Vision Helps Experimentally Monitor Mixing Effects in Deep Eutectic Solvents
Source: ACS Sustain Chem Eng. 2025 Oct 3;13(41):17241–56. doi: 10.1021/acssuschemeng.5c05783 (PMC12541803; doi:10.1021/acssuschemeng.5c05783)
Supplement: Supplementary file 1 [file sc5c05783_si_001.pdf]

Computer Vision Helps Experimentally Monitor Mixing Effects in Deep Eutectic Solvents

Calum Fyfe, Rhoda Duncan, Timothy J.D. McCabe, Kristin Donnachie, Henry Barrington, and Marc Reid*

Department of Pure and Applied Chemistry, University of Strathclyde, Glasgow G1 1XL, U.K.

Supporting Information

Table of Contents

Experimental Details 3

DES Synthesis 3

Type I ChCl/$ZnCl_2$ 3

Type III ChCl/EG 3

Type III ChCl/G 3

Type III ChCl/U 3

Type I $ZnCl_2$ 3

Dyed DES 3

Viscosity measurements 4

Proof-of-concept – tracking mixing effects in viscous media 5

pH titrations with phenolphthalein in water and in a water/glycol mixed solvent system (Figure 6 – 8) 5

Non-reactive mixing 5

Dye mixing measurements with DESs: cuvette studies (Table 1, Figures 10 – 11) 5

Dye mixing measurements with DESs: Schlenk tube studies (Figure 12, Table 2) 6

Reaction case study: Borohydride-mediated aldehyde reduction (Table 3) 7

NMR Spectra 9

Computational Details 31

Computational Fluid Dynamics Modelling with Ansys R17.1 31

Geometry 31

Meshing 33

Setup 33

Plateau Analysis 34

Additional Visualizations 35
Proof-of-concept – tracking mixing effects in viscous media 35
Dye mixing measurements with DESs: cuvette studies 38
Computational Fluid Dynamics 39

NOTE – in addition to the higher-level details shared in this document, a zipped folder of machine-readable data, ordered according to the Figure and Table numbers in the main text, is available as part of the supporting information via figshare:

https://doi.org/10.6084/m9.figshare.29279894.v2

For information on licensing Kineticolor software, please contact the corresponding author and the University of Strathclyde technology transfer office:

marc.reid.100@strath.ac.uk ; iprmanager@strath.ac.uk

Experimental Details

DES Synthesis

The following methods were used to produce the DESs, on 1 mol scale approximately 200 mL of DES was produced:

Type I ChCl/$ZnCl_2$

Choline chloride (69.812 g, 0.5 mol) was mixed with $ZnCl_2$ (136.286 g, 1 mol) with stirring at 100 °C until a clear homogenous mixture was formed.

Type III ChCl/EG

Choline chloride (69.812 g, 0.5 mol) was mixed with ethylene glycol (62.068 g, 1 mol) with stirring at 100 °C until a clear homogenous mixture was formed.

Type III ChCl/G

Choline chloride (69.812 g, 0.5 mol) was mixed with glycerol (92.094 g, 1 mol) with stirring at 100 °C until a clear homogenous mixture was formed.

Type III ChCl/U

Choline chloride (69.812 g, 0.5 mol) was mixed with urea (60.055 g, 1 mol) with stirring at 100 °C until a clear homogenous mixture was formed.

Type IV $ZnCl_2$/U

$ZnCl_2$ (68.143 g, 0.5 mol) was mixed with Urea (105.096 g, 1.75 mol) with stirring at 100 °C until a clear homogenous mixture was formed.

Dyed DES

Dyed DES stock solutions were prepared at 5 mg /mL for all three DES formulations. 0.3 g of indigo carmine dye was added to 60 mL of DES and mixed until dissolved or fully dispersed.

Viscosity measurements

An Ostwald viscometer was placed in a water bath at 20 °C and filled with DES up to level A (Figure S1). A pipette filler was attached to the narrow arm; the liquid sucked to above level B. The pipette filler was removed, and the time it took for the liquid to drop from B to C was recorded. This process was repeated five times.

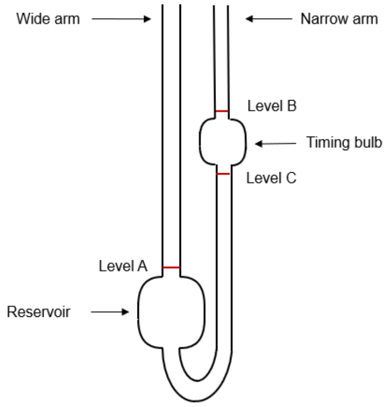

Figure S1: Ostwald's viscometer

This was repeated for all three DESs at several temperatures, as can be seen in Table S1 Due to the DESs varying in viscosity, a smaller viscometer was used for the ChCl/EG.

Table S1: Viscometer repeat conditions.

**DES**	**Viscometer**	**Run 1**	**Run 2**	**Run 3**	**Run 4**
ChCl/EG	1	20 °C	25 °C	40 °C	60 °C
ChCl/G	2	20 °C	25 °C	40 °C	60 °C
ChCl/U	2	20 °C	40 °C	60 °C	N/A

The following equation was used to calculate the viscosity of the DESs at a specific temperature where the viscosity was known for one DES at a particular temperature.,

$$\eta_1 = \frac{\rho_1 t_1}{\rho_2 t_2} \times \eta_2$$

Where,

η_1= Viscosity of component 1 at temperature T_1 (cP)

ρ_1= Density of component 1 (g cm^{-3})

t_1= Time for liquid 1 to travel from B to C (s)

η_2 = Viscosity of Component 2 at temperature T_2 (cP)

ρ_2 = Density of component 2 (g cm^{-3})

t_2 = Time for liquid 2 to travel from B to C (s)

The following data was used as the basis of calculations:

Table S2: DES physical property obtained from https://doi.org/10.1021/acs.chemrev.0c00385

DES	Ratio	Temperature (°C)	Density (g cm^{-3})	Viscosity (cP)
ChCl/EG	1:2	25	1.12	37
ChCl/G	1:2	25	1.18	376
ChCl/U	1:2	-	1.25	-

Proof-of-concept – tracking mixing effects in viscous media

pH titrations with phenolphthalein in water and in a water/glycol mixed solvent system (Figure 6 – 8)

From earlier interactions with our industrial collaborator at Fujifilm, Grangemouth (Scotland) in relation to our first computer vision-focused mixing paper (https://doi.org/10.1021/acs.oprd.2c00216), access was granted to archive video footage used by Fujifilm to educate staff on mixing effects on chemical process scale-up. The videos used for the proof-of-concept study were used as supplied. The videos should an acidic solution of phenolphthalein in a 3 L beaker, being basified by addition of aqueous sodium hydroxide from a measuring cylinder to the surface of the reaction mixing. Overhead stirring was provided by bespoke glass impellers made by Scott Glass Ltd (Stirling, Scotland; https://scottglass.co.uk/). Stirring rate was held at 400 RPM. Further details of the volumes of each component used were not available.

Non-reactive mixing

Dye mixing measurements with DESs: cuvette studies (Table 1, Figures 10 – 11)

A hotplate with a water bath was set up in a Godox 45 cm^3 Triple LED studio light tent with an HSK A4 LED light box light pad behind. A Panasonic HC-W580 camera was set up on a tripod in front of the light box. In another water bath, the stock DESs and dyed DESs were prewarmed to the desired temperature (25, 40, or 60 °C). For all temperature and DES combinations, 2.9 mL of

fresh DES was transferred to a Hellma quartz glass 10 mm cuvette via a 5 mL syringe and placed into the water bath set to the desired temperature. A 3 mm diameter 6 mm length cylindrical stir bar was added to the cuvette, and mixing commenced at 500 rpm. The cuvette and camera positions were aligned, ~70 cm from the lens to the cuvette, 8X zoom was used, auto white balance, 1/50 shutter speed, and manual focus. Once positioned, the Cuvette was left to mix for at least 1 min, allowing the system to settle to steady-state mixing. Then, recording was commenced, 0.1 mL of the appropriate dyed DES was added to the cuvette via a 1 mL syringe, and the mixing process was recorded for 1 hour. This capture process was repeated for all combinations of DES and Temperature, and all repeated in triplicate.

Dye mixing measurements with DESs: Schlenk tube studies (Figure 12, Table 2)

A hotplate with a water bath was set up in a Godox 45 cm^3 Triple LED studio light tent with an HSK A4 LED light box light pad behind. A Panasonic HC-W580 camera was set up on a tripod in front of the light box. In another water bath, the stock DESs and dyed DESs were prewarmed to the desired temperature (25, 40, or 60 °C). For all temperature and DES combinations, 4.8 mL of fresh DES was transferred to the Schlenk tube via a 5 mL syringe and placed into the water bath set to the desired temperature. A 5 mm diameter 12 mm length cylindrical stir bar with a pivot was added to the Schlenk (additional experiments were also conducted using a 3 mm diameter 6 mm length cylindrical stir bar in the narrow Schlenk to investigate the impact of the stir bar selected), and mixing commenced at 700 rpm. The Schlenk and camera positions were aligned, ~30 cm from the lens to the cuvette, x7 zoom was used, auto white balance, 1/50 shutter speed, and manual focus. Once positioned, the Schlenk was left to mix for at least 1 min, allowing the system to settle to steady-state mixing. Then, recording was commenced, 0.2 mL of the appropriate dyed DES was added to the Schlenk via a 1 mL syringe, and the mixing process was recorded for 1 hour. This capture process was repeated for all combinations of DES, Temperature, and Schlenk tube size (narrow 17.5 mm diameter and wide 38 mm diameter).

DESs in overhead stirred tank systems (Figure 14)

In a 2L beaker, 1L of the choline chloride/urea DES was added, heated to 25/60 °C, and stirred using an overhead stirrer fitted with a double impeller set to 200 RPM. Once the DES had reached the desired temperature, the recording was started, and 10 mL of blue food coloring was added to the beaker via a measuring cylinder. The reaction was filmed using the Panasonic camcorder at 780p, 2X zoom for 4 minutes.

Reaction case study: Borohydride-mediated aldehyde reduction (Table 3)

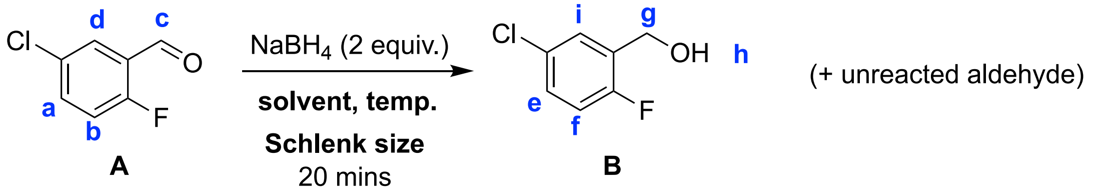

Figure S2: Reduction 5-chloro-2-fluorobenzaldehyde with F and H environment assignment

5-chloro-2-fluorobenzaldehyde, **A** (0.792 g, 5 mmol) was combined in 5 mL of the ChCl/Urea DESs in the chosen diameter Schlenk tube (wide or narrow) and stirred (using a 12 x 5 mm stir bar) at 700 RPM for at least 1 minute at the desired temperature (40 or 60 ^{o}C). $NaBH_4$ (0.379 g, 10 mmol, 2 equiv.) was added and allowed to react for 20 minutes. The reaction was quenched with 50 mL 0.1 M HCl and extracted with diethyl ether (3 x 20 mL). The combined organic extracts were washed with saturated aqueous sodium chloride (2 x 20 mL). The mixture was then dried over magnesium sulfate and the solvent was removed in vacuo.

The commercially sourced starting material and extracted reaction mixtures (containing both product **B** and unreacted **A**) were analysed using ^{1}H and ^{19}F NMR. α, α, α–trifluorotoluene as an ^{19}F internal standard, which was prepared in a 10 mL volumetric flask at 20 mg/mL. NMR samples were prepared for the resultant reaction mixtures by dissolving 10-15 mg of material in 0.5 mL $CDCl_3$ and adding 0.5 mL of the internal standard solution.

Conversion was calculated from ^{19}F NMR and the following equation:

$$\% \, Assay = \frac{m_{IS}}{m_A} \cdot \frac{I_A}{I_{IS}} \cdot \frac{M_A}{M_{IS}} \cdot \frac{N_{19_{F},IS}}{N_{19_{F},A}}$$

Where:

$$m_i = mass\ of\ analyte\ (A)\ or\ internal\ standard\ (IS)$$

$$I_i = integration\ of\ selected\ peaks$$

$$M_i = molar\ mass$$

$$N_{19_{F},i} = number\ of\ F\ represented\ by\ the\ selected\ peaks$$

NMR Analysis of isolated starting material (5-Chloro-2-fluorobenzaldehyde; A):

^{1}H NMR ($CDCl_3$, 500MHz): δ 10.33 (1H, s, H_c), 7.86 (1H, dd, $^4J_{HH}$= 2 Hz, $^4J_{HF}$=6 Hz, H_d), 7.58 (1H, ddd, $^3J_{HH}$=8Hz, $^4J_{HH}$=2Hz, $^4J_{HF}$=3Hz, H_a), 7.18 (1H, t, `$^3J_{HH/F}$=8 Hz, H_b)

^{19}F NMR ($CDCl_3$, 500MHz): -124.57 (F_1)

Analysis of reduction reaction mixture:

^{1}H NMR ($CDCl_3$, 500MHz): δ 10.22 (1H, s, H_c), 7.75 (1H, dd, $^4J_{HH}$= 2 Hz, $^4J_{HF}$=6 Hz, H_d), 7.48, (1H, ddd, $^3J_{HH}$=8Hz, $^4J_{HH}$=2Hz, $^4J_{HF}$=3Hz, H_a), 7.36 (1H, dd, $^4J_{HH}$= 2 Hz, $^4J_{HF}$=6 Hz, H_i), 7.14 (1H, ddd, $^3J_{HH}$=8Hz, $^4J_{HH}$=2Hz, $^4J_{HF}$=3Hz, H_e), 7.08 (1H, t, $^3J_{HH/F}$=8 Hz, H_b), 6.91 (1H, t, $^3J_{HH/F}$=8 Hz, H_f), 4.66 (2H, s, H_g), 1.84 (1H, s, H_h).

^{19}F NMR ($CDCl_3$, 500MHz): -122.48 (product alcohol), -124.48 (aldehyde)

The spectra showed starting material peak at 124.57 ppm and a new peak seen at 122.48 ppm. The peak at 122.48 ppm is determined to be the desired product, this is confirmed by the ^{1}H NMR spectra.

Table S3: Estimated conversion from ^{19}F NMR, $m_{IS} = 10.02\ mg, I_{IS} = 3, M_{IS} = 146.11 \frac{g}{mol}, N_{19_{F,IS}} = 3, and\ N_{19_{F,A}} = 1$. *Relates to Table 3 in the manuscript.*

Solvent	Vessel	Temp (ºC)	Mass in NMR sample (m_A) mg	Area of starting material ^{19}F peak ($I_A, M_A = 158.56$)	Area of product ^{19}F peak ($I_A, M_A = 160.58$)	Conversion by ^{19}F NMR (%)
ChCl/Urea	Narrow	40	11.96	0.5609	0.0603	9.71
ChCl/Urea	Narrow	40	11.44	0.5862	0.2664	31.25
ChCl/Urea	Narrow	40	12.79	0.8748	0.1239	12.41
ChCl/Urea	Wide	40	9.98	0.3053	0.4162	57.69
ChCl/Urea	Wide	40	11.41	0.2747	0.4579	62.50
ChCl/Urea	Wide	40	10.15	0.4395	0.2496	36.22
ChCl/Urea	Wide	60	14.15	0.1423	0.8198	85.21
ChCl/Urea	Wide	60	14.47	0.0113	0.9075	98.77
ChCl/Urea	Wide	60	12.68	0.2111	0.8111	79.35

NMR Spectra

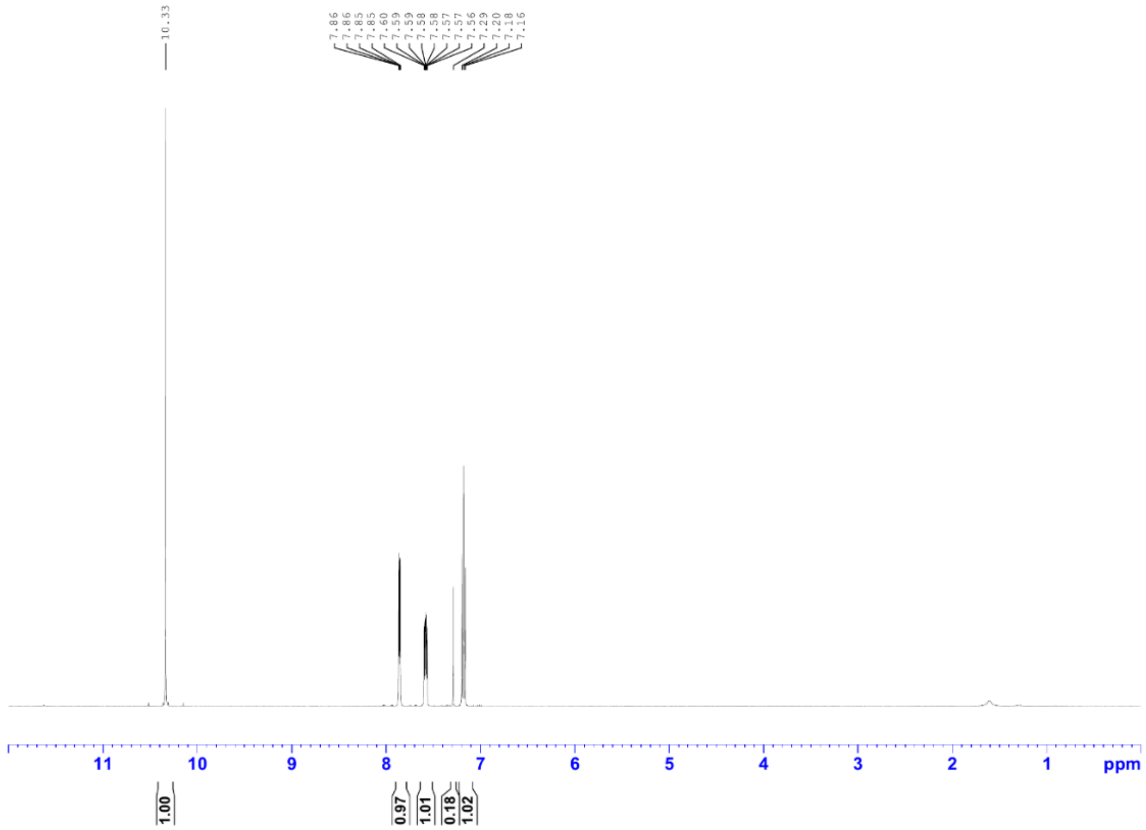

Figure S3: ^{1}H NMR of starting material A (5-chloro-2-fluorobenzaldehyde)

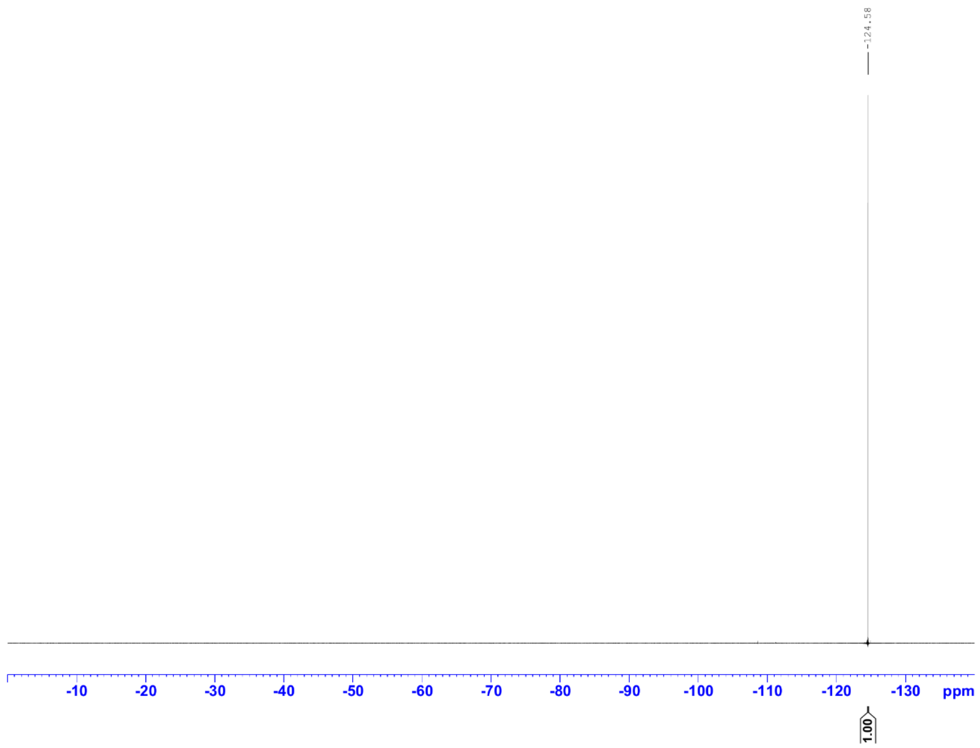

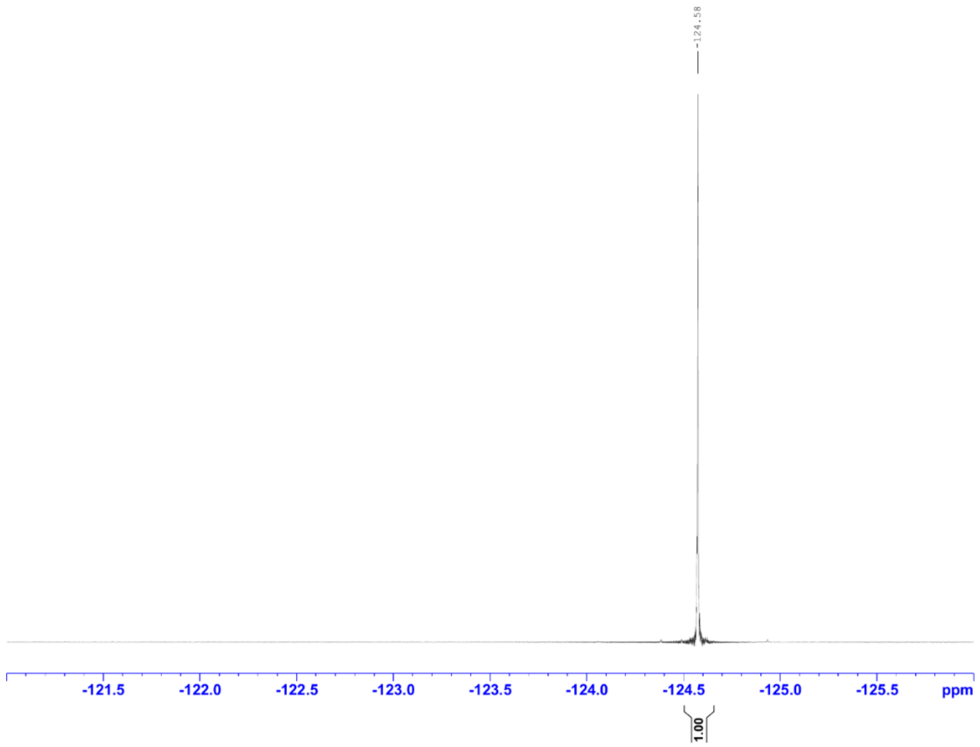

Figure S4: ^{19}F NMR of starting material A (5-chloro-2-fluorobenzaldehyde)

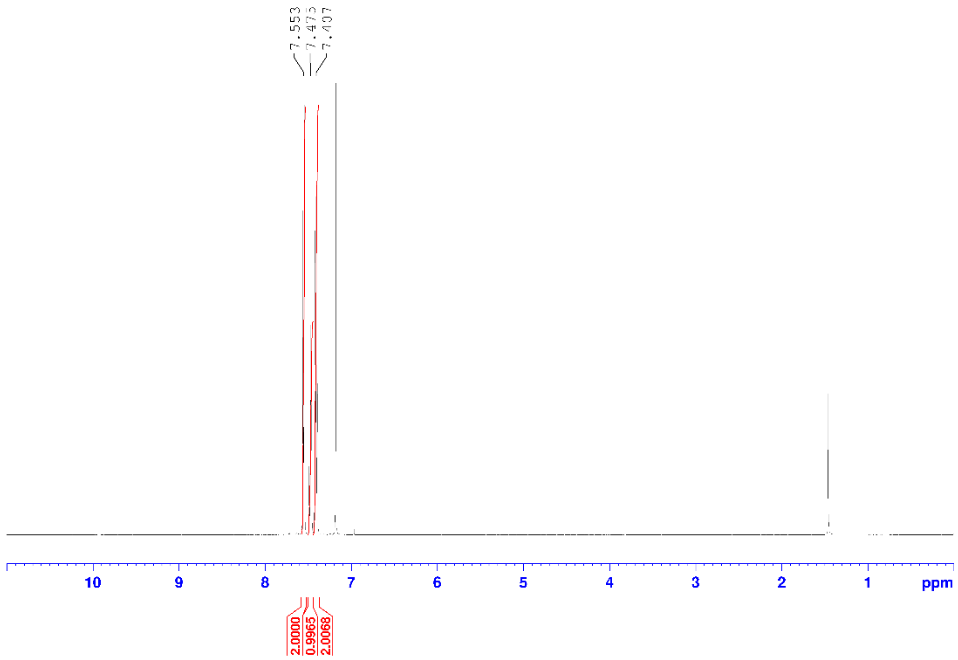

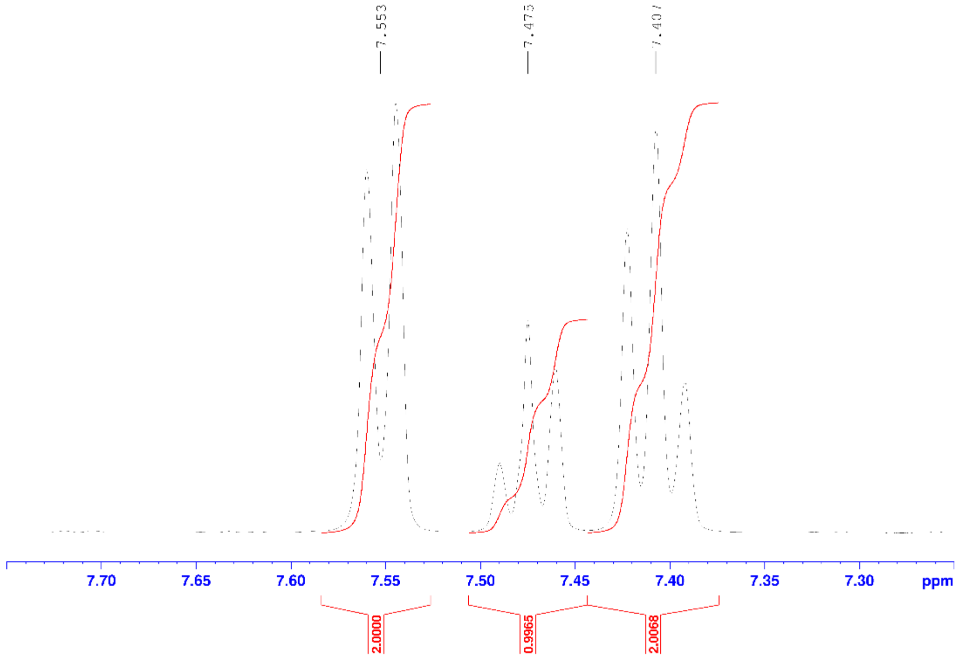

Figure S5: H[1] NMR of internal standard α,α,α-Trifluorotoluene

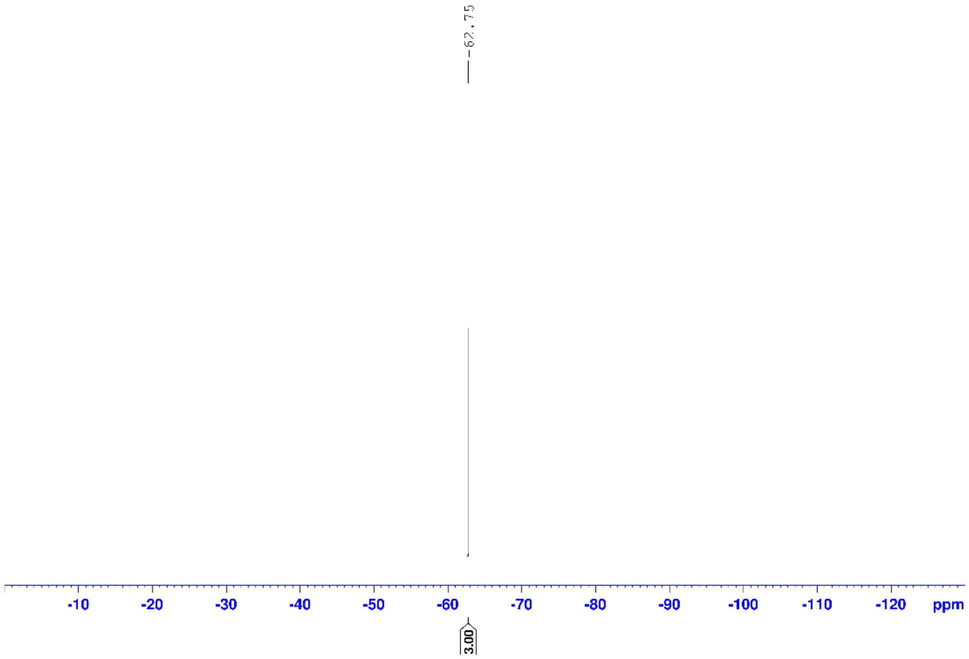

Figure S6: F[19] NMR of internal standard α,α,α-Trifluorotoluene

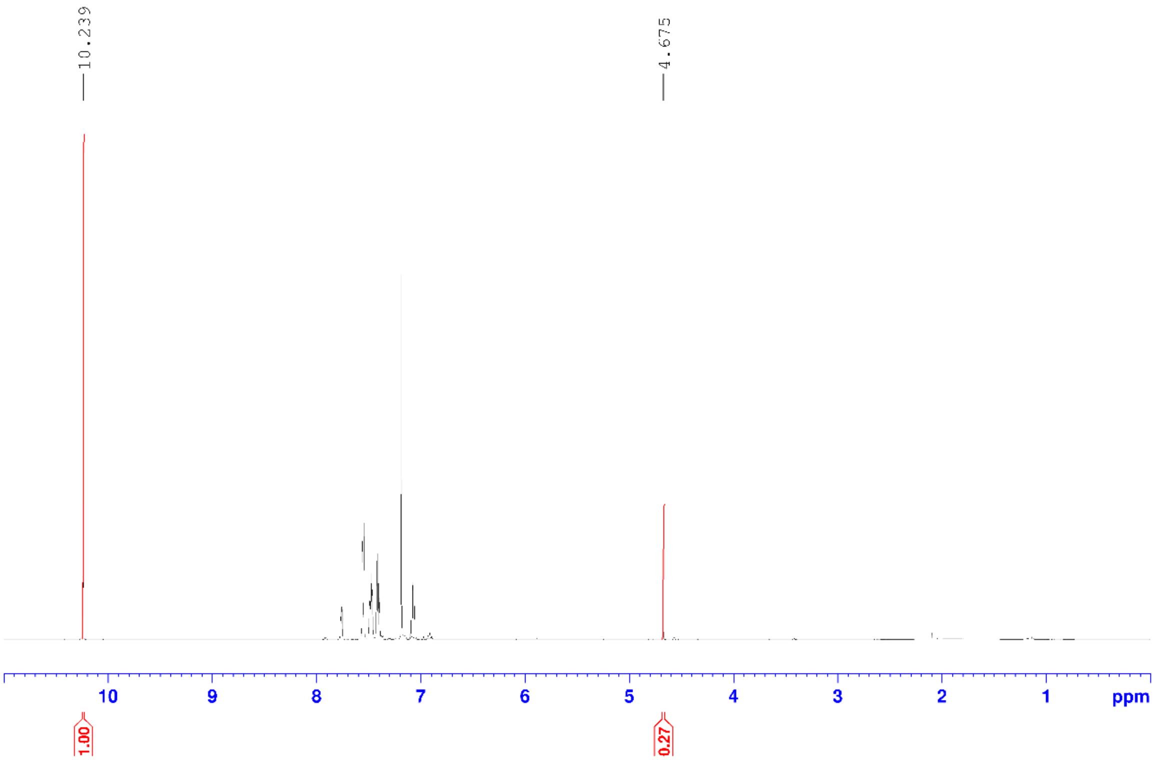

Figure S7: ChCl/Urea, narrow vessel, 40 ºC, ^{1}H NMR of reduction reaction mixture

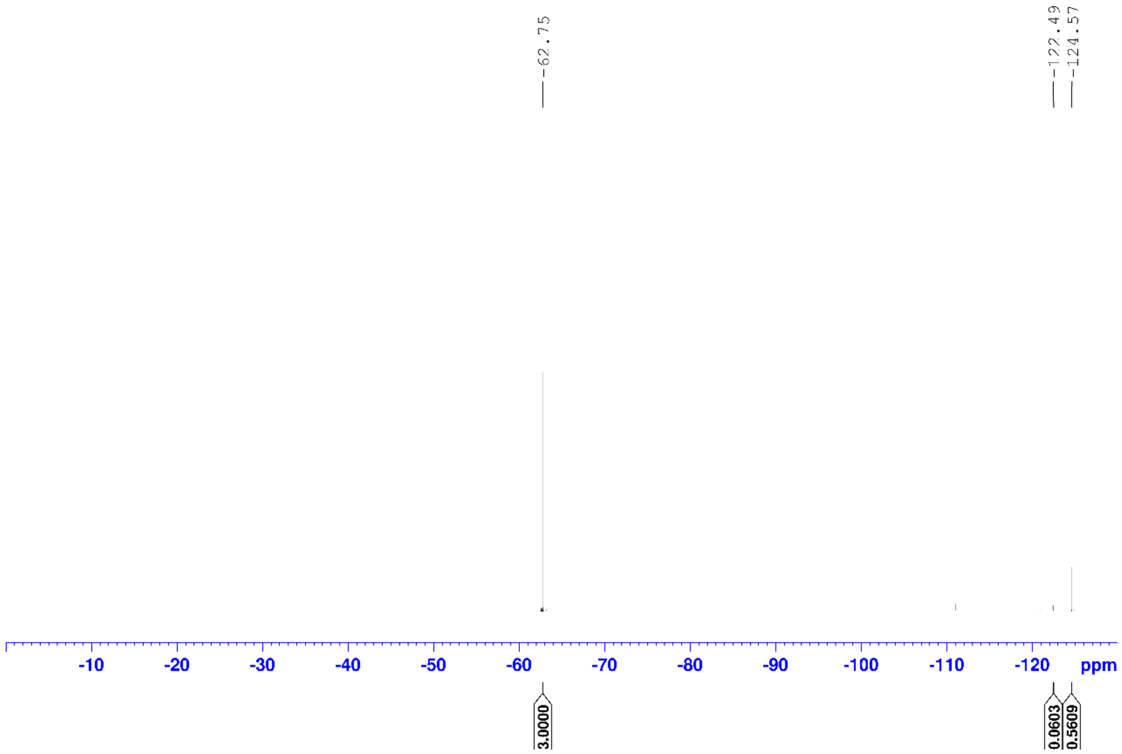

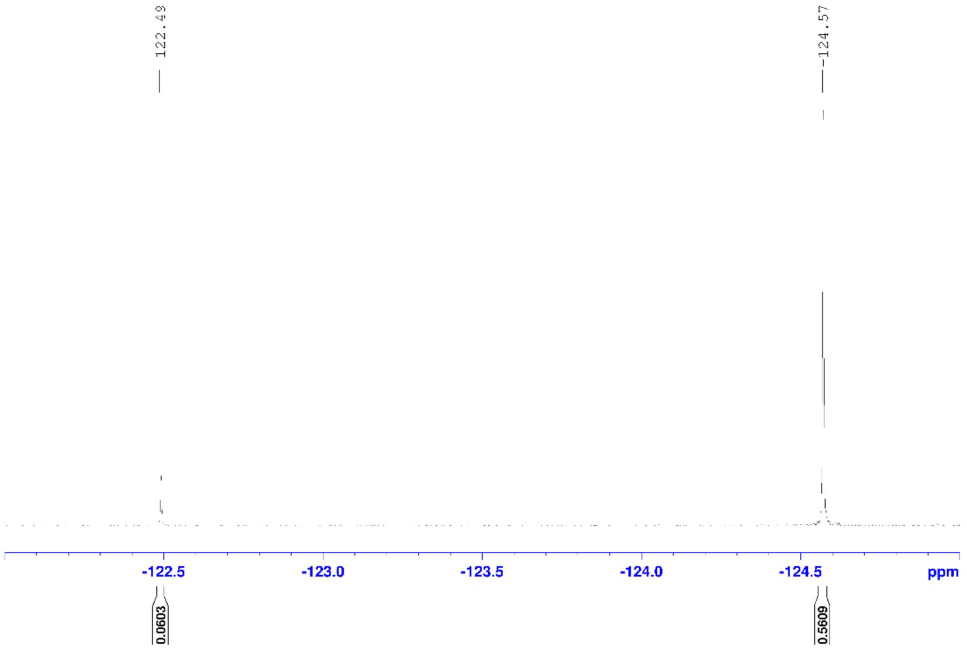

Figure S8: ChCl/Urea, narrow vessel, 40 ^{o}C, ^{19}F NMR of reduction reaction mixture

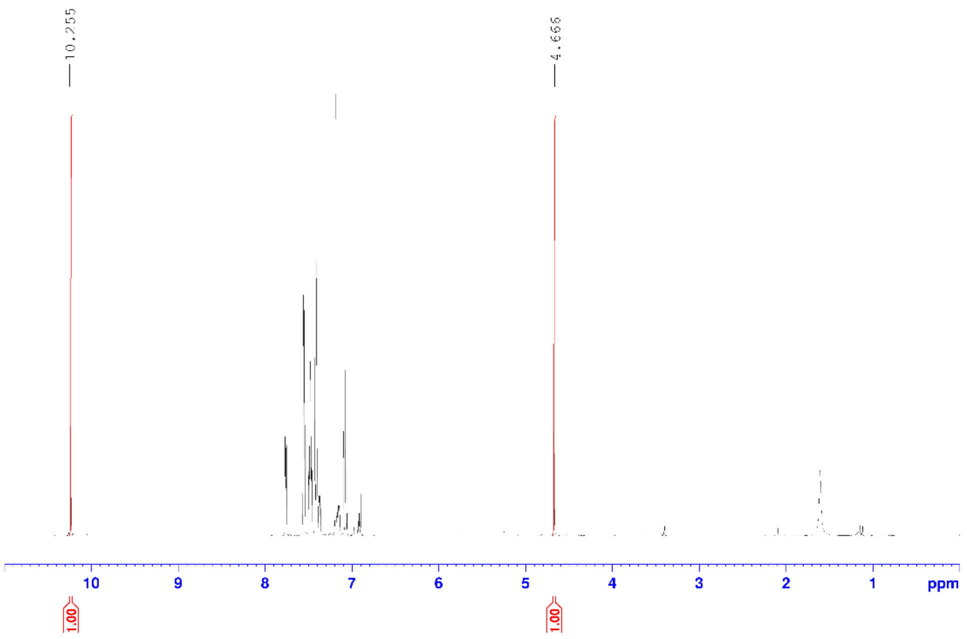

Figure S9: ChCl/Urea, narrow vessel, 40 ºC, ^{1}H NMR of reduction reaction mixture repeat 1

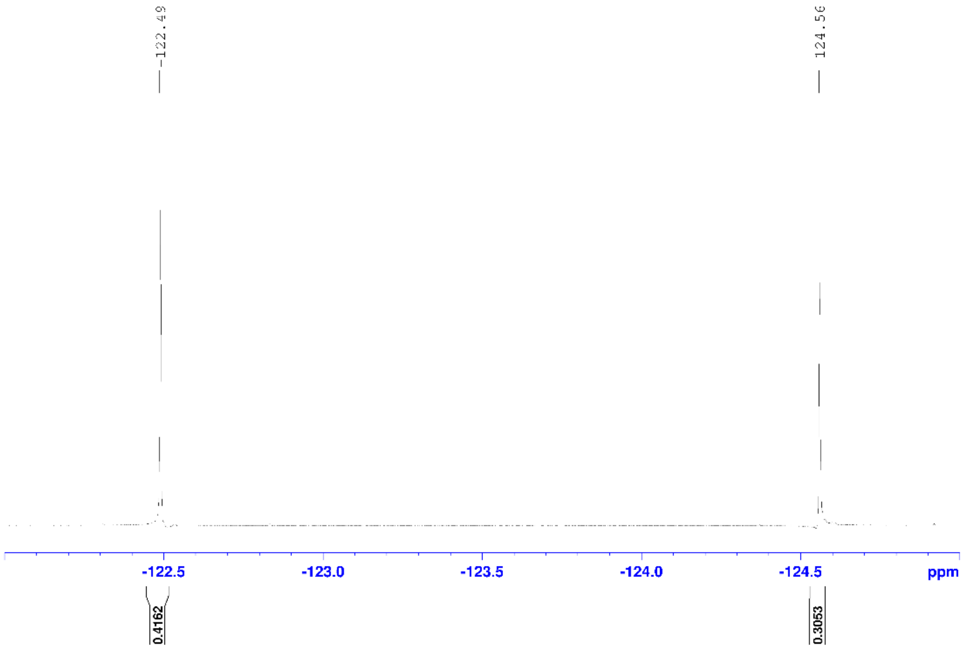

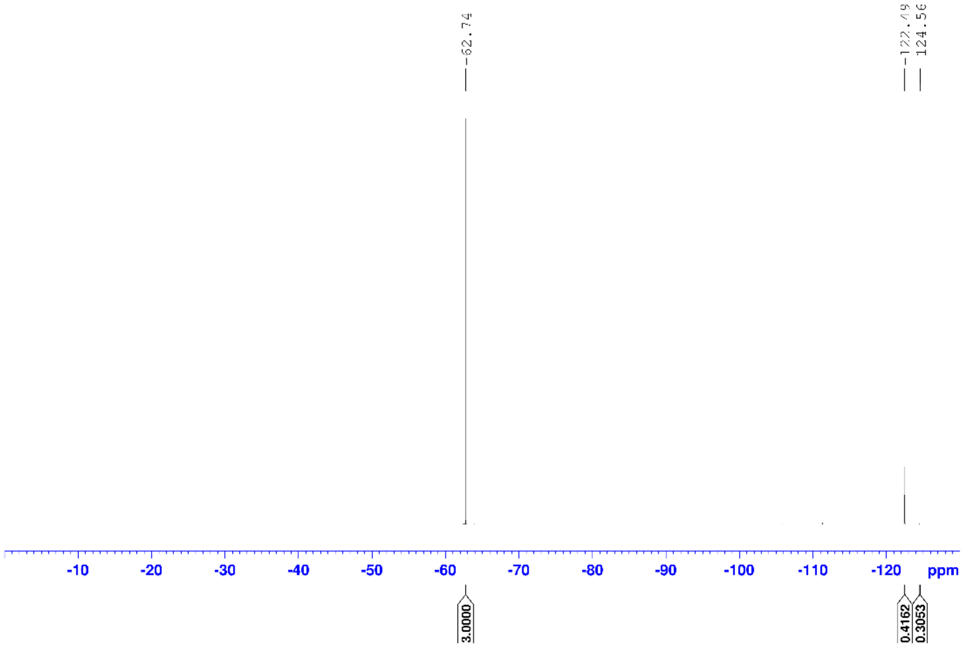

Figure S10: ChCl/Urea, narrow vessel, 40 ºC, ^{19}F NMR of reduction reaction mixture repeat 1

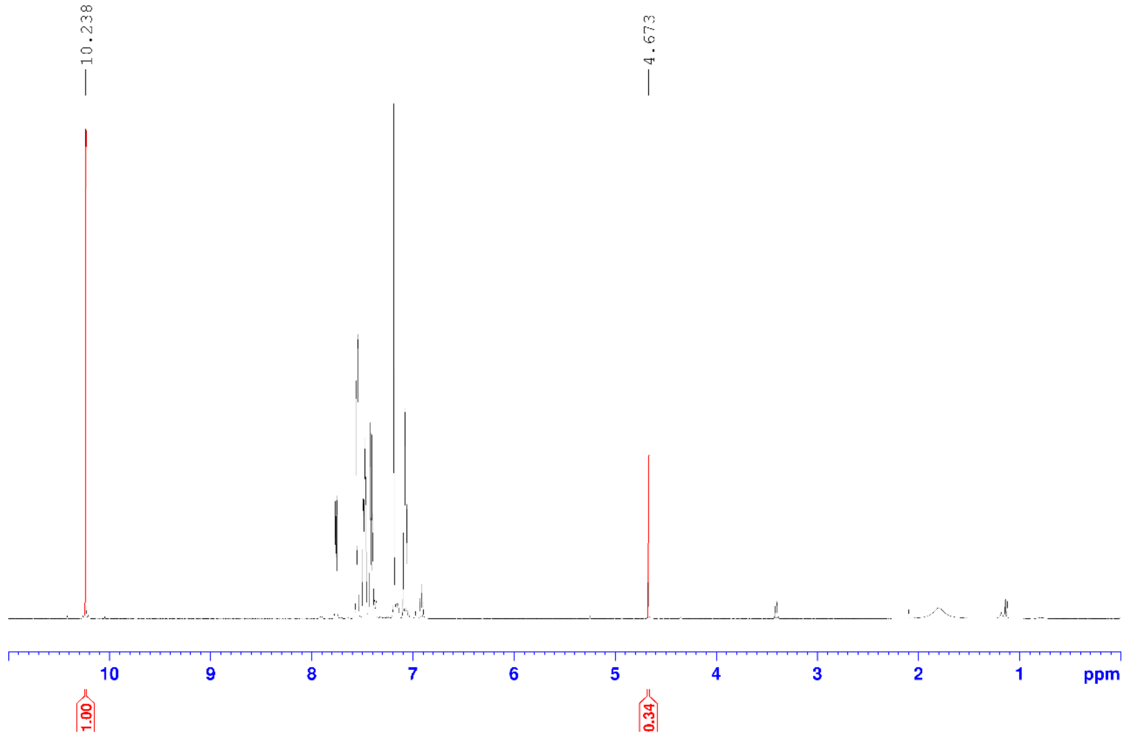

Figure S11: ChCl/Urea, narrow vessel, 40 ºC, ^{1}H NMR of reduction reaction mixture repeat 2

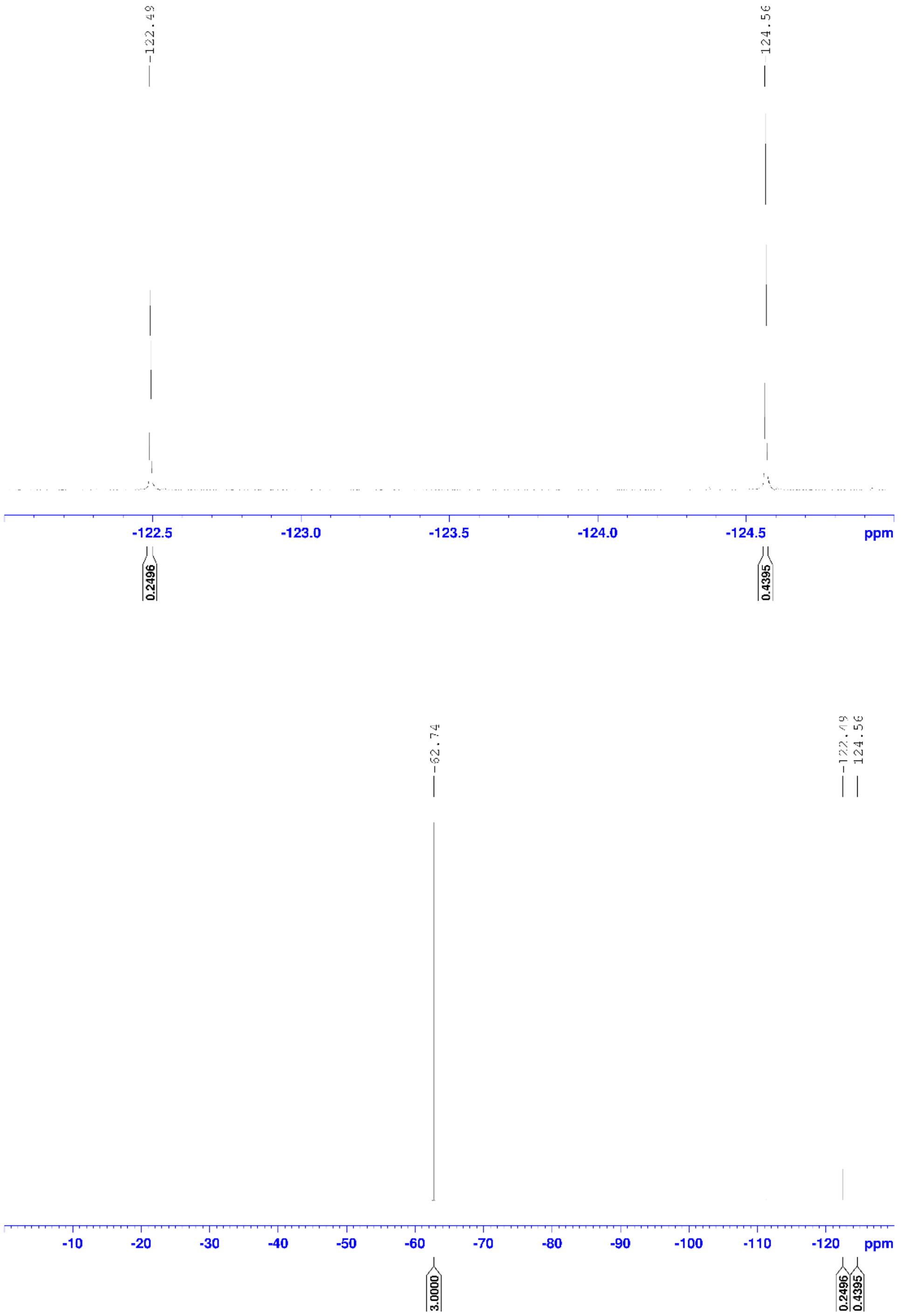

Figure S12: ChCl/Urea, narrow vessel, 40 ^{o}C, ^{19}F NMR of reduction reaction mixture repeat 2

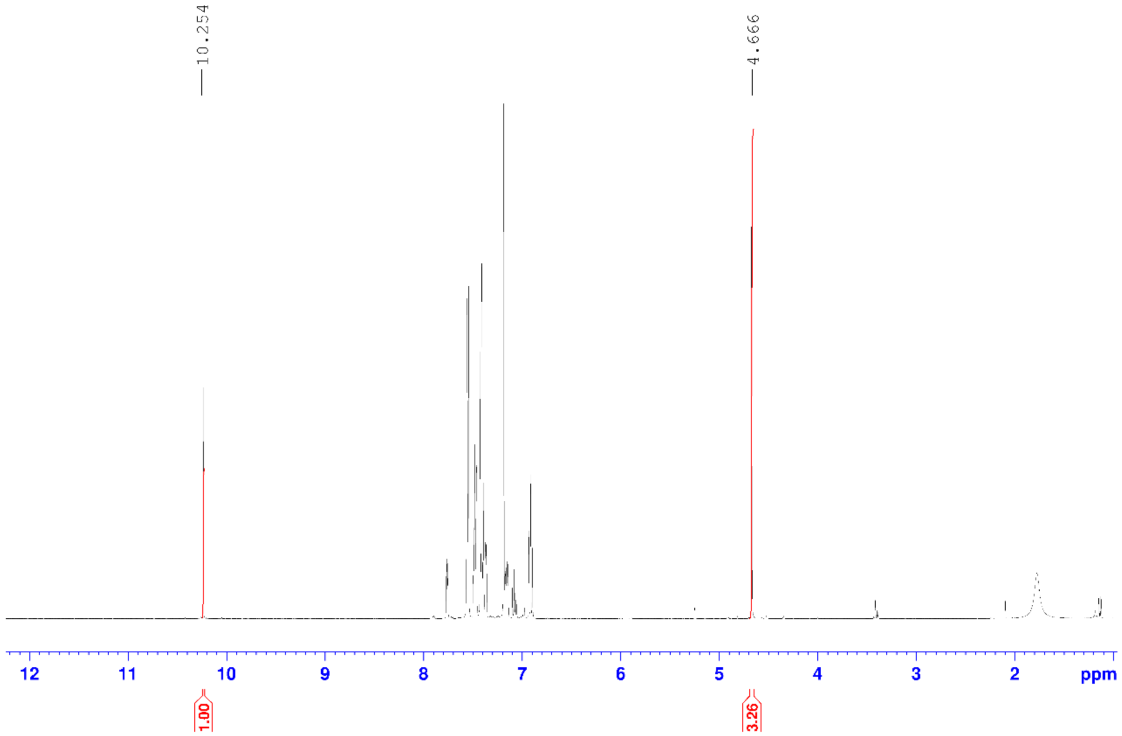

Figure S13: ChCl/Urea, wide vessel, 40 ºC, ¹H NMR of reduction reaction mixture

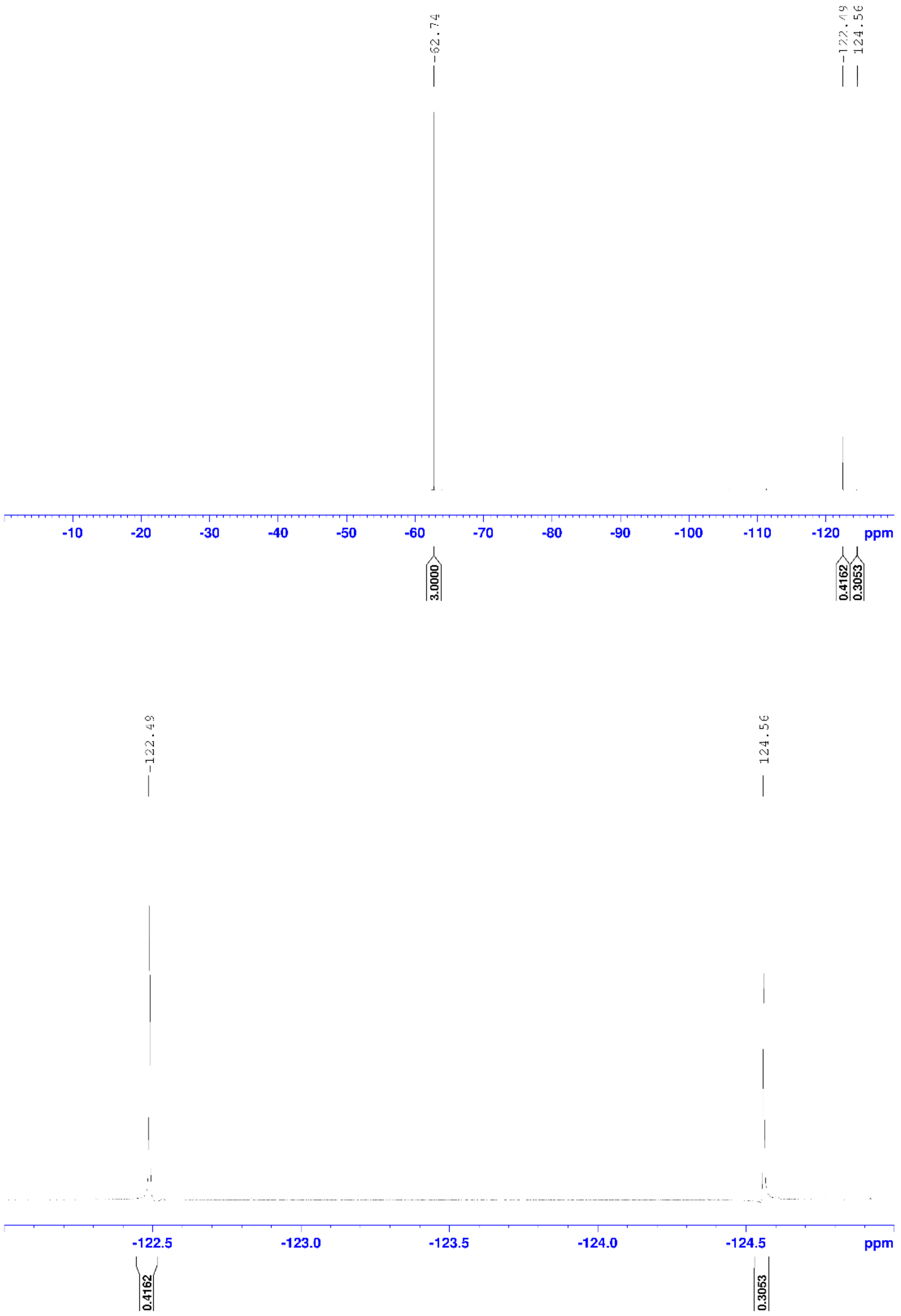

Figure S14: ChCl/Urea, wide vessel, 40 ^oC, ^{19}F NMR of reduction reaction mixture

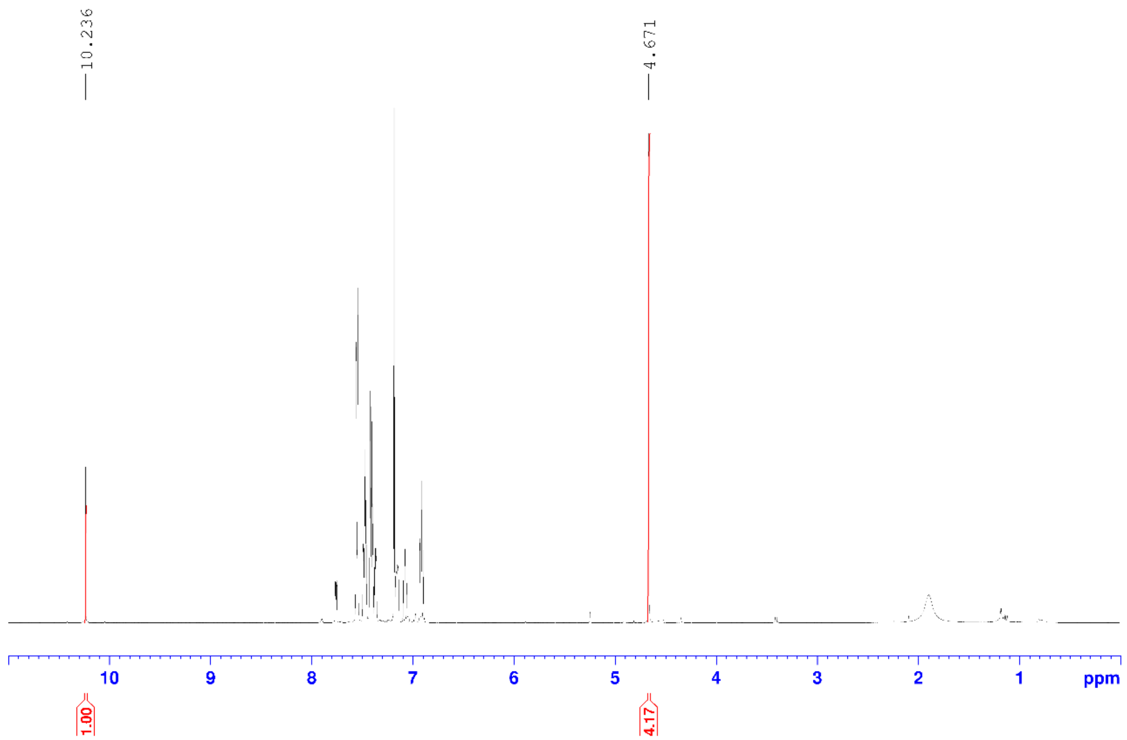

Figure S15: ChCl/Urea, wide vessel, 40 ºC, ^{1}H NMR of reduction reaction mixture repeat 1

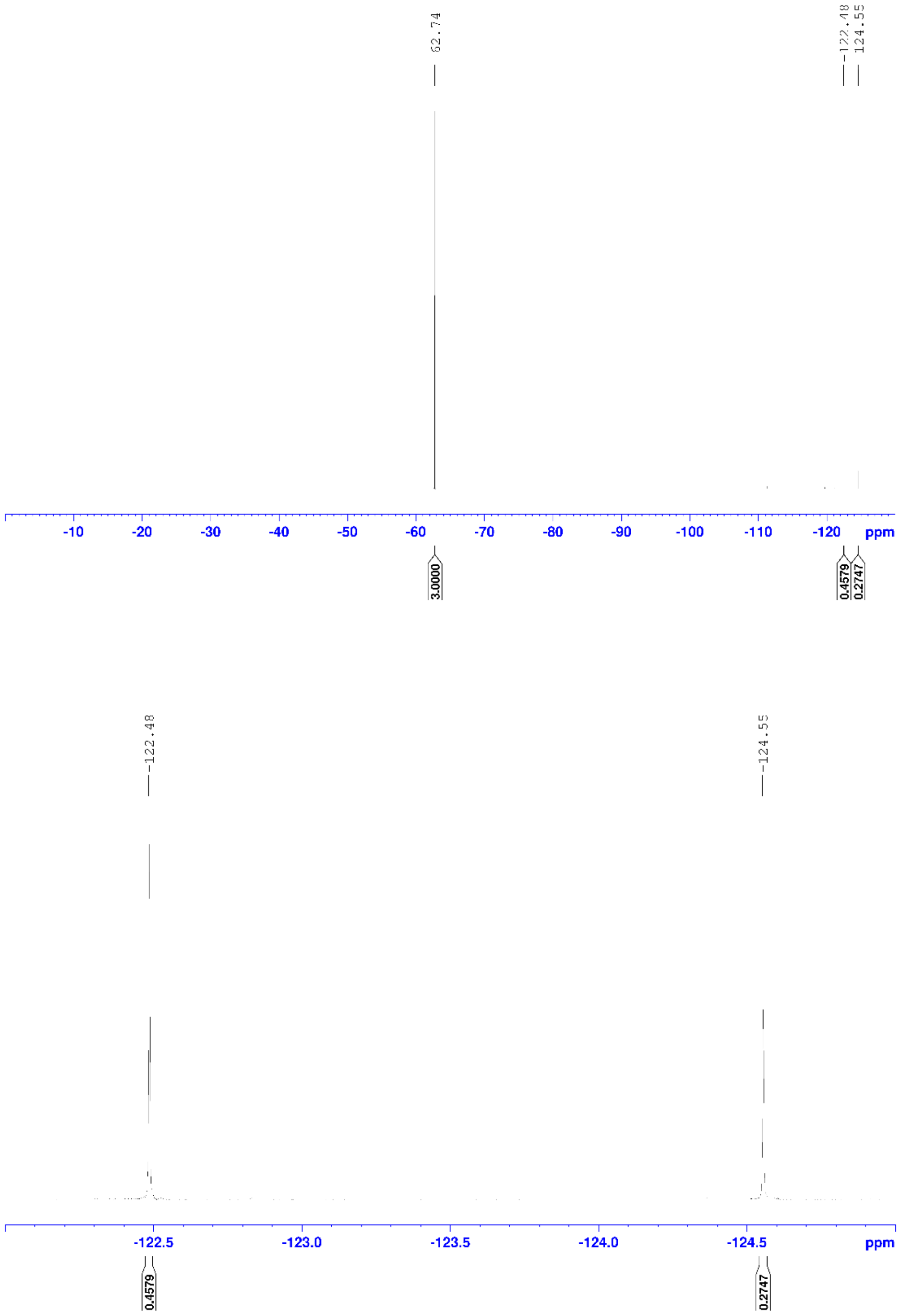

Figure S16: ChCl/Urea, wide vessel, 40 ºC, ^{19}F NMR of reduction reaction mixture repeat 1

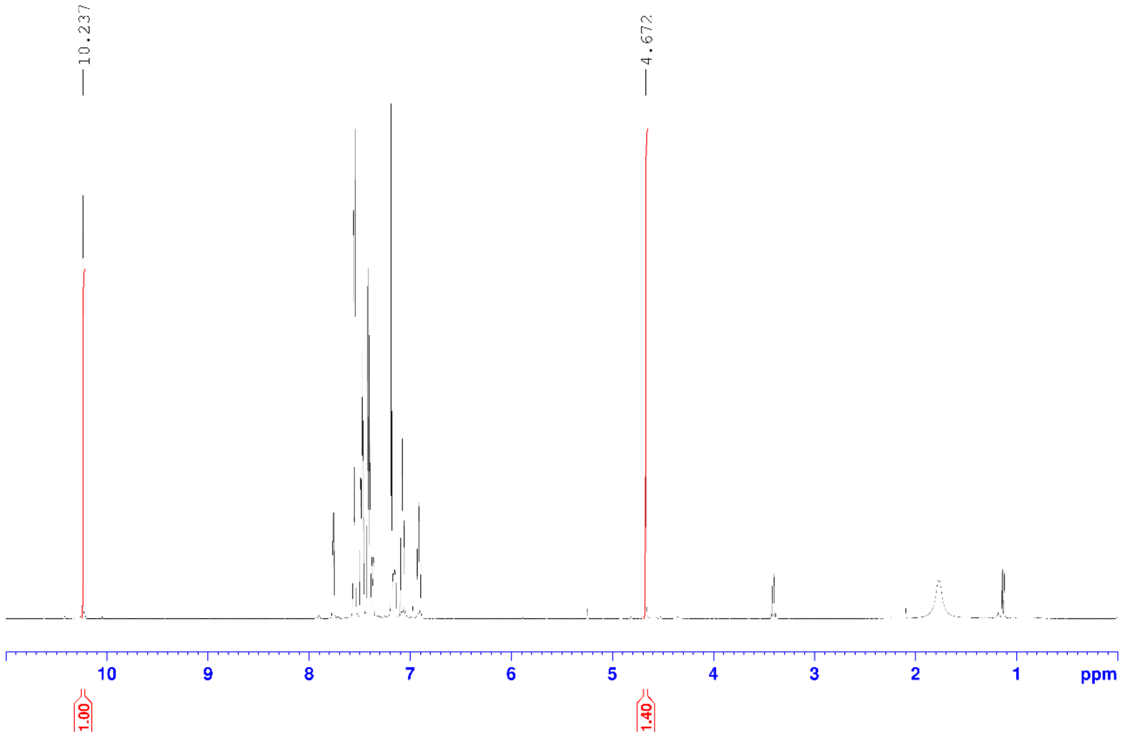

Figure S17: ChCl/Urea, wide vessel, 40 ºC, ¹H NMR of reduction reaction mixture repeat 2

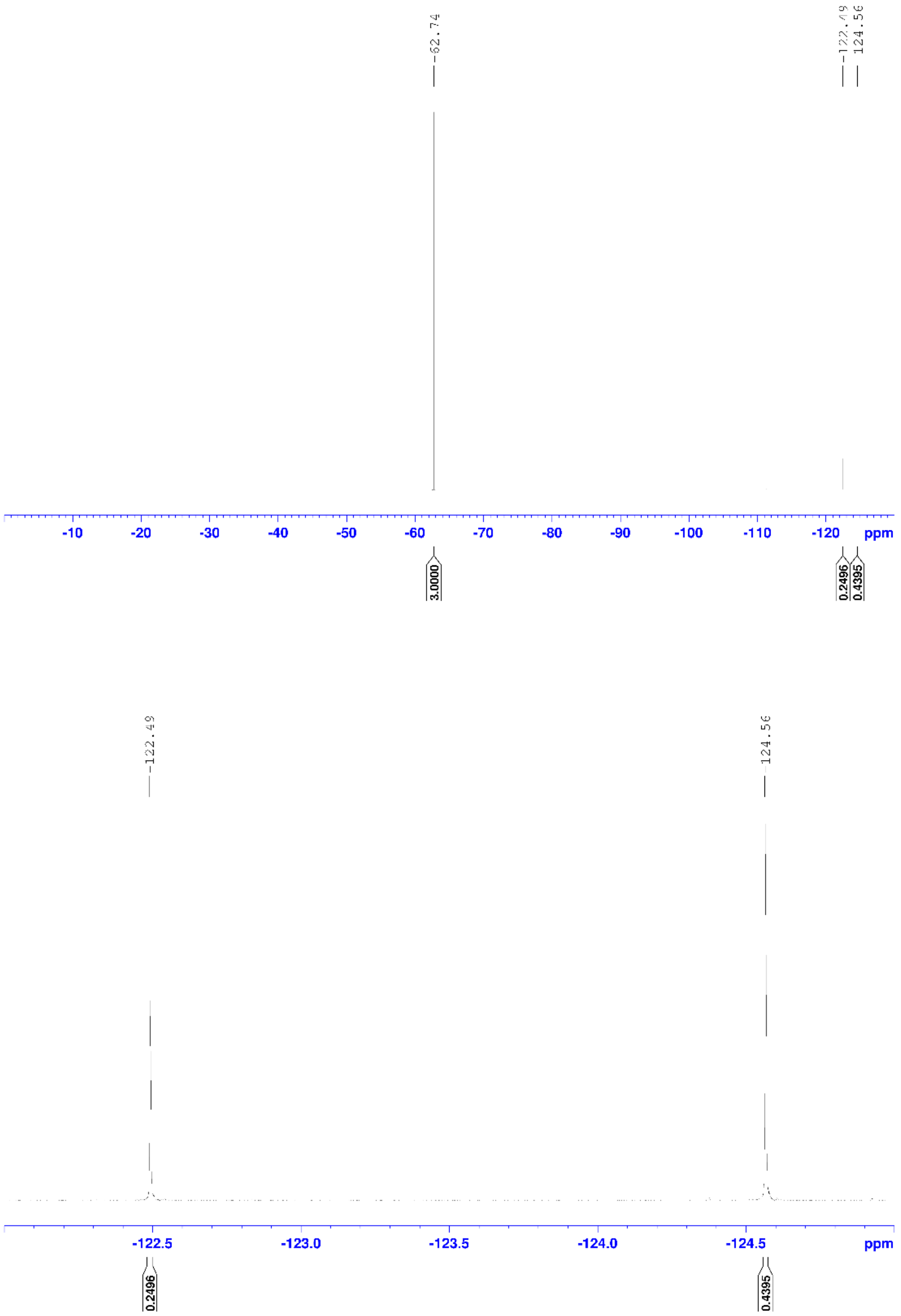

Figure S18: ChCl/Urea, wide vessel, 40 ^oC, ^{19}F NMR of reduction reaction mixture repeat 2

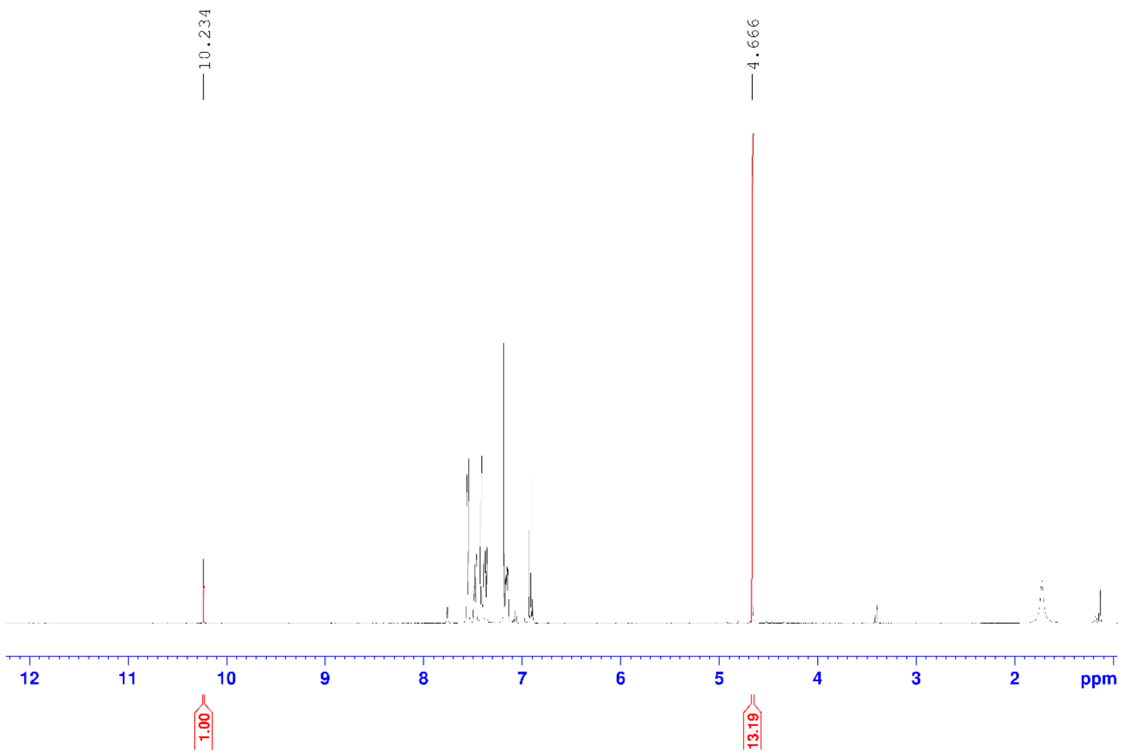

Figure S19: ChCl/Urea, wide vessel, 60 ºC, ^{1}H NMR of reduction reaction mixture

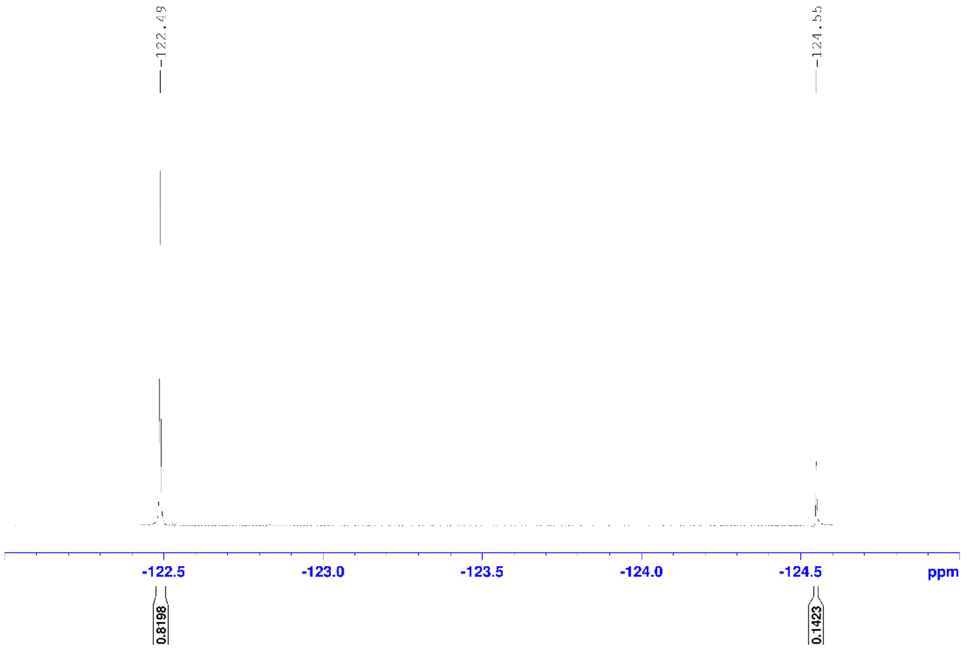

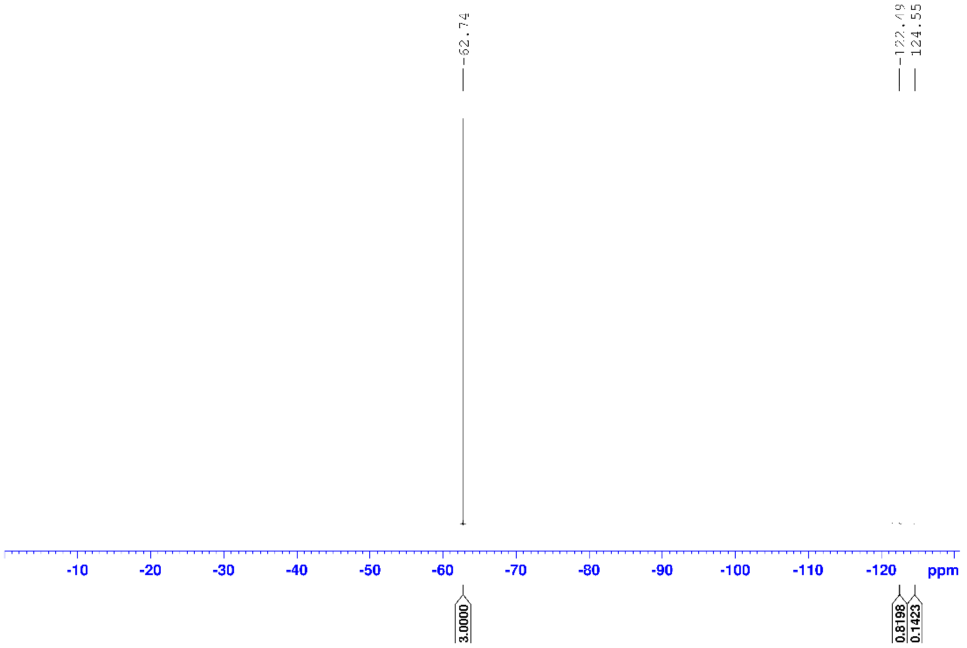

Figure S20: ChCl/Urea, wide vessel, 60 ^{o}C, ^{19}F NMR of reduction reaction mixture

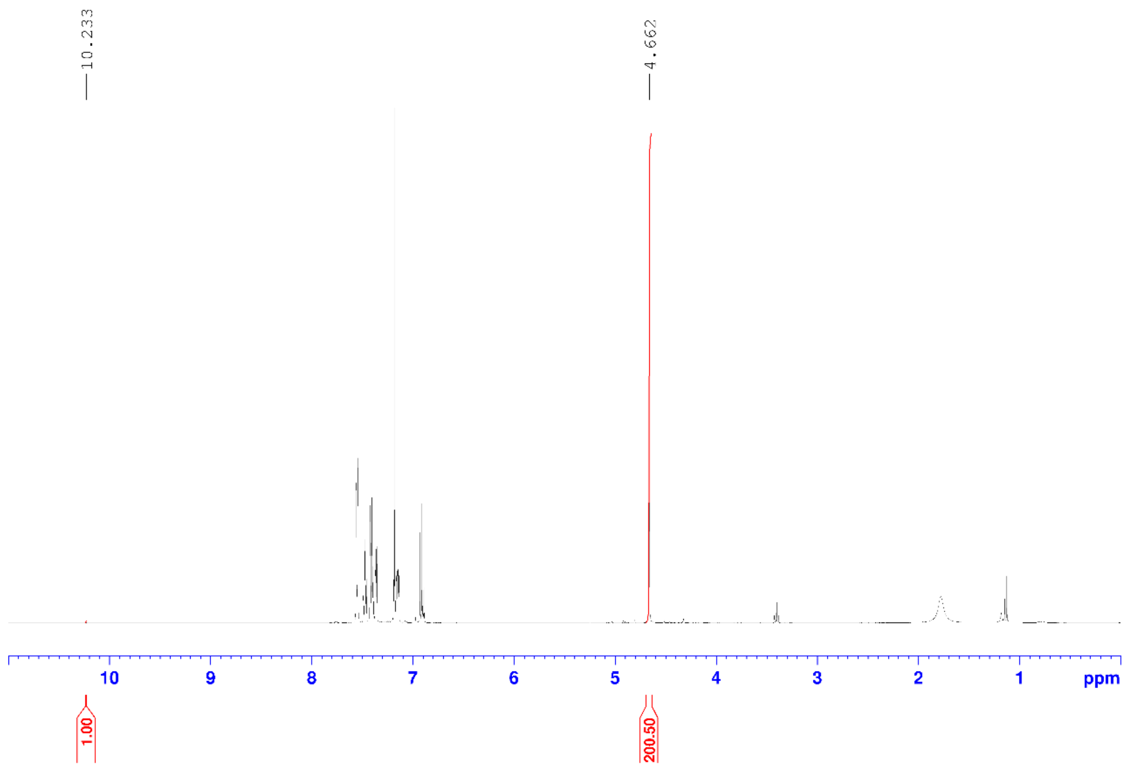

Figure S21: ChCl/Urea, wide vessel, 60 ºC, ¹H NMR of reduction reaction mixture repeat 1

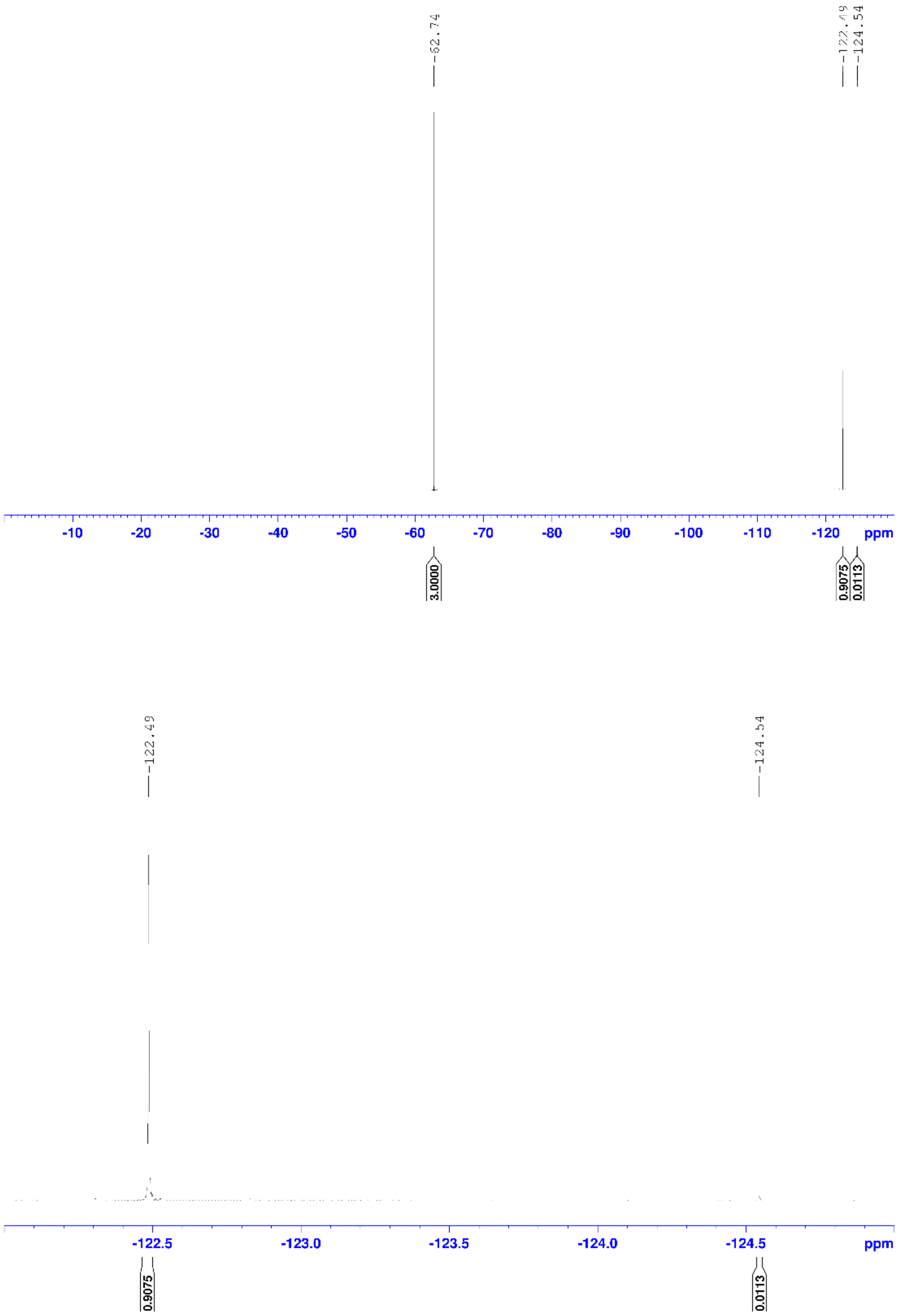

Figure S22: ChCl/Urea, wide vessel, 60 ^{o}C, ^{19}F NMR of reduction reaction mixture repeat 1

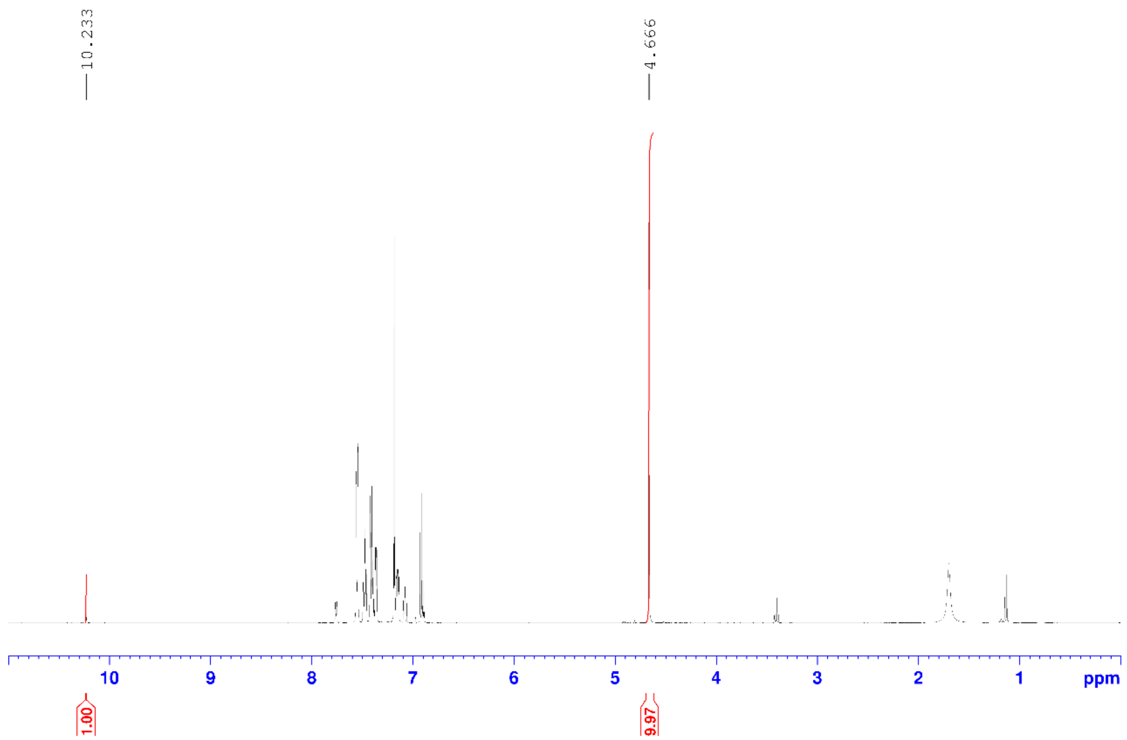

Figure S23: ChCl/Urea, wide vessel, 60 ºC, ¹H NMR of reduction reaction mixture repeat 2

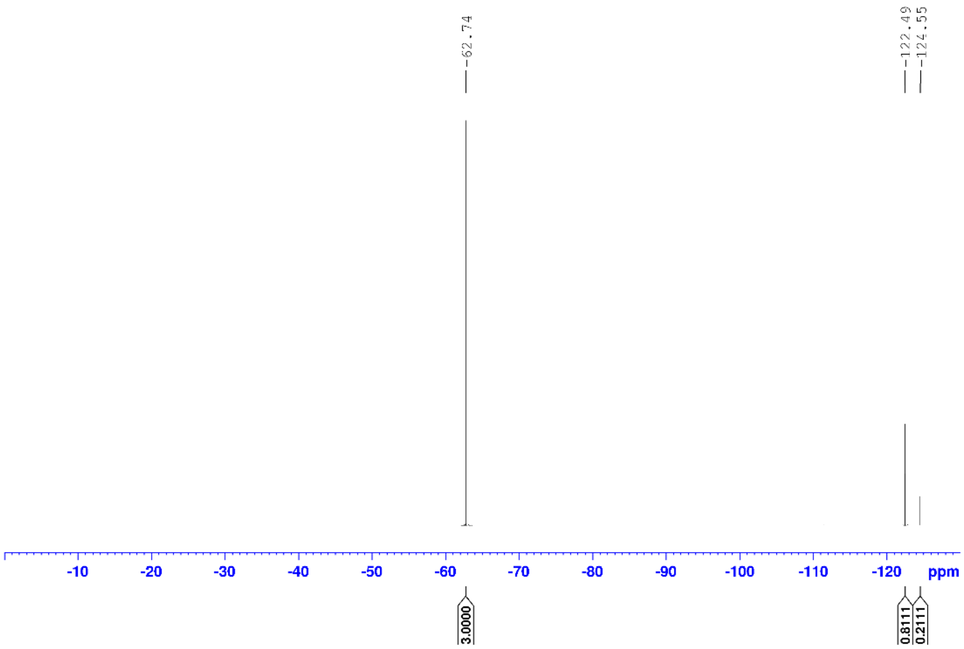

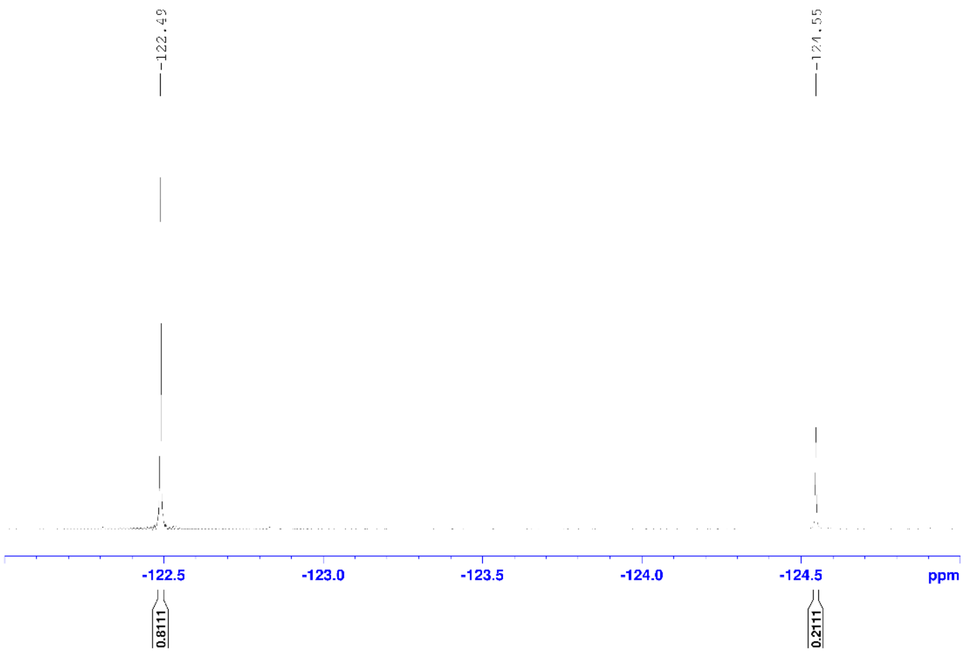

Figure S24: ChCl/Urea, wide vessel, 60 ºC, ^{19}F NMR of reduction reaction mixture repeat 2

Computational Details

Computational Fluid Dynamics Modelling with Ansys R17.1

Geometry

The two vessel geometries, wide and narrow Schlenk tubes, used in the CFD calculations were constructed using the Ansys Design Modeler. Taking advantage of the radial symmetry, the general strategy was to create a 2D sketch that could be revolved around the center line to give the desired 3D geometry. A set of digital calipers was used to obtain the necessary dimensions from the glassware and stir bar. The stir bar (12 x 5 mm) sketch was created by modifying an oval created from the oval drawing tool, producing a 'half oval'. The wide/large Schlenk was assumed to be a partially filled sphere, and the narrow/small Schlenk was assumed to be a cylinder with a hemispherical bottom. The outer diameters were obtained using digital calipers measuring 37.6 mm and 17.5 mm, respectively. A glass thickness of 1.2 mm was assumed based on some broken lab glassware. Giving an internal radius of 17.6 mm and 7.55 mm, respectively. The fill height in both cases was adjusted to ensure the final volume of the fluids to be modelled was 5 ml. Rather than using a measured height, this approach accounted for differences due to the assumptions made about the vessel and stir bar geometry. Following this, the stir bar was positioned appropriately in the two vessels. It is acknowledged that this is where the geometries deviate from reality; the stir bar is suspended in the vessels, therefore it doesn't spin about the bottom of the vessels as it would normally do. Nevertheless, it provides an insightful qualitative comparison to the experimental data. The region that the stir bar directly influences is defined by a cylinder primitive.

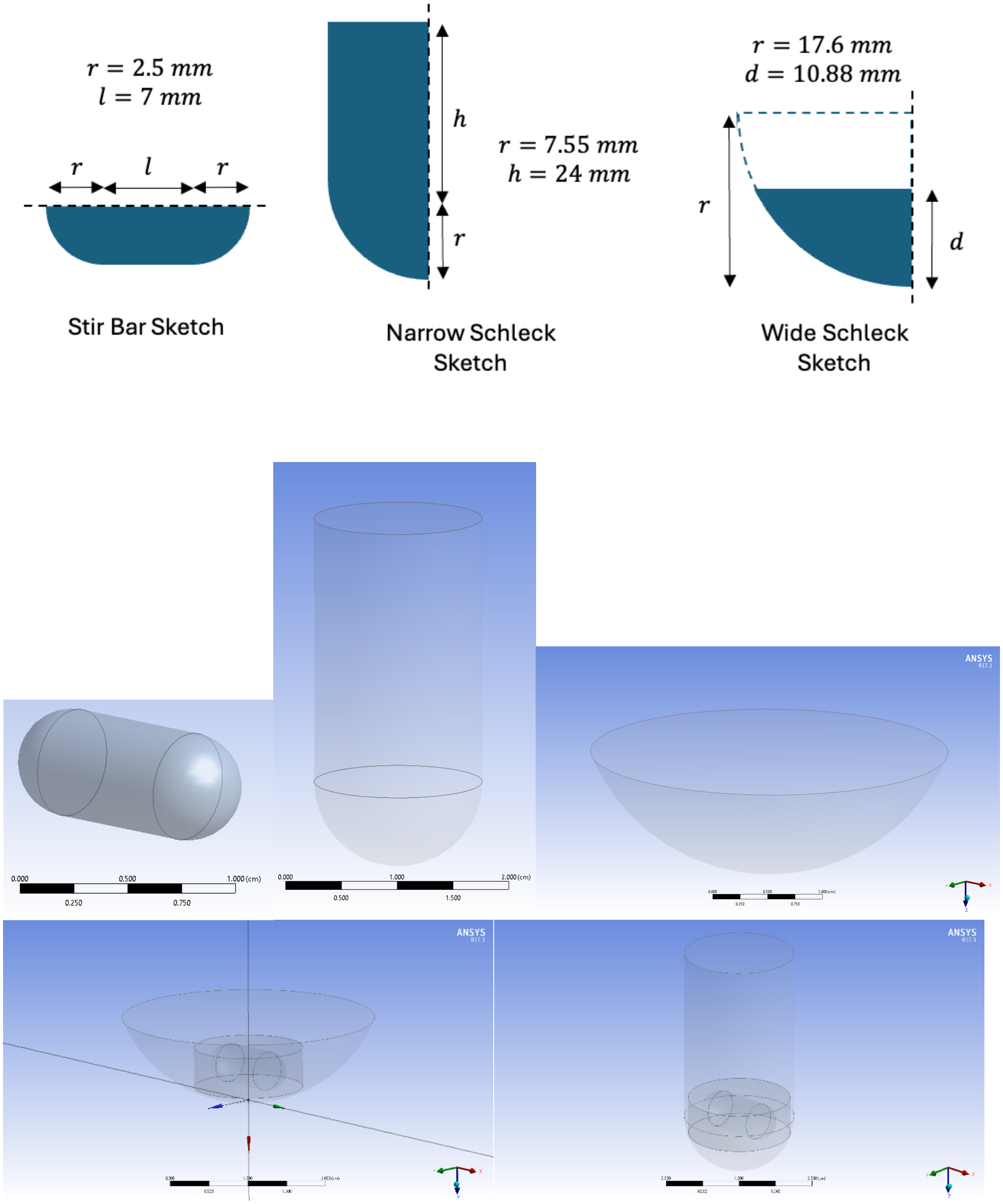

Figure S25: Final geometry for wide and narrow Schlenk tubes.

Meshing

Ansys automatic meshing was used, Proximity and curvature size function, fine relevance center all other values default. Mesh quality was assessed looking at the aspect ratio (< 5), skewness (< 0.7), and orthogonal quality (> 0.3):

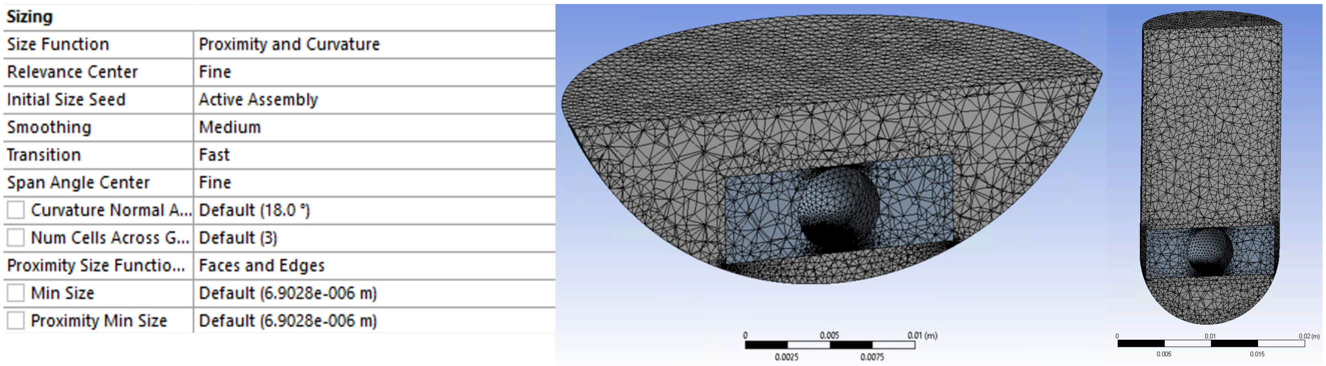

Figure S26: Meshing settings and resultant mesh for the wide and narrow setups.

Table S4: Mesh statistics.

			Aspect Ratio		Skewness		Orthogonal quality	
**Setup**	Nodes	Elements	Average	Std	Average	Std	Average	Std
**Wide Geometry**	1011599	678764	1.8555	0.4394	0.23466	0.12669	0.85703	7.9947e-002
**Narrow Geometry**	1711595	1151201	1.854	0.4376	0.2344	0.12739	0.85734	7.9666e-002

Setup

All simulations used double precision, transient, gravity, k-omega SST viscous model, and coupled solution method. Inner Domain rotating at 700 rpm. Simulations ran for 60 s flow time at 0.05 s time steps for DESs, MeOH ran for 10 s flow time at 0.001s time steps. Solvent viscosity and density for each solvent shown in Table S5:

Table S5: Table of solvent viscosity (cP) and density (g/cm^3) used in the CFD simulations.

Solvent	viscosity, cP	density, gcm^{-3}
**MeOH**	0.5495	0.785
**EG_60 [1]**	13.22	1.09637
**EG_40 [1]**	24.24	1.10761
**EG_20 [1]**	52.55	1.11897
**G_60 [2]**	53.8117	1.1713
**U_60 [2]**	68.6478	1.1773
**G_40 [2]**	133.3749	1.1827
**U_40 [3]**	238.0763	1.1887
**G_20 [3]**	472.9671	1.1943
**U_20 [3]**	1371.9719	1.2001

[1] https://doi.org/10.1021/acs.jced.3c00222
[2] https://doi.org/10.1016/j.fluid.2014.01.028
[3] https://doi.org/10.1021/je5001796

Plateau Analysis

The methods reported herein were first reported by our group in the following publication:

Chem. Sci., **2023**,*14*, 11872-11880, DOI: https://doi.org/10.1039/D3SC01383A, and supporting information therein.

The underlying principle of the plateau detection method is plateau is a region of little or no change; the 1st order differential will be approximately zero. Employing numerical differentiation, interpreted as calculating the gradient between two adjacent points, noise is amplified when values and associated error are of similar magnitude. This is often the case at a plateau when changes in the y-axis values are small. Data smoothing via rolling averages was employed to minimise the impact of noise. When the rolling average of the gradient is below a defined threshold value, it may be in a plateau region. A plateau region was formally defined if a user-defined number of consecutive points were below the threshold rate-of-change value.

Python code associated with plateau analysis is provided in the machine-readable supporting information zipped folder. See figshare **DOI: 10.6084/m9.figshare.29279894**

Additional Visualizations

Proof-of-concept – tracking mixing effects in viscous media

Beyond the ΔE visualizations supplied in manuscript Figure 7, below are additional outputs from Kineticolor analysis of the proof-of-concept phenylphthalein titrations.

Contact (thresholding-based metric)

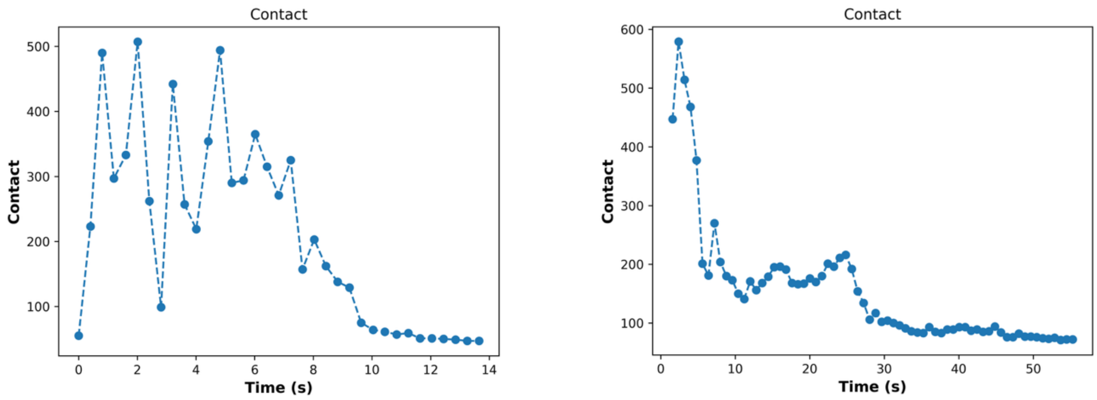

Figure S27.

The thresholding-based contact metric shows pronounced differences in particle interaction behavior. The non-viscous medium (left) exhibits dramatic spikes reaching 500+ contact events with high variability in the initial phase, followed by a steady decline to baseline levels around 50. The viscous system (right) demonstrates a more controlled contact pattern, starting high around 600 but smoothly decreasing to stabilize around 80-100 contacts, suggesting more consistent particle interactions due to the increased medium viscosity.

Contrast (texture-based metric)

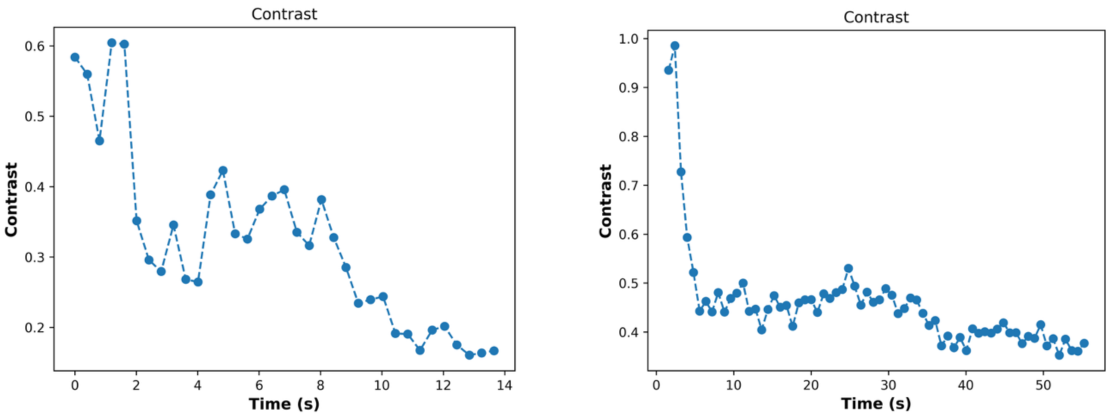

Figure S28.

Contrast measurements highlight the textural differences between reaction conditions. The non-viscous system (left) begins with high contrast values around 0.6, experiences several peaks and valleys in the early stages, then progressively decreases to stabilize around 0.16. The viscous system (right) starts with extremely high contrast near 1.0 but rapidly decreases and maintains relatively stable, lower values between 0.4-0.5 throughout most of the observation period.

Angular Second Moment (a.k.a. Energy; texture-based metric)

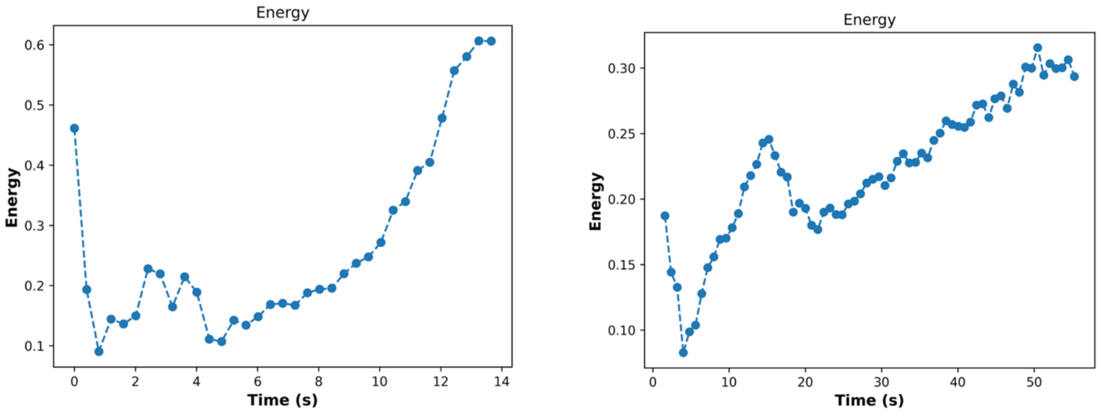

Figure S29.

The energy metric reveals markedly different patterns between the two systems. The non-viscous reaction (left) shows initial volatility with energy dropping to approximately 0.09 before steadily climbing to 0.6, indicating increasing texture uniformity over time. In contrast, the viscous medium (right) exhibits a more controlled progression, starting around 0.08, peaking briefly at 0.25, then gradually ascending to 0.30 with smoother transitions.

Homogeneity (texture-based metric)

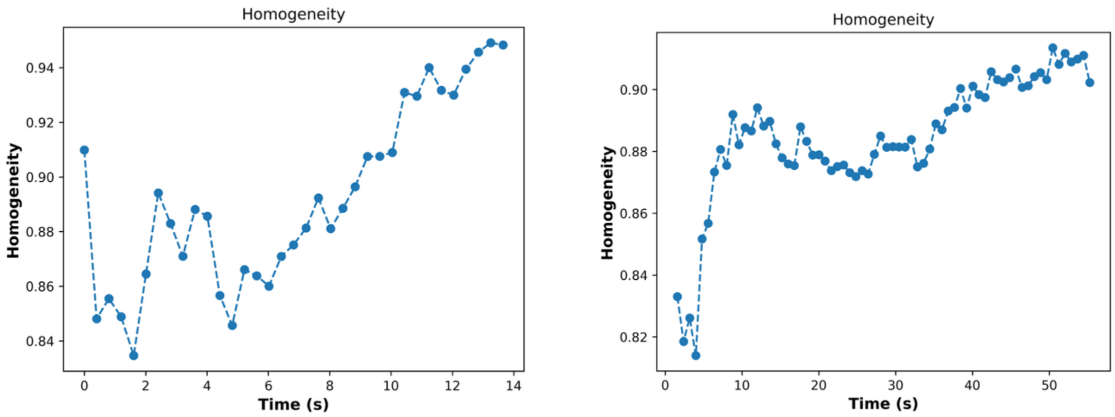

Figure S30.

The homogeneity metric shows distinct behavior between non-viscous and viscous reaction media. In the non-viscous system (left), homogeneity exhibits significant fluctuations early on, dropping to around 0.84 before gradually increasing to stabilize above 0.94 by the end of the observation period. The viscous system (right) demonstrates more stable homogeneity values, starting around 0.82 and steadily increasing to plateau near 0.92, with less dramatic fluctuations throughout the process.

Dye mixing measurements with DESs: cuvette studies

As temperature increased, the row-specific ΔE profiles converged more rapidly, demonstrating improved mass transport throughout the vessel volume. Figure S29 shows the ΔE by row for the three DES formulations ChCl:EG, ChCl:G, and ChCl:U at three temperatures, 25 °C, 40 °C, and 60 °C.

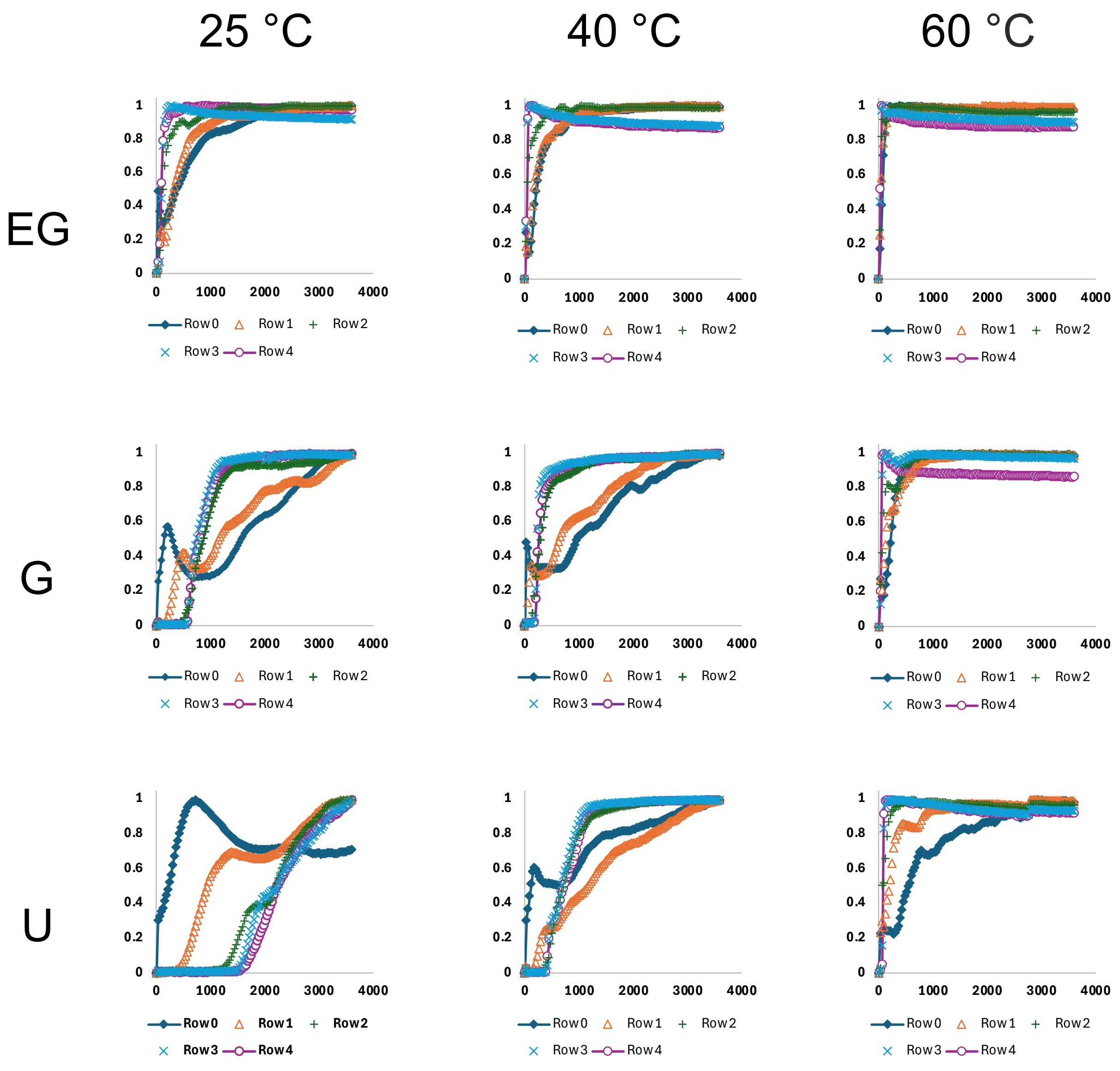

Figure S31: Plots of ΔE by row (row 0 top of the cuvette, row 4 bottom of the cuvette) for DES formulations ChCl:EG, ChCl:G, and ChCl:U at three temperatures, 25 °C, 40 °C, and 60 °C. ΔE profiles converge faster at higher temperatures.

Computational Fluid Dynamics

Figure S30 shows the complete vertical velocity at the centerline for both the wide and narrow vessels. Showing how the vertical velocity changes as you move away from the stir bar towards the top surface of the vessels.

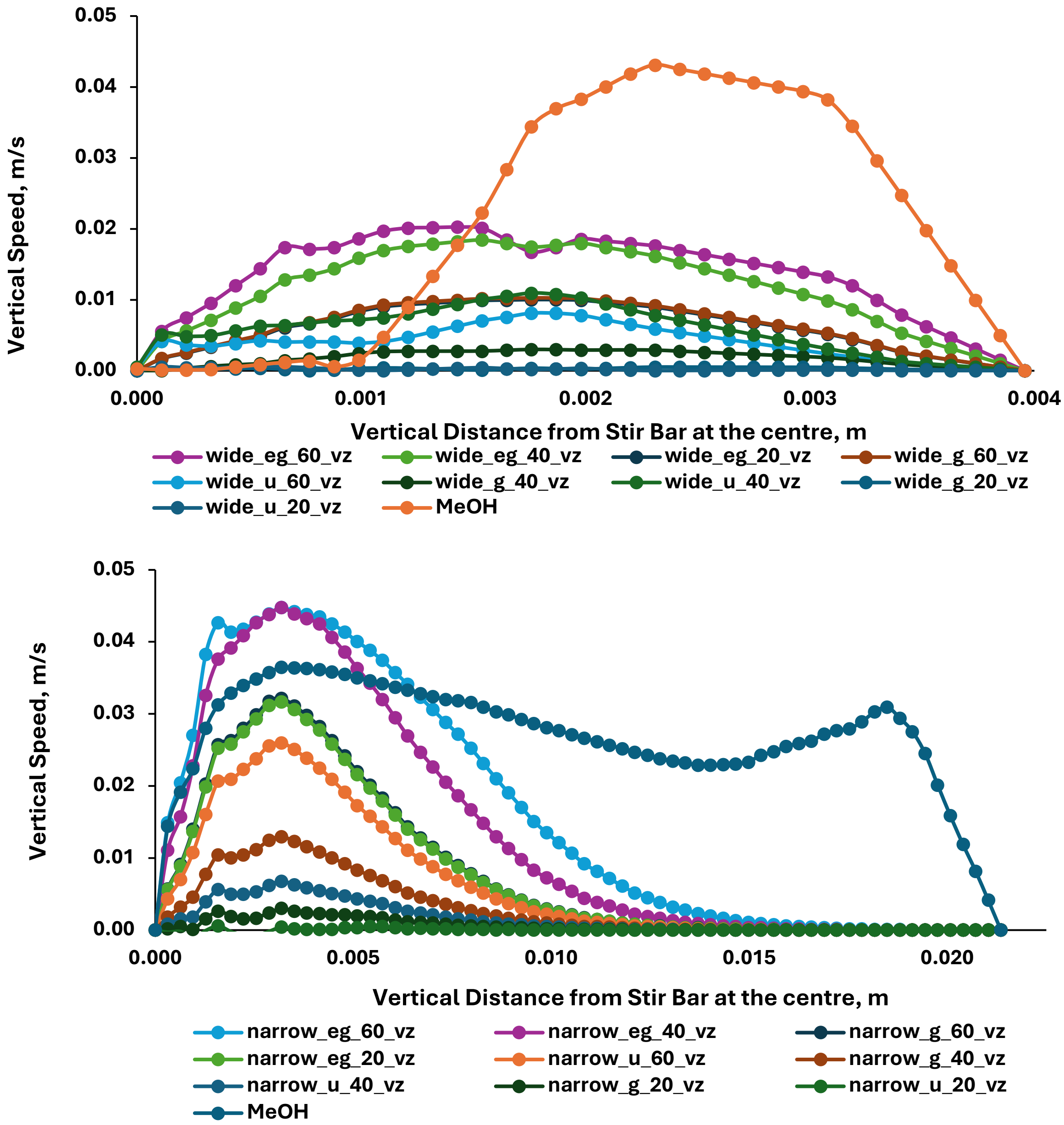

Figure S32: vertical velocity at the centerline for the wide vessel (Top) and narrow vessel (bottom). The larger the solvent viscosity, the lower the vertical velocity magnitude and the distance over which the stir bar exerts an influence.

Examining the component velocities reveals more details than the overall velocity contour plots. Component x, y, and z velocities for selected solvents are shown in Figure S30. With increasing viscosity, there is a decrease in the y and z component velocities, resulting in less turbulence and poor mixing. All show the same x-component structure indicative of the rotational motion of the stir bar.

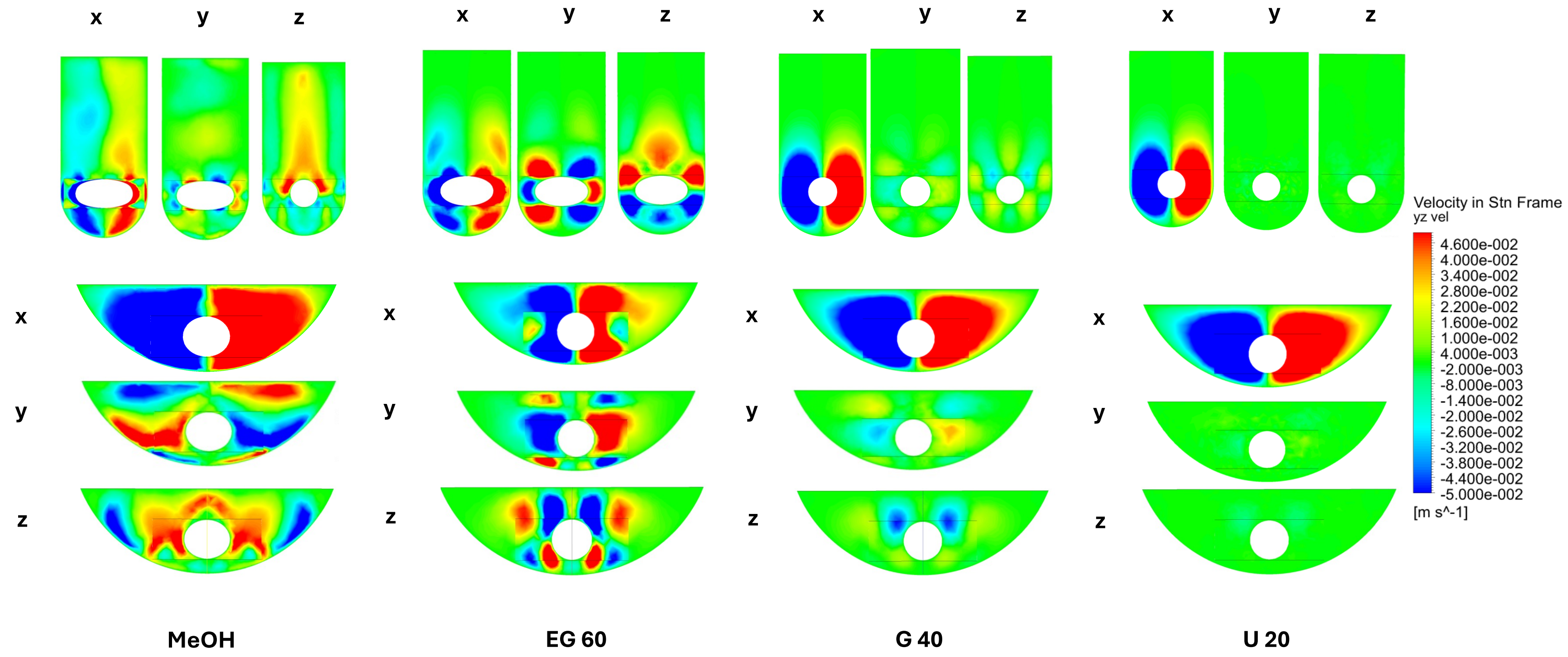

Figure S33: Component x (into and out of the page), y (left and right) , and z (up and down) velocity contour plots for MeOH at 20 °C, ChCl:EG at 60 °C, ChCl:G at 40 °C, and ChCl:U at 20 °C, showing the diminishing y and z components as viscosity increases.

Using streamlines, we visualized the complex flows. Streamlines for selected solvents are shown in Figure S32. In the more viscous cases, the streamlines are parallel, indicating more laminar flow, less turbulence, and poor mixing. For MeOH, there is an evident and strong vortex. The streamline structures from CFD exhibit a similarity to what is observed in the videos.

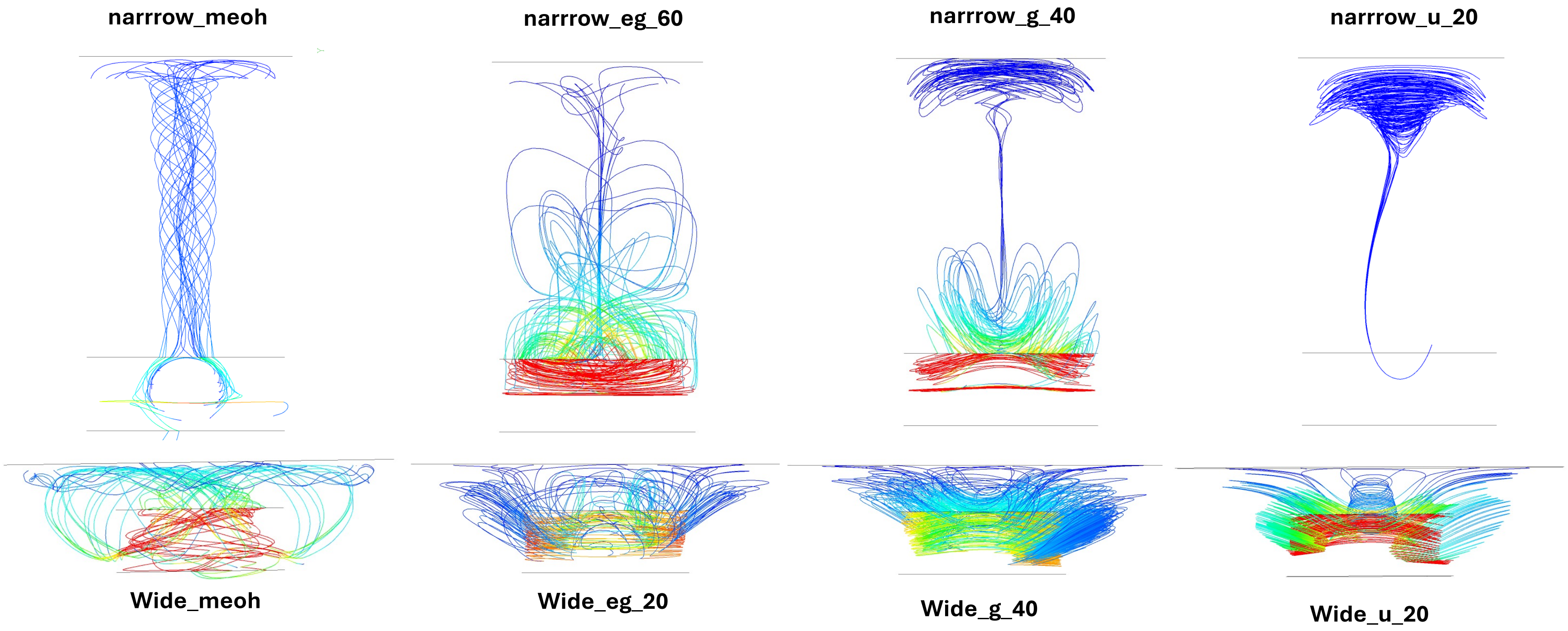

Figure S34: CFD streamlines for MeOH at 20 °C, ChCl:EG at 60 °C, ChCl:G at 40 °C, and ChCl:U at 20 °C, showing laminar characteristics as viscosity increases.
